# Autistic Adult Services Availability, Preferences, and User Experiences: Results From the Autism Spectrum Disorder in the European Union Survey

**DOI:** 10.3389/fpsyt.2022.919234

**Published:** 2022-06-10

**Authors:** Martina Micai, Francesca Fulceri, Tommaso Salvitti, Giovanna Romano, Luise Poustka, Robert Diehm, Georgi Iskrov, Rumen Stefanov, Quentin Guillon, Bernadette Rogé, Anthony Staines, Mary Rose Sweeney, Andrew Martin Boilson, Thora Leósdóttir, Evald Saemundsen, Irma Moilanen, Hanna Ebeling, Anneli Yliherva, Mika Gissler, Tarja Parviainen, Pekka Tani, Rafal Kawa, Eva Pisula, Astrid Vicente, Célia Rasga, Magdalena Budişteanu, Ian Dale, Carol Povey, Noelia Flores, Cristina Jenaro, Maria Luisa Monroy, Patricia García Primo, Tony Charman, Susanne Cramer, Christine Kloster Warberg, Ricardo Canal-Bedia, Manuel Posada, Diana Schendel, Maria Luisa Scattoni

**Affiliations:** ^1^Istituto Superiore di Sanità, Research Coordination and Support Service, Rome, Italy; ^2^Directorate General of Health Prevention, Ministry of Health, Rome, Italy; ^3^University Medical Center Göttingen, Department of Child and Adolescent Psychiatry and Psychotherapy, Göttingen, Germany; ^4^Department of Child and Adolescent Psychiatry and Psychotherapy, Medical University of Vienna, Vienna, Austria; ^5^Institute for Rare Diseases, Plovdiv, Bulgaria; ^6^Department of Social Medicine and Public Health, Faculty of Public Health, Medical University of Plovdiv, Plovdiv, Bulgaria; ^7^CERPPS, Université Toulouse Jean Jaurès, Toulouse, France; ^8^School of Nursing, Psychotherapy and Community Health, Dublin City University, Dublin, Ireland; ^9^The State Diagnostic and Counselling Centre, Kópavogur, Iceland; ^10^Clinic of Child Psychiatry, University Hospital of Oulu, Oulu, Finland; ^11^Oulu University Hospital, Medical Faculty, Oulu, Finland; ^12^Logopedic Child Language Research Center, University of Oulu, Oulu, Finland; ^13^Department of Knowledge Brokers, Finnish Institute for Health and Welfare, Helsinki, Finland; ^14^Research Centre for Child Psychiatry, University of Turku, Turku, Finland; ^15^Department of Molecular Medicine and Surgery and Region Stockholm, Academic Primary Health Care Centre, Karolinska Institute, Stockholm, Sweden; ^16^Finnish Association for Autism and Asperger’s Syndrome, Helsinki, Finland; ^17^Department of Psychiatry, University of Helsinki, Helsinki, Finland; ^18^Faculty of Psychology, University of Warsaw, Warsaw, Poland; ^19^Center for Biodiversity, Functional and Integrative Genomics, Instituto Nacional de Saúde Doutor Ricardo Jorge, Lisbon, Portugal; ^20^“Victor Babeş” National Institute for Research and Development in Pathology and Biomedical Sciences, Bucharest, Romania; ^21^National Autistic Society, The Centre for Autism, London, United Kingdom; ^22^Departamento de Personalidad, Evaluación y Tratamiento Psicológicos Salamanca, Instituto Universitario de Integración en la Comunidad, University of Salamanca, Salamanca, Spain; ^23^Departamento de Psicología Evolutiva y de la Educación, Instituto Universitario de Integración en la Comunidad, University of Salamanca, Salamanca, Spain; ^24^Instituto de Salud Carlos III, Institute of Rare Diseases Research, Madrid, Spain; ^25^King’s College London, Institute of Psychiatry, London, United Kingdom; ^26^Department of Public Health, Aarhus University, Aarhus, Denmark; ^27^Lundbeck Foundation Initiative for Integrative Psychiatric Research, Aarhus, Denmark; ^28^Department of Economics and Business, National Centre for Register-Based Research, Aarhus University, Aarhus, Denmark

**Keywords:** autism spectrum disorder, adults, residential service, employment service, education service, financial service, social service

## Abstract

There is very little knowledge regarding autistic adult services, practices, and delivery. The study objective was to improve understanding of current services and practices for autistic adults and opportunities for improvement as part of the Autism Spectrum Disorder in the European Union (ASDEU) project. Separate survey versions were created for autistic adults, carers of autistic adults, and professionals in adult services. 2,009 persons responded to the survey and 1,085 (54%) of them completed at least one of the services sections: 469 autistic adults (65% female; 55% <35 years old), 441 carers of autistic adults (27% female; 6% <35 years old), 175 professionals in adult services (76% female; 67% in non-medical services). Top choices by autistic adults, carers or professionals for services best suiting their current needs were: residential services: “help in own home” (adults, carers of high independent adults, professionals), “fulltime residential facility” (carers of low independent adults); employment services: “job mentors” (adults, carers of high independent adults, professionals), “Sheltered employment” (carers of low independent adults); education services: “support in regular education setting” (all groups); financial services: financial support in lieu of employment (“Supplementary income for persons unable to have full employment” for adults, “full pension” for carers of low independent adults) or to supplement employment earnings for carers of high independent adults and professionals; social services: “behavior training” (adults) and “life skills training” (carers and professionals). Waiting times for specific services were generally < 1 month or 1–3 months, except for residential services which could be up to 6 months; most professionals were uninformed of waiting times (>50% responded “don’t know”). Five of seven residential services features recommended for autistic adults were experienced by <50% of adults. The knowledge of good local services models that work well for autistic adults was generally low across all services areas. The variation in services experiences and perceptions reported by autistic adults, carers, or professionals underscore the need to query all groups for a complete picture of community services availability and needs. The results showed areas for potential improvement in autistic adult services delivery in the EU to achieve recommended standards.

## Introduction

Autism is characterized by deficits in social communication and interaction, and restricted/repetitive repertoires of behaviors, interests, and activities [Autism Spectrum Disorder, (1)] and usually is a lifelong condition (2–4). Despite the growing population of autistic youth aging into adulthood as well as newly diagnosed autistic adults, most studies on service use have been conducted investigating autistic children or young adults up until their late twenties (5, 6). In general, the research base in autistic adult services is underdeveloped which hampers efforts toward improvement of services provision and policymaking.

In adulthood, autistic persons often face challenges around services, such as lack of autism training of service providers and chaotic services management or, alternatively, having to pay for private services. Consequently, autistic adults or carers of autistic adults tend to express dissatisfaction with post-diagnostic support, interventions, and management of medical and psychiatric co-occurring conditions (7–15). A recent study explored autism service satisfaction and preferences of parents/guardians and autistic adults in Canada, France, Germany, Italy, and the United States (16) and the investigators found high rates of satisfaction for autism-specific early intervention and general day services but general dissatisfaction for job training and mixed-disability day services. Dissatisfaction could be one consequence of poor alignment between autistic services recommendations and actual experiences by users that has been reported in a few studies (8, 15, 17). Also, very few studies have explored the views of adult services by professionals versus autistic adults and carers [e.g., (15)].

The overall objective of the present study was to improve understanding of current services experienced by autistic adults and opportunities for improvement as part of the Autism Spectrum Disorder in the European Union (ASDEU) project. Specific study objectives were to examine perceptions and experiences of autistic adults, carers, and professionals on a variety of features of the overall services infrastructure (residential, employment, education, financial, social services) for autistic adults including: availability of public (versus private) services; whether the service they received was designed for autism specifically; what services were received versus what is perceived as most needed now; indicators of limited services availability (i.e., services waiting times); level of autism expertise in offices where services were applied for; alignment of user experiences of residential services with published guidelines for residential services; and users’ awareness of good services models across all services types.

## Materials and Methods

### Survey Development and Description

The ASDEU project conducted a survey on services based, in part, on a variety of published guidelines and recommendations regarding services for autistic adults [(18–21); Think Autism: Updating the 2010 Adult Autism Strategy]. The three versions of the survey targeted autistic adults; family/caregivers of autistic adults (NOT necessarily the carers of the adults who participated in this study themselves); and administrators/professionals/service providers for adults. Experts in all ASDEU sites reviewed the surveys and an autistic adult tested the adult version of the on-line survey. Written instructions were presented to the participants before they filled out the survey. Responders were asked to select answer choices that seemed to suit most closely with what they knew or had experienced and to answer to the best of their knowledge and experience. The survey questions were written using everyday language and avoided technical terms that might not be understood or not applicable across different countries. To ensure the reported information was recent, for each services section, only respondents who had applied for or had the service in the last 2 years were eligible to answer the section questions.

The present study used the following data: (1) demographic characteristics of responders, including 12 questions for the autistic adults, 9 for carers, and 7 for professionals; (2) residential services for autistic adults, including 11 questions for autistic adults, 11 for carers, and 6 for professionals; (3) employment services for autistic adults including 10 questions for autistic adults, 10 for carers, and 5 for professionals; (4) adult education services for autistic adults including 10 questions for autistic adults, 10 for carers, and 5 for professionals; (5) financial services for autistic adults including 10 questions for autistic adults, 10 for carers, and 5 for professionals; (6) social support services for autistic adults including 10 questions for autistic adults, 10 for carers, and 5 for professionals (Supplementary Data 1). For some of the questions regarding residential services provisions, the response options were designed to determine if the respondent’s experiences with local services matched published recommendations (Supplementary Data 10). Supplementary Data 1 presents the survey questions and response options for the demographic characteristics and specific services questions examined for this study.

When asked to report availability and preference for services, the survey had two sets of questions for different services situations: whether a respondent had applied for a specific type of service in the last 2 years and (a) failed to get it or (b) received it. Respondents were then asked what type of service would best suit their current needs.

### Recruitment and Survey Distribution

All ASDEU partners sent out survey invitations to participate to autism organizations (national, local, and voluntary) and service provider organizations (public and private, including residential facilities, job training, and education programs). Furthermore, these organizations were asked to share the survey links through their channels (e-newsletters, websites, or social media accounts). Also, the investigators at each site disseminated the surveys through their professional networks and on social media.

The survey was available online over 10.5 months in 2017. In mid-February, it was launched in three languages (English, Spanish, and Danish) and by mid-September 2017, in eight additional languages (French, Polish, Icelandic, German, Finnish, Italian, and Romanian, as well as Portuguese for the professional version); data for the analysis were collected until December 2017.

Each ASDEU site obtained local ethical approval before distributing the survey in their respective countries. All procedures were in accordance with the ethical standards of the institutional and/or national research committee. Responders read the information about the survey prior to start and gave their informed consent electronically. Personal identifying information was not collected. Data were analyzed in aggregated form.

### Analysis Methods

The entire survey was completed or partially completed by 2,009 participants distributed as follows: 667 autistic adults, 591 carers of autistic adults, and 751 professionals. We excluded the 3.63% (*n* = 73, 21 autistic adults, 16 carers, and 9 professionals) of responders who partially completed the survey, resulting in a sample size of 1,963 responders (646 autistic adults, 575 carers, and 742 professionals). The response rate was 97.7%, minimizing the risk for non-response bias.

For the present study, only demographic characteristics, and responses specific to residential, educational, employment, financial or social services for autistic adults were analyzed. Other survey sections on autistic adults’ diagnosis, health, and interventions are presented elsewhere (12, 13, 15).

For these analyses, distribution of responses (frequency, percent) from all three respondent groups regarding features of each type of service were analyzed separately; responses from the carer group for analysis were stratified on level of independence of the autistic adult under their care; for the questions on availability and preferences of services, responses from the autistic adult group were stratified on gender (Supplementary Data 4B–8C); we performed Chi-square tests (with Yates continuity corrections) on the affirmative answers to explore if autistic males vs. females vs. other gender/no gender answer differed in reporting the preference of services (Supplementary Data 4C–8C). We also repeated analyses of select questions – Have you tried (for adults)/Has the adult, or someone for the adult (for carers) tried to get a service at some time in the last 2 years? – to compare different countries of residence in terms of success of service availability/provision. For the questions on services, the number of respondents varied across the different service sections. A summary of the main findings is reported in Table 1.

**TABLE 1:** Summary of the main findings.

Autistic adult	Carer	Professional
**Demographic characteristics**		
Mostly women, 65%	Autistic adults cared for by carers mainly men, 72%	Mostly women, 76%
Mostly less than 35 years of age, 55%	Mostly less than 35 years of age, 81%	NA
Primarily living in Denmark, 41%	Primarily living in Denmark, 27%	Primarily living in Denmark, 54%
	Autistic adults cared for by carers had hig or some level of independence, 50%	Most non-medical background, 69%
**Organization of services**		
Most applied for a residential service at a public office	Most applied for a residential service at a public office	NA
**Availability and preferences of services**		
**Residential**		
14% tried to get a residential service at some time in the last 2 years but failed	24% tried to get a residential service at some time in the last 2 years but failed	Most reported having knowledge of and work experience in residential services, 79%
29% were in a residential service now or had been at some time in the last 2 years	48% were in a residential service now or had been at some time in the last 2 years	“Help while living in a college or school dormitory” was reported less often as available, 42%
40% were satisfied with the residential service they currently had	31% carers of HI and 23% of LI were satisfied with the residential service they currently had	NA
“Help in own home” was the most frequent residential service got, failed, and needed	“Help in own home” was the most frequent residential service got, failed, and needed”; “full time residential facility” was the most frequent residential service got and failed for carers of LI	“Help in own home” and “full time residential facility with full apartment” were the most needed residential services
**Employment**		
12% tried to get an employment service at some time in the last 2 years but failed	13% tried to get an employment service at some time in the last 2 years but failed	About half of professionals reported having knowledge of and work experience in employment services
28% had an employment service now or had been at some time in the last 2 years	29% had an employment service now or had been at some time in the last 2 years	All services options except “employer programs to encourage employment of persons with autism” (19%), were reported to be available in the area by 44–77%
24% were satisfied with the employment service they currently had	20% carers of HI and 20% of LI were satisfied with the employment service they currently had	NA
“Specific counseling” was the most frequent employment service failed and “Internships or work placement” the most frequent got	“Internship or work placement” was the most frequent employment service failed and got for carers of HI; “Job mentors” was the most frequent employment service failed and “Sheltered employment and job mentors” was the most frequent employment service got for carers of LI	NA
“Job mentors” and “job placement specifically for autistic adults” were the service that would suit them best now (i.e., not satisfied with current service)	“Job mentors” and “job placement specifically for autistic adults” were the service that would suit them best now (i.e., not satisfied with current service) for carers of HI; “sheltered employment” and “job mentors” for LI	“Job mentors” and “job placement specific for autistic persons/employer programs to encourage employment of persons with autism” were the most needed employment services
**Education**		
6% tried to get an education service at some time in the last 2 years but failed	12% tried to get an education service at some time in the last 2 years but failed	37% reported having knowledge of and work experience in education services
14% had an education service now or had been at some time in the last 2 years	29% had an education service now or had been at some time in the last 2 years	All services options were claimed to be currently available by >65% of respondents except for “boarding school or college for adults with autism spectrum” (35%)
35% were satisfied with the education service they currently had	29% carers of HI and 34% of LI were satisfied with the education service they currently had	NA
“Mentorship or specialist support in a regular education setting” and “day school or college for adults with autism” were the most frequent education service got, failed, and needed	“Mentorship or specialist support in a regular education setting” and “day school or college for adults with autism” were the most frequent residential service got, failed, and needed	“Mentorship or specialist support in regular education settings” and “day school or college for adults with autism spectrum” were the most needed education services
**Financial**		
18% tried to get a financial service at some time in the last 2 years but failed	22% tried to get a financial service at some time in the last 2 years but failed	25% reported having knowledge of and work experience in financial services
43% had a financial service now or had been at some time in the last 2 years	64% had a financial service now or had been at some time in the last 2 years	All services options were claimed to be currently available by >51% of respondents except for “special insurance to help pay for health care” (28%)
28% were satisfied with the financial service they currently had	21% carers of HI and 23% of LI were satisfied with the financial service they currently had	NA
“Supplementary income” was the most frequent financial service failed and “unemployment benefits” the most frequent got	“Support during school or job training” was the most frequent financial service failed for carers of HI; “caregiver supplementary income” for carers of LI; “full pension” was the most frequent financial service failed and got for carers of HI and LI	NA
“Supplementary income for persons unable to have full employment” and “full pension” were the most needed financial services	“Supported employment” was the most frequent financial service needed for HI; “full pension” for LI	“Supported employment” and “supplementary income for persons unable to have full employment” were the most needed financial services
**Social support**		
12% tried to get a social support service at some time in the last 2 years but failed	25% tried to get a social support service at some time in the last 2 years but failed	57% reported having knowledge of and work experience in social support services
31% had a social support service now or had been at some time in the last 2 years	36% had a social support service now or had been at some time in the last 2 years	“Life skills training” and “free time activities” were the most frequently selected options to be currently available
25% were satisfied with the social support service they currently had	12% carers of HI and 18% of LI were satisfied with the social support service they currently had	NA
“Free time activities” was the most frequent social support service failed and “life skill training” the most frequent got	“Free time activities” was the most frequent social support service failed for carers of HI; “life skill training” for carers of LI; “life skill training” was the most frequent social support service failed and got for carers of HI and LI, together with “free time activities” only for LI carers	NA
“Behavior training, for an individual,” “peer to peer matching,” “peer-to non-peer matching,” and “life skills training” were the most needed social support services	“Peer-to non-peer matching” and “life skills training” were the most frequent social support service needed for HI; “life skills training,” “behavior training for an individual,” and “peer-to non-peer matching” for LI	“Life skills training,” “free time activities,” and “behavior training for individuals” were the most needed social support services
**Alignment of user’s experience with residential services guidelines**		
<50% of autistic adults experienced: ways to get specialist care when needed, services coordination, activities to feel part of the community, the physical environment was adapted to their needs, and staff had specialist autism training	<50% of carers experienced: ways to get specialist care when needed, services coordination, support for employment, and support for independent living	Foreach recommended provision of residential services 63% of professionals said that it was in place or coming
**Waiting times**		
For the most services < 1 month or 1–3 months. Waiting times for residential services were more disparate and the time choices were more variable across the different types of residential services	For the most services < 1 month or 1–3 months. Waiting times for residential services were more disparate and the time choices were more variable across the different types of residential services	For the most part, did not know the waiting times for services and when they did report a waiting time it generally was not concordant with the adults’ and carers’ reports
**Straff training**		
<50% of autistic adults said that the staff of the office where to apply for services seemed knowledgeable about autism or autism services.	<50% of carers said that the staff of the office where to apply for services seemed knowledgeable about autism or autism services.	NA
50% or more said that only some or none of the staff seemed knowledgeable about autism spectrum or autism spectrum services	50% or more said that only some or none of the staff seemed knowledgeable about autism spectrum or autism spectrum services	NA
At the residential facility, 47% said that staff had special training in autism	At the residential facility, 59% said that staff had special training in autism	At the residential facility, 79% said that staff had special training in autism
**Good local models**		
<34% of adults knew of a good local service model. The domains with the lowest proportions of “yes” among adults were financial service, employment service, and adult education service	<34% of carers knew of a good local service model. The domain with the lowest proportions of “yes” among carers was financial service	Higher proportions of professionals knew of good local models, although for financial and social support service < 50% of professionals knew of a good local model
The domain with the highest proportions of “yes” for a good local model was the residential service	The domain with the highest proportions of “yes” for a good local model was the residential service	The domain with the highest proportions of “yes” for a good local model was the residential service

*NA = Question not available for the correspondent group. HI, high independent adults; LI, low independent adults.*

## Results

### Demographic Characteristics

For the demographic characteristics analysis, we considered responders to at least one of the services sections (*n* = 1,085). Responders were mostly women (autistic women: 308, 65%; carers: 361, 81%; professionals: 133, 76%), while the autistic adults cared for by carers were mainly men (319, 72%). Over half of the autistic adults (55%) were less than 35 years of age and 81% of the carers’ adults were less than 35. Responders were primarily living in Denmark (410, 37%), France (147, 13%), Spain (125, 11%), Finland (109, 10%), Poland (88, 8%), Italy (79, 7%), and Iceland (71, 6%) and lived in cities that are not capital cities (758, 69%) (Supplementary Data 2).

Most of the autistic adult responders reported to be currently in a college/university education program (65, 61%) or had completed study at a college/university level (149, 40%). Over half (247, 52%) of the autistic adult responders were unemployed, and the most common reason for unemployment was having a disability that prevents them from having a job (80, 32%). Only 20% of the autistic responders were diagnosed between 16 and 25 years old, while the rest were 26 years of age or older when diagnosed (Supplementary Data 2).

About half of autistic adults cared for by carers had some level of independence (high level of independence, 42, 9%; some independence but needs support, 180, 40%), whereas the other half required a high level of support (needs a high level of support in daily living, 149, 33%; needs high level institution-like care, 70, 15%). Fifty-eight percent (*n* = 42) of the autistic adults cared for by carers were diagnosed with autism between 16 and 25 years old, while the rest were 26 years of age or older when diagnosed (Supplementary Data 2).

The most represented backgrounds of the professionals were teachers/pedagogues (45, 25%), social workers (44, 25%), and psychologists (20, 11%); 31% (*n* = 55) of professionals selected the option “Other” when asked to select their professional background. Most (69%) of the professionals had a non-medical background (Supplementary Data 2).

### Organization of Services

Most adults and carers (60%) applied for service at a public office; almost all financial services applications (90%) were made at a public office. A large minority of social services applications (19.7% by autistic adults and 14.5% by carers) were made at charitable organizations (Supplementary Data 3A).

For employment, education or financial services, most adults and carers received the service from organizations NOT set up for autism specifically (largest percentages of respondents received the service at organizations for either all persons or persons with disabilities; autistic adults: 28.9% to 45.2%; carers: 18.5% to 71.9%) (Supplementary Data 3B). For residential and social services, however, 40% of adults and 50% of carers received the service from organizations set up for autism specifically (Supplementary Data 3B).

### Availability of and Preferences for Services

#### Residential

**FIGURE 1:**
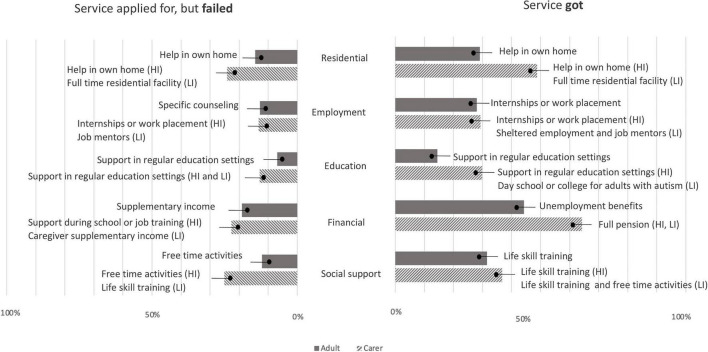
Services that had been applied for but failed to get or Services got reported by autistic adults and carers. Values expressed as percentages. Shown in the text boxes are the most frequently selected specific service option within each service area reported by autistic adults and carers of high independent adults (HI) and low independence adults (LI).

Among respondents, 14.5% of adults (48 of 331; 12.9% females; 18% males; 13.3% other gender/gender no answer), or someone for the adult, and 24.2% (55 of 227) of carers tried to get a residential service at some time in the last 2 years but failed while 29.1% (138 of 474) of adults and 48.8% (217 on 444) of carers reported that they were in a residential service now or had been at some time in the last 2 years (Figure 1 and Supplementary Data 4B). Autistic adults living in Italy (6, 16.6%) and Germany (1, 16.6%) and carers living in Finland (8, 22.2%) and Iceland (9, 22.5%) were those who have tried more often to get residential service and failed (Supplementary Data 9).

**FIGURE 2:**
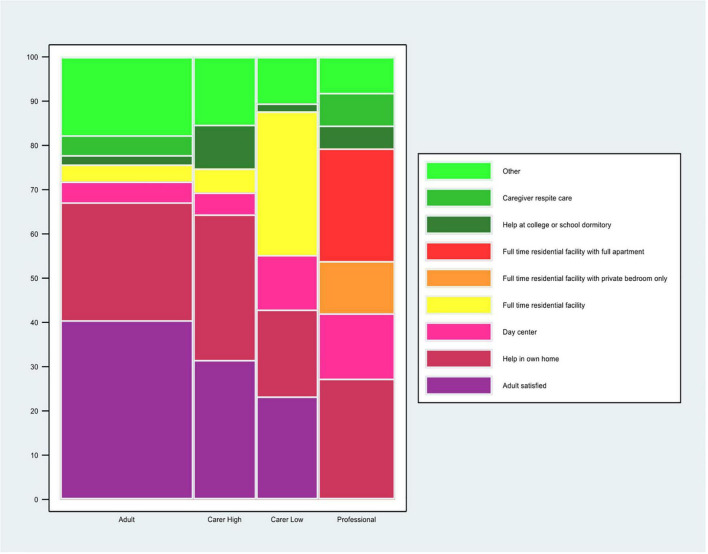
Preferred residential service. The question for the autistic adults was: “*If you could choose a residential service that fits your needs best now, what would you choose? (Please, tick 1 box).”* The question for the carers of autistic adults was: *“If you could choose a residential service that fits the adult’s needs best now, what would you choose? (Please, tick 1 box).”* The carers’ data are stratified by high and low level of independence of the autistic adult. The question for the professional was: *“Which 2 types of residential services do you think are most needed for autistic adults in the (geographical) area where you work now? (Please, tick 2 boxes).”* Professionals who selected “*I prefer not to make a choice*” are not included in the calculation of % for each of the other answer choices. The %s for the other answer choices are based on the professionals who made a choice, *n* = 271 (301–30 = 271). *This% is calculated on the total sample of the professionals’ responders (*n* = 301). Data are for all adults and carers of adults.

About 40.5% of adults but a lower percentage of carers (of high independence adults: 31.5%; of low independence adults: 23.9%) were satisfied with the residential service they currently had (Figure 2 and Supplementary Data 4A).

For autistic adult respondents, “help in own home” was by far the most frequent residential service: over 41.1% of adults who tried but failed to get a residential service in the last 2 years, were trying to get “help in own home”; 53.1% of adults who had a residential service in the last 2 years had “help in own home” service (Supplementary Data 4B). The most frequent choice by autistic adults for a residential service best suiting their needs now (i.e., the adults who were not satisfied with what they had already) was “help in own home” (26.6%) (Figure 2 and Supplementary Data 4A).

For the same three questions, the residential service profile of the adults of carers differed between adults of low and high independence. For carers of high independence adults, “help in own home” was the most common answer choice (Supplementary Data 4B – kind of residential service that was applied for, but failed: 34.2%; kind of residential service the adult had: 38.8%; residential service best suiting there needs now 32.8%), while “full time residential facility” was the most common answer choice when the adult was of low independence (Supplementary Data 4B – kind of residential service that was applied for, but failed: 26.0%; kind of residential service the adult got: 43.7%; Supplementary Data 4A – kind of service best suiting there needs now: 32.4%).

Similar distributions of responses on residential services availability and preferences were observed for autistic males and females (Supplementary Data 4C; *X ^2^* = 10.1, *p* = 0.60, Supplementary Data 4D).

Most professionals (177 of 224; 79%) reported having knowledge of and work experience in residential services that are currently available for adults, including autistic adults. Each residential service option was said to be available in the area by about 56.2–78.8% of professionals, except for “help while living in a college or school dormitory” which was said to be available by only 42.5% of professionals (Supplementary Data 4B). Consistent with the autistic adults and carers, the top two choices by professionals for residential services most needed in their area were “help in own home” as well as “full time residential facility with full apartment” (respectively selected by 27.3 and 25.4% of professionals) (Figure 2 and Supplementary Data 4A).

#### Employment

Among responders, 12.7% of the adults, or someone for the adult, (43 on 336; 12.5% females; 14.2% males; 7.1% other gender/no gender answer) and 13.3% (41 on 307) of carers tried to get an employment service at some time in the last 2 years but failed, while 28% (131 on 467) of the adults and the 29.3% (128 on 436) of the carers reported that had an employment service now or had been at some time in the last 2 years (Figure 1 and Supplementary Data 5B). Autistic adults living in Italy (8, 22.2%) and Germany (1, 16.6%) and carers living in Finland (5, 13.8%) were those who have tried more often to get employment service and failed (Supplementary Data 9).

**FIGURE 3:**
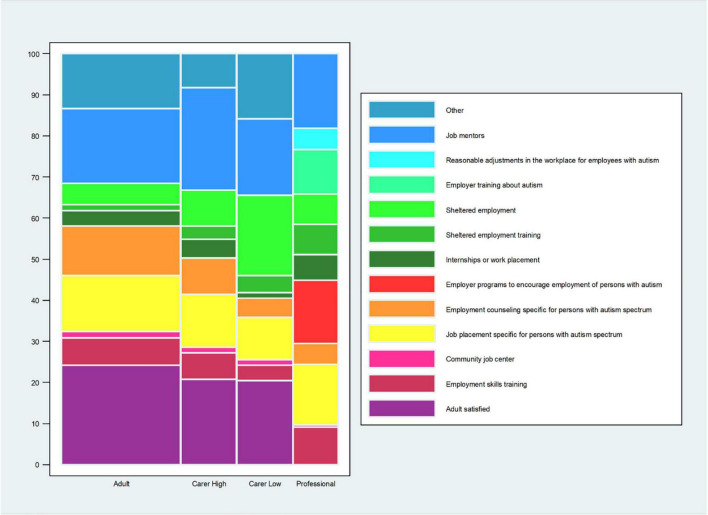
Preferred employment service. The question for the autistic adults was: “*If you could choose an employment service that fits your needs best now, what would you choose? (Please, tick 1 box).”* The question for the carers of autistic adults was: *“If you could choose an employment service that fits the adult’s needs best now, what would you choose? (Please, tick 1 box).”* The carers’ data are stratified by high and low level of independence of the autistic adult. The question for the professional was: *“Which 2 types of employment services do you think are most needed for autistic adults in the (geographical) area where you work now? (Please, tick 2 boxes).”* Professionals who selected “*I prefer not to make a choice*” are not included in the calculation of% for each of the other answer choices. The%s for the other answer choices are based on the professionals who made a choice, *n* = 176 (198–22 = 176). *This% is calculated on the total sample of the professionals’ responders (*n* = 198). Data are for all adults and carers of adults.

A quarter or less of adults (24.1%) and carers (of high independence adults: 20.7%; of low independence adults: 20.4%) were satisfied with the employment service they currently had (Figure 3 and Supplementary Data 5A).

For adults who tried to get a service and failed, the top two services they sought were “employment counseling specific for persons with autism spectrum” (17.1%) and “job mentors” (16.1%). For adults who had an employment service, the most common services they got were “internships or work placement” (21.6%) and “job mentors” (18.7%) (Supplementary Data 5B). When asked what service that would suit them best now (i.e., not satisfied with current service), the top choices by the adults were: “job mentors” (18.1%) and “job placement specifically for autistic adults” (13.5%) (Figure 3 and Supplementary Data 5A).

For carers, the top two choices differed by the adult’s level of independence. For a service that the carers tried to get and failed, carers of high independence adults were more often trying to get “internships or work placement” (16.6%) and “community job center” (14.2%) while carers of low independence adults were trying to get “job mentors” (26.8%) or “employment counseling specifically for autistic persons” (17%). For carers who had a service, the most frequent services the adult got among carers of high independence adults was “internships or work placement” (21.5%) and “job mentors” (19.6%) while carers of low independence adults got “sheltered employment” (18.1%) or “job mentors” (18.1%) (Supplementary Data 5B). For services that would suit best the adult now (i.e., not satisfied with current service), carers of high independence adults selected more often “job mentors” (24.8%) and “job placement specifically for autistic adults” (12.9%) while carers of low independence adults selected “sheltered employment” (19.5%) and “job mentors” (18.6%) Supplementary Data 5A).

Similar distributions of responses on employment services availability and preferences were observed for autistic males and females (Supplementary Data 5C; *X ^2^* = 14.7, *p* = 0.68, Supplementary Data 5D).

About half of the professionals reported having knowledge of and work experience in employment services that are currently available for adults, including autistic adults. For professionals, all of the 11 services options except “employer programs to encourage employment of persons with autism” (19.1%), were reported to be available in the area by 44–77% of professionals (Supplementary Data 5B). For professionals, the top two choices of services most needed now were: “job mentors” (18.1%) and “job placement specific for autistic persons/employer programs to encourage employment of persons with autism” (15.3%) (Figure 3 and Supplementary Data 5A).

#### Education

Among respondents, 6.8% of adults (27 of 393; 5.7% females; 10.4% males; 7.1% other gender/no gender answer), or someone for the adult, and 12.9% (39 of 302) of the carers tried but failed to get an adult education service at some time in the last 2 years; 14.5% (67 of 462) adults and 29.9% (129 of 431) of the carers reported that they had an adult education service now or at some time in the last 2 years (Figure 1 and Supplementary Data 6B). Autistic adults living in Italy (7, 19.4%) and Republic of Ireland (2, 15.3%) and carers living in Republic of Ireland (2, 20.0%), Finland (5, 13.8%), and France (9, 13.4%) were those who have tried more often to get education service and failed (Supplementary Data 9).

**FIGURE 4:**
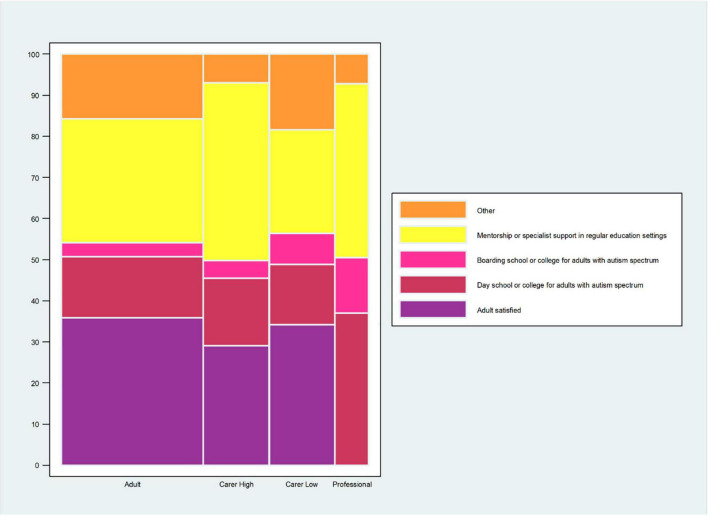
Preferred adult education service. The question for the autistic adults was: “*If you could choose an education service that fits your needs best now, what would you choose? (Please, tick 1 box).”* The question for the carers of autistic adults was: *“If you could choose an education service that fits the adult’s needs best now, what would you choose? (Please, tick 1 box).”* The carers’ data are stratified by high and low level of independence of the autistic adult. The question for the professional was: *“Which 2 types of education services do you think are most needed for autistic adults in the (geographical) area where you work now? (Please, tick 2 boxes).”* Professionals who selected “*I prefer not to make a choice*” are not included in the calculation of% for each of the other answer choices. The %s for the other answer choices are based on the professionals who made a choice, *n* = 111 (124–13 = 111). *This% is calculated on the total sample of the professionals’ responders (*n* = 124). Data are for all adults and carers of adults.

About a third of adults (35.8%) and carers (of high independence adults: 29.1%; of low independence adults: 34.1%) were satisfied with the education service they currently had (Figure 4 and Supplementary Data 6A).

For adults and carers, the most frequently selected education service option across all three questions (tried and failed; had a service; what service would best suit now) was “mentorship or specialist support in a regular education setting” while “day school or college for adults with autism” was consistently a close second choice. The option that was consistently least frequently selected across all 3 questions and all respondent groups was “boarding school or college for autistic adults” (Figure 4 and Supplementary Data 6A,B).

Similar distributions of responses on education services availability and preferences were observed for autistic male and female (Supplementary Data 6C; *X ^2^* = 9.2, *p* = 0.32, Supplementary Data 6D).

About a third (37%) of the professionals reported having knowledge of and work experience in adult education services that are currently available for adults, including autistic adults. For professionals, all services options were claimed to be currently available by >65% of respondents except for “boarding school or college for adults with autism spectrum” (only 35.5% of professionals said it was currently available) (Supplementary Data 6B). For professionals, the 2 options most frequently selected as most needed now were “mentorship or specialist support in regular education settings” (42.3%) and “day school or college for adults with autism spectrum” (36.9%) (Figure 4 and Supplementary Data 6A).

#### Financial

Among respondents, 18.9% of adults (49 of 258; 18.5% females; 22% males), or someone for the adult, and 22.6% (34 of 150) of the carers tried and failed to get a financial service at some time in the last 2 years while 43.2% (197 of 455) of the adults and 64.2% (270 of 420) of the carers reported that they had financial service now or had one at some time in the last 2 years (Figure 1). Autistic adults living in Spain (5, 22.7%) and France (13, 21.3%) and carers living in Republic of Ireland (1, 10.0%), Denmark (12, 9.8%) and Italy (3, 9.3%) were those who have tried more often to get financial service and failed (Supplementary Data 9).

**FIGURE 5:**
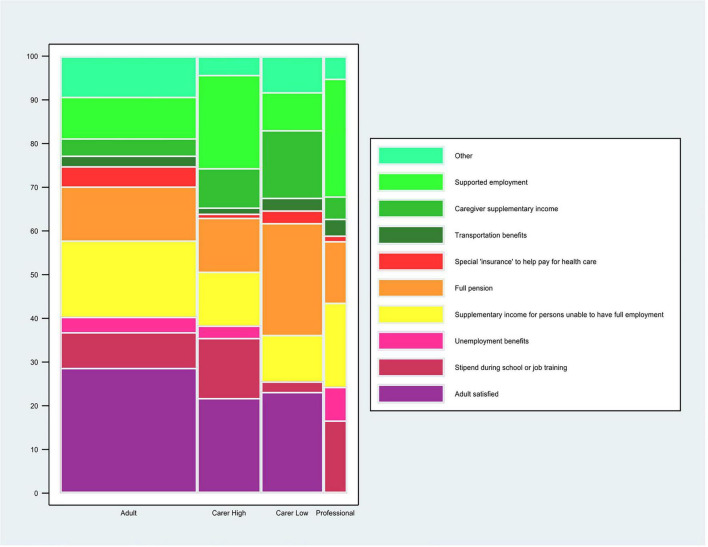
Preferred financial service. The question for the autistic adults was: “*If you could choose a financial service that fits your needs best now, what would you choose? (Please, tick 1 box).”* The question for the carers of autistic adults was: *“If you could choose a financial service that fits the adult’s needs best now, what would you choose? (Please, tick 1 box).”* The carers’ data are stratified by high and low level of independence of the autistic adult. The question for the professional was: *“Which 2 types of financial services do you think are most needed for autistic adults in the (geographical) area where you work now? (Please, tick 2 boxes).”* Professionals who selected “*I prefer not to make a choice*” are not included in the calculation of% for each of the other answer choices. The %s for the other answer choices are based on the professionals who made a choice, *n* = 78 (88–10 = 78). *This% is calculated on the total sample of the professionals’ responders (*n* = 88). Data are for all adults and carers of adults.

About a third of adults (28.7%) but fewer carers (of high independence adults: 21.8%; of low independence adults: 23.1%) were satisfied with the financial service they currently had (Figure 5 and Supplementary Data 7A).

For adults, the most frequently selected financial service options that they tried to get and failed were “supplementary income for persons unable to have full employment” (18.4%) or “full pension” (13.1%) (Supplementary Data 7B). For the service the adult got the most frequent options were “unemployment benefits” (22.3%) and “full pension” (16.2%) (Supplementary Data 7B). The most frequent options that would best suit their needs now were “supplementary income for persons unable to have full employment” (17.4%) or “full pension” (12.3%) (Figure 5 and Supplementary Data 7A).

For carers the selected options differed depending on the level of their adult’s independence. For carers of high independence adults, the top selected option for a financial service they tried to get and failed was “stipend/support during school or job training” (26%) while for carers of low independence adults “caregiver supplementary income” (42.8%) was the top choice. The most frequently selected financial service option across questions for a service that the adult got was “full pension” for both carers of high independence adults (19.5%) and low independence adults (39.2%) (Supplementary Data 7B). For carers of high independence adults, the most frequent option that would best suit their needs now was “supported employment” (21.3%) and for carers of low independence adults “full pension” (25.6%) (Supplementary Data 7A).

Gender analysis showed a significant difference in preference responses for financial services (*X ^2^* = 7.4, *p* = 0.005, Supplementary Data 7D). About 20% of females preferred “Supplementary income for persons unable to have full employment,” while only 12.1% of males selected this option. Whereas “Supported employment” was more often selected by males (14.9%) than females (7.1%; Supplementary Data 7D).

About a quarter of the (25.6%) professionals reported having knowledge of and work experience in financial services that are currently available for adults, including autistic adults. For these professionals, all services options were claimed to be currently available by >51% of respondents except for “special insurance to help pay for health care” (only 28.8% of professionals said it was currently available; Supplementary Data 7B). For professionals, the 2 options most frequently selected as most needed were “supported employment” (26.9%) and “supplementary income for persons unable to have full employment” (19.2%) (Figure 5 and Supplementary Data 7A).

#### Social Support

Among responders, 12% of adults (37 of 306; 12.3% females; 11.0% males; 16.6% other gender/no gender answer), or someone for the adult, and 25% (66 of 263) of the carers tried and failed to get a social support service at some time in the last 2 years; 31.5% (142 of 450) of the adults and 36.7% (153 of 416) of the carers reported that had a social support service now or had one at some time in the last 2 years (Figure 1). Autistic adults living in France (10, 16.3%) and Spain (3, 13.6%) and carers living in Iceland (10, 25.0%), Italy (7, 21.8%), Republic of Ireland (2, 20.0%), and France (13, 19.4%) were those that have tried more often to get social support service and failed (Supplementary Data 9).

**FIGURE 6:**
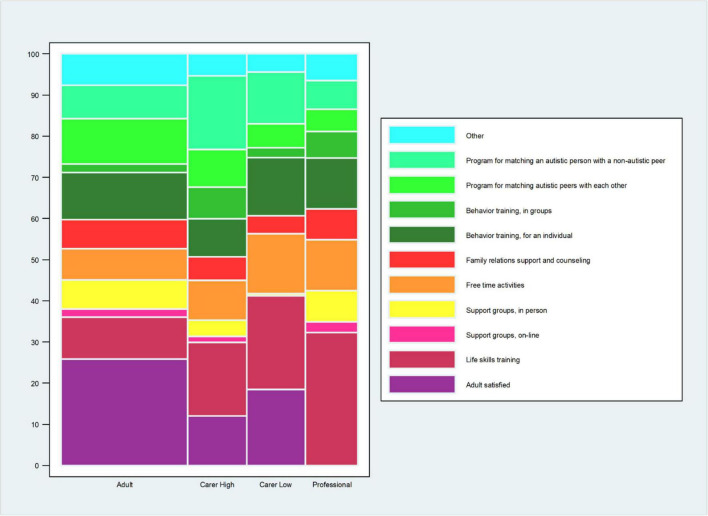
Preferred social support service. Data are for all adults and carers of adults. The question for the autistic adults was: “*If you could choose a social support service that fits your needs best now, what would you choose? (Please, tick 1 box).”* The question for the carers of autistic adults was: *“If you could choose a social support service that fits the adult’s needs best now, what would you choose? (Please, tick 1 box).”* The carers’ data are stratified by high and low level of independence of the autistic adult. The question for the professional was: *“Which 2 types of social support services do you think are most needed for autistic adults in the (geographical) area where you work now? (Please, tick 2 boxes).”* Professionals who selected “*I prefer not to make a choice*” are not included in the calculation of% for each of the other answer choices. The %s for the other answer choices are based on the professionals who made a choice, *n* = 186 (207–21 = 186). *This% is calculated on the total sample of the professionals’ responders (*n* = 207).

About a third of adults (25.9%) but fewer carers (of high independence adults: 12%; of low independence adults: 18.4%) were satisfied with the social support service they currently had (Figure 6 and Supplementary Data 8A).

For both adults and carers - of both high and low independence adults - the two most frequently selected choices for social services they tried to get and failed, or currently have, were: “life skills training” and “free time activities” (Supplementary Data 8B). In contrast, when asked what services options would suit them best now, adults selected “behavior training, for an individual” and “peer to peer matching” (11.4%), as well as “peer-to non-peer matching” (11.0%) and “life skills training” (10.1%) (Supplementary Data 8A). For carers of high independence adults, their top choices for this question were “peer-to non-peer matching” as well as “life skills training” (17.8%). For carers of low independence adults, their top two choices were “life skills training” (22.8%), “free time activities” (14.5%) in addition to “behavior training for an individual” (14.0%) and “peer-to non-peer matching” (12.6%) (Supplementary Data 8A).

Similar distribution of responses on social support services availability and preferences were observed for autistic males and females (Supplementary Data 8C; *X ^2^* = 28.2, *p* = 0.10, Supplementary Data 8D).

Over half (57.2%) of the professionals reported having knowledge of and work experience in social support services that are currently available for adults, including autistic adults. The same two service types selected by autistic adults (tried to get and failed, or currently have: “life skills training” and “free time activities”) were by far the most frequently selected options to be currently available by the professionals (both said to be currently available by 80% of professionals) (Supplementary Data 8B). The professionals’ choices for what was most needed was consistent with that of the adults and carers: “life skills training” (by far the most frequently selected option at 32.2% of professionals), “free time activities” and “behavior training for individuals” (12.3%; Figure 6 and Supplementary Data 8A).

### Alignment of User’s Experience With Residential Services Guidelines

For each recommended provision of residential services (i.e., structured activities for the residents, activities for the residents to feel part of the community, opportunities for the residents to go into different places in the community, a physical environment that is adapted to the needs of adults with autism spectrum, staff with specialist autism spectrum training, ways to get specialist care when it is needed, ways to coordinate services with other providers in the area, if needed, support for employment, support for independent living), 63% of professionals said that it was in place or coming.

Somewhat lower proportions of adults and carers reported that they experienced each recommended service provision with their residential service. More than 50% of carers reported: structured activities (63.4%), staff had specialist autism training (59.2%), the adult had opportunities to go into the community (58.8%), physical environment was adapted to the adult’s needs (58.8%), and the adult had activities to feel part of the community (55%) (Supplementary Data 10).

However, less than 50% of autistic adults or carers experienced: ways to get specialist care when needed (adults: 18.2%, carers: 44.9%), services coordination (adults: 32.1%, carers: 40.7%), support for employment (carers only, 37.5%), and support for independent living (carers only, 44.4%). Additionally, less than 50% of adults experienced the following (although many also said the item was not applicable to them): activities to feel part of the community (39.4%), the physical environment was adapted to their needs (38.6%), and staff had specialist autism training (47.4%) (Supplementary Data 10).

### Waiting Times

Adults’ and carers’ reports regarding waiting times for employment, education, financial and social services were very highly concordant and were for the most part < 1 month or 1–3 months. Adults’ and carers’ reports regarding waiting times for residential services, however, were more disparate and the time choices were more variable across the different types of residential services. Professionals, for the most part, did not know the waiting times for services (50% or more selected “don’t know”) and when they did report a waiting time it generally was not concordant with the adults’ and carers’ reports (Supplementary Data 11).

### Staff Training

At the offices where autistic adults and carers went to apply for a given service (residential, employment, education, financial, or social), less than 50% of both groups said that the staff seemed knowledgeable about autism or autism services. On the other hand, 50% or more said that only some or none of the staff seemed knowledgeable about autism spectrum or autism spectrum services.

At the residential facility itself, 47.4% of adults and 59.2% of carers said that staff had special training in autism (Supplementary Data 12), while 79% of professionals answered “yes” to this feature: Do you have knowledge of and work experience in residential services that are currently available for adults, including autistic adults?

### Good Local Models

Across the five different services domains, the survey included a question asking the respondent if they knew of a good local model for the service, for example, in the residential service area, employment or education for autistic adults. Supplementary Data 13 presents the results from these questions, summarized over all five domains. Across each of the five service area domains, generally less than 34.7% of adults and carers knew of a good local service model. The domain with the lowest proportions of “yes” among adults (9.9%) and carers (14.6%) was financial service; employment service (9.5%) and adult education service (10%) were also low among autistic adults. Higher proportions of professionals knew of good local models, although for two of the five domains less than 50% of professionals knew of a good local model (financial service: 36.5%; social support service: 47.4%). The domain with the highest proportions of “yes” for a good local model was the residential service domain (autistic adults: 17.48%; carers: 34.7%; professionals: 72.6%). Another striking feature was that large proportions of respondents answered “don’t know” to the questions, indicating that knowledge of good local models of service is not high, even among professionals (Supplementary Data 13).

## Discussion

The availability of autism services and unmet needs should be tracked to inform ongoing, coordinated system actions where service users and providers are key and active players in the process. In this study, we asked autistic adults, carers, and professionals about what they want, have, or need regarding services. The study results inform our understanding of several general features of the services infrastructure for autistic adults with the added advantage of looking at a variety of service types. For example, adults and carers were most likely (over 60%) to apply for a service at a public office although, according to the majority of respondents, the staff at the organizations where they applied for a given service did not seem knowledgeable about autism. The employment, education, and financial services they received were typically NOT set up for autistic persons specifically while residential and social services most likely were. These results should be noted as a gap in the service provision and are reinforced by the Cascio and Racine (16) results where most respondents preferred autism-specific services, especially for autism-specific support groups, residential services, and social/recreational groups as well as autism-specific early intervention and general day services.

While all service types had been sought for or received by large proportions of adults or carers (although < 50% in each case) in the last 2 years, the largest proportions of respondents had sought (and failed) or received financial services.

Answers on success of service provision, varied considerably across different countries and difficulties emerged in specific countries. For example, responders living in Italy reported more often that they had tried to get a service and failed (autistic adults: residential, employment, and education services; carers: financial and social support services). Responders living in France reported this challenge for financial and social support services (autistic adults), education and social support services (carers). Responders living in the Republic of Ireland reported this challenge for education (autistic adults and carers), financial and social support services (carers). Sample sizes by country were also quite variable, which could be contributing to the variability in the results. Uneven sample size across different countries with different health/social care systems means that the overall summary mean averages may not apply more broadly across Europe and we need to know more about access to health and social care provision in the countries underrepresented in the current survey.

There were fairly consistent results across all respondent groups in terms of the specific services which they had sought for, received, or would best suit them now, e.g., “help in own home” among the different type of residential services, whereas respondents’ satisfaction with their current service varied widely by type of service and respondent group: the highest proportion was found among the autistic adults regarding satisfaction with their residential service and the lowest was found among carers of high independence adults regarding satisfaction with their social services. There were very similar distributions of responses by gender of the autistic adults regarding services availability and preferences, except in the case of financial services.

### Residential Services

Forty percent of the autistic adults were satisfied with their current residential situation and “help in own home” was by far the most common and preferred residential service choice for autistic adults. Availability and preference/need for residential services of carers appeared to depend in part on the level of independence of the adult: “help in home” for high independence and “full time residential facility” for carers of low independence adults. The proportion of carers satisfied with what they had was low (31% for high independent adults; 23% low independence adults). Perhaps reflecting an under-met need, professionals also most often selected “help in own home” as well as “full time residential facility with full apartment” as the residential service most needed. In the United States (US), it has been observed that it is more common for autistic adults to live in a family member’s home and less common to live in agency apartments, in their own home or an “other living arrangement” (22). Nevertheless, in a long-term prospective follow-up study of a population-based cohort (*n* = 120), very few families (*n* = 3, 3.6%) wanted to keep their loved ones at home as long as possible (23). An interview study suggested that what was considered “best” depended on the family member in question: mothers perceived that the adult living in the family home was the best option for the family, while living in a residential facility was the best arrangement for the autistic adult (24). From the adult’s family perspective, 12 families (13%) in the Billstedt et al. (23) study reported the need for respite care “to cope with the situation, to provide a welcome break, to help with transition from home and to enable the individual and his or her family to get used to separations” (23).

### Employment Services

For adults and carers, “job mentors” was the employment service most likely to be selected across the different questions of employment service availability or preference/need. According to the professionals, the service option that was least likely to be currently available was “job placement specific for autistic persons/employer programs to encourage employment of persons with autism,” while the top choice for what was most needed was “job mentors.” Other studies have reported the importance of “worksite peer mentors” who are constantly present at work to help the autistic adult with social interactions and other problems (25–27) (versus job mentors who are not always present at the job and may be less knowledgeable about work requirements and social interactions/environment; (25)). Another study highlighted the success of a close cooperation between the autistic adult job coaches and employers in exploring targeted job opportunities and finding job duties appropriate to the adult’s abilities (28).

### Adult Education Services

For adults and carers, “mentorship or specialist support in a regular education setting” was the education service most likely to be selected across the different questions of education service availability or preference/need. For carers of low independence adults, “day school or college for adults with autism” was the education service most likely to be selected among the kind of adult educational service the adult got. Along similar lines, the service options that the professionals reported were selected as top choices for what was most needed were “mentorship or specialist support in regular education settings” and “day school or college for adults with autism spectrum.” The preferences for mentorship in regular education settings or autism-specific facilities likely reflect the support needs to address challenges of autistic students in higher education settings that require increasing independence, organization and time management, social relationships, unexpected changes, and sensory and academic demands (29–36). Other investigators have advocated for autism spectrum-specific support provided by higher education organizations (37, 38).

### Financial Services

For adults and carers, the most common financial services of choice seemed to concern financial support in lieu of employment (“full pension”) or to supplement employment earnings, either by the adult or carers, or for support during education/job training. For professionals, the top choices for needed financial services also revolved around employment: “supported employment” or “supplementary income for persons unable to have full employment.” The focus in these results on financial support due to no or limited earnings from employment is interesting in view of a recent literature review of the high costs associated with autism which highlighted a considerable array of potential costs: medical and healthcare service costs, therapeutic costs, (special) education costs, costs of informal care by family/caregivers, costs of accommodation, respite care, and out-of-pocket expenses, as well as costs of lost productivity by the adult or family/caregivers (39). Thus, perhaps the shortfalls in income from employment that limit the ability to pay for their diverse needs are a main financial concern of autistic adults or their carers. This concern is reflected regardless of the gender of the autistic respondent.

### Social Support Services

Although “life skills training” and “free time activities” were consistently chosen by most adults, carers, and professionals across the questions of social services availability and preference/need, another top preference was “behavior training for an individual” and, for carers of higher independence adults, different forms of “peer to peer matching.” While during adolescence, autistic people may show increased interest in social relationships and in developing social skills [e.g., (40, 41)], most individuals continue to show social impairment in adolescence and adulthood (42, 43). This study’s results appear to underscore the perceived need to improve poor social skills and to have targeted social skills services integrated into the care pathway of autistic adults.

### Alignment With Residential Services’ Guidelines

The National Institute for Health and Care Excellence autism guidelines [(21), updated in 2021] advises that the residential environment should be “structured to support and maintain a collaborative approach between the autistic person and their family, partner or carer(s) for the development and maintenance of interpersonal and community living skills” (21). The NICE guidelines also concern the residential care activities, care environments and care staff characteristics. The present study investigated the user and professionals’ experiences around these aspects.

From the perspective of the adults and carers in this study, only 2 of 8 recommendations were experienced by the majority (>50%) of the adults and only 5 of 10 recommendations were experienced by the majority (>50%) of carers: structured activities for the residents and opportunities for the residents to go into different places in the community (for adults and carers), activities for the residents to feel part of the community, a physical environment that is adapted to the needs of adults with autism spectrum, staff with specialist autism spectrum training (for carers). The other recommended services provisions experienced by less than half of respondents were “ways to get specialist care when needed,” “services coordination,” “support for employment,” “support for independent living,” “activities to feel part of the community,” “residential physical environment was adapted to their needs,” and “residential staff had specialist autism training.” The present results parallel the findings of Scattoni et al. (15) where a lack of alignment was observed between the user experiences and guidelines on recommended characteristics for post-diagnostic support for autistic adults (15).

### Waiting Times for Services

According to the adult and carer responses, waiting times for residential services seemed to be more variable than waiting times for employment, education, financial, or social services; the waiting times for the latter were for the most part less than 1 month or 1 to 3 months. Professional respondents were not well informed about waiting times for these types of services (majority answering “Don’t know”). There is little literature on services for autistic adults waiting times; the available literature focuses on child services waiting times. Caldwell and Heller (44) reported that families on waiting lists for services for developmental disabilities showed more unmet needs and lower service satisfaction than families supported by services. If longer waiting times indicate both a large demand and limited supply of the service, then this study’s results indicate a most limited availability for residential services for autistic adults that appears to be larger than that for other types of services.

Professionals, for the most part, reported that they did not know the waiting times for services and when they did report a waiting time it generally was not in parallel with the users’ reports. Users should be accompanied by an autism-trained care manager through the process of requesting and waiting for residential service - which should be as short as possible. In addition, professionals should be aware of the time that users and their families spend awaiting care so that they can best accommodate them in the service.

### Service Staff Training

The perception of more than half of autistic adults and carers was that only some or none of the employees in offices where services are applied for seemed knowledgeable about autism or autism services. The value of staff training was supported by a study of McDonnell et al. (45) that showed that a 3-day training course increased residential social care and day service staff confidence in managing aggression in autistic people. The present study appears to indicate considerable shortfalls in training of services staff for autistic adults even though staff training improves service quality for the clients and benefits the staff persons themselves. It is particularly important that employees in the offices where autistic adults and their carers apply for services know about autism and its needs in adulthood so that they can best refer and advise them about the services available locally.

### Good Local Service Models

Although knowledge of good local services models that work well for autistic adults was generally low across all services areas, residential services had the highest proportions of respondents with positive responses. Large proportions of respondents answered “don’t know” to the questions, indicating that knowledge of good local models of service is not high, even among professionals. These results underscore both the complex nature of the services infrastructure needed for autistic adults and opportunities for improvement.

This generally low positive response rate may reflect a possible lack of good services models for autistic adults in the local community or, possibly, a critical information gap among both users and professionals. It is recommended that local care pathways are understandable, accessible, acceptable for users and providers, consider the person’s knowledge and understanding of autism and its care and be appropriate to the local communities [(21), updated in 2021; 2014], and relevant professionals should know local autism care pathways and the way to access services (46).

### Limitations

The present results should be interpreted in view of the study limitations. First, the survey data were collected by convenience sampling thus it is likely that there may be selection bias since only people already in a support network and with internet access had access to the survey. Second, most of the autistic adult respondents were female [as found in on-line surveys in general; (47)], thus the results may poorly reflect the autistic males’ experiences and preferences. Third, most of the responders were living in cities that are not capital cities having <1.000, 1.000–20.000 or 20.000–100.000 inhabitants, thus the results may apply to specific contexts, with less services availability than found in capital cities. Fourth, the survey did not ask for important clinical information from the responders (e.g., the psychosocial functioning, illness severity or current treatment) apart from the question for the carers regarding level of independence of the autistic adult asked of the carers. Future studies should seek to ask participants for more detailed information about their clinical background to determine the impact on services use. Fifth, age specific analysis (e.g., different experiences with service options by age at diagnosis) was not feasible due to sample size limitations, especially because data were also stratified by gender. Finally, although we considered the level of independence of the carer’s autistic adult, we could not fully account for the developmental level of the autistic adult which is a strong predictor of service usage and needs (48).

## Conclusion

The ASDEU survey sheds light on the state of different types of autistic adult services in 11 countries of the European Union from the perspective of autistic adults, carers and professionals. The results underscore the highly diverse needs and preferences of the autistic community such that the different residential, employment, educational, financial, and social services options that adults or their carers tried to get and failed, or succeeded in receiving, or believed would better suit their current needs varied depending on the respondent group and the level of independence of the autistic adult in question. Although there was some concordance between professional and adult/carer responses in terms of the availability and preferences/needs of different services options, the differences are also important to note since they highlight the different perspectives of the professional providers versus the adult/carer services users. Thus, for services policy planning, it is important to consider the perspectives of all these types of respondents when assessing autistic adult services needs and gaps in the community (49).

The results also highlight specific gaps in knowledge: lack of autism knowledge and training in services office staff, shortfalls in meeting published recommendations around residential services (which may reflect lack of knowledge about them by the service provider), professionals’ lack of awareness of services waiting times, and lack of knowledge by all respondent groups of good local services models that work well for autistic adults. Filling these knowledge gaps may be important steps toward more equitable service delivery and better support for the autistic adult community.

## Data Availability Statement

The datasets presented in this article are not readily available because we can only share aggregated data (not individual level), and any data based on a small sample size (<5) may not be shared as an extra data privacy precaution. Requests to access the datasets should be directed to DS, diana.schendel@ph.au.dk and MS, marialuisa.scattoni@iss.it.

## Ethics Statement

The studies involving human participants were reviewed and approved by Each ASDEU site obtained local ethical approval. The patients/participants provided their written informed consent to participate in this study.

## Author Contributions

MLS, TS, MM, and DS: formal analysis. MM, MLS, and DS: writing – original draft preparation. MP: funding acquisition for the survey dissemination and data collection and analysis (DGSANCO) and principal investigator. MLS: funding acquisition for the Italian participation at the survey, data analysis and writing (“Osservatorio Italiano per il monitoraggio dei disturbi dello spettro autistico” and “I disturbi dello spettro autistico: attività previste dal decreto ministeriale del 30.12.2016”) and principal investigator. All authors contributed to the conceptualization, investigation, and writing – review and editing and have read and agreed to the published version of the manuscript.

## Conflict of Interest

The authors declare that the research was conducted in the absence of any commercial or financial relationships that could be construed as a potential conflict of interest.

## Publisher’s Note

All claims expressed in this article are solely those of the authors and do not necessarily represent those of their affiliated organizations, or those of the publisher, the editors and the reviewers. Any product that may be evaluated in this article, or claim that may be made by its manufacturer, is not guaranteed or endorsed by the publisher.
