# Supplementary material for: Autistic Adult Services Availability, Preferences, and User Experiences: Results From the Autism Spectrum Disorder in the European Union Survey
Source: Front Psychiatry. 2022 Jun 10;13:919234. doi: 10.3389/fpsyt.2022.919234 (PMC9226363; doi:10.3389/fpsyt.2022.919234)
Supplement: Supplementary file 1 [file Table_1.DOC]

Supplementary Material

# Supplementary Data 1

Survey’s questions and answer choices for autistic adults, carers and professionals

| Question autistic adult | Question carer | Question professional | Answer autistic adult | Answer carer | Answer professional |
| --- | --- | --- | --- | --- | --- |
| Demographic characteristics | | | | | |
| N/A | N/A | Thinking of your work history, what job or career title fits you best? | N/A | N/A | General practitioner |
|  |  |  |  |  | Psychiatrist |
|  |  |  |  |  | Medical specialist, other than psychiatrist |
|  |  |  |  |  | Nurse |
|  |  |  |  |  | Other medical professional |
|  |  |  |  |  | Psychologist |
|  |  |  |  |  | Social worker |
|  |  |  |  |  | Teacher/pedagogue |
|  |  |  |  |  | Teaching assistant/nursery assistant |
|  |  |  |  |  | Mental health therapist |
|  |  |  |  |  | Physical or occupational therapist |
|  |  |  |  |  | I work in the area of criminal justice (e.g., police, courts, legal advocate) |
|  |  |  |  |  | Other, please specify |
| N/A | N/A | Thinking of your work history, how many years in total have you been in jobs in adult services and care (social, medical, or other services) | N/A | N/A | < 1 year |
|  |  |  |  |  | 1-2 years |
|  |  |  |  |  | 3-5 years |
|  |  |  |  |  | 6-10 years |
|  |  |  |  |  | > 10 years |
| What is your gender? | What is your gender? | What is your gender? | Male | Male | Male |
|  |  |  | Female | Female | Female |
|  |  |  | Other/no answer | Other/no answer | Other/no answer |
| How old are you? | How old are you? | N/A | 18-25 | 18-25 | 18-25 |
|  |  |  | 26-35 | 26-35 | 26-35 |
|  |  |  | 36-45 | 36-45 | 36-45 |
|  |  |  | 46-55 | 46-55 | 46-55 |
|  |  |  | 56-64 | 56-64 | 56-64 |
|  |  |  | > 65 | > 65 | > 65 |
| N/A | How many years of education did you complete? | N/A | N/A | < 10 years | N/A |
|  |  |  |  | 10-12 years |  |
|  |  |  |  | 13-16 years |  |
|  |  |  |  | > 16 years |  |
| Are you going to a school, a home school or in an education program now? | N/A | N/A | Yes, full time | N/A | N/A |
|  |  |  | Yes, part time |  |  |
|  |  |  | No |  |  |
| If no, do you remember your age when you finished your education? | N/A | N/A | Yes | N/A | N/A |
|  |  |  | No |  |  |
| How old were you when you finished your education? | N/A | N/A | _______ | N/A | N/A |
| If yes, what kind of education are you in now? | N/A | N/A | Primary level school | N/A | N/A |
|  |  |  | Secondary level school (for example high school or gymnasium) |  |  |
|  |  |  | Technical, vocational or job training school |  |  |
|  |  |  | College or university |  |  |
| What kind of education were you in when you finished your education: | N/A | N/A | Secondary - level school (for example high school or gymnasium) | N/A | N/A |
|  |  |  | Technical, vocational or job training school |  |  |
|  |  |  | College or university |  |  |
|  |  |  | Don't know |  |  |
|  |  |  | Secondary - level school (for example high school or gymnasium) |  |  |
| Are you: | What is your current employment status now? Pick the answer that fits you best. | N/A | N/A | Student | N/A |
|  |  |  |  | Unemployed |  |
|  |  |  |  | Employed (part time or full time) |  |
|  |  |  |  | Self-employed |  |
|  |  |  |  | Retired |  |
|  |  |  |  | Volunteer |  |
| If you are unemployed, is it because: | N/A | N/A | You are a student | N/A | N/A |
|  |  |  | You are retired |  |  |
|  |  |  | You are looking for a job |  |  |
|  |  |  | You believe that you cannot find a job |  |  |
|  |  |  | You have a disability that prevents you from having a job |  |  |
|  |  |  | Other, please specify |  |  |
| What country do you live in? | What country do you live in? | What country do you work in? | Austria | Austria | Austria |
|  |  |  | Belgium | Belgium | Belgium |
|  |  |  | Bulgaria | Bulgaria | Bulgaria |
|  |  |  | Denmark | Denmark | Denmark |
|  |  |  | England | England | England |
|  |  |  | Finland | Finland | Finland |
|  |  |  | France | France | France |
|  |  |  | Iceland | Iceland | Iceland |
|  |  |  | Italy | Italy | Italy |
|  |  |  | Northern Ireland | Northern Ireland | Northern Ireland |
|  |  |  | Poland | Poland | Poland |
|  |  |  | Portugal | Portugal | Portugal |
|  |  |  | Republic of Ireland | Republic of Ireland | Republic of Ireland |
|  |  |  | Romania | Romania | Romania |
|  |  |  | Scotland | Scotland | Scotland |
|  |  |  | Spain | Spain | Spain |
|  |  |  | Wales | Wales | Wales |
|  |  |  | Other, specify | Other, specify | Other, specify |
| N/A | What is the autistic adult’s gender? | N/A | N/A | Male | N/A |
|  |  |  |  | Female |  |
|  |  |  |  | Other/no answer |  |
| N/A | How old is the autistic adult? | N/A | N/A | 18-25 | N/A |
|  |  |  |  | 26-35 |  |
|  |  |  |  | 36-45 |  |
|  |  |  |  | 46-55 |  |
|  |  |  |  | 56-64 |  |
|  |  |  |  | > 65 |  |
| N/A | How many years have you known the autistic adult? | N/A | N/A | Less than 1 year | N/A |
|  |  |  |  | 1-5 years |  |
|  |  |  |  | 5-10 years |  |
|  |  |  |  | More than 10 years but not the adult's whole life |  |
|  |  |  |  | The adult's whole life |  |
| N/A | How are you related to the autistic adult? | N/A | N/A | Parent | N/A |
|  |  |  |  | Other family member related by blood (not a parent, but for example a child, cousin, grand parent, uncle etc.) |  |
|  |  |  |  | Spouse or partner |  |
|  |  |  |  | A carer, but not a family member, spouse or partner |  |
| Where do you live now? | Where is the autistic adult living now? | Thinking of your current job location where is it located? | Capital city | Capital city | Capital city |
|  |  |  | Other than a capital city | Other than a capital city | Other than a capital city |
| How many people live in the community where you live now? (Answer the best you can) | How many people live in the community where the autistic adult lives now? Answer as best you can | How many people live in the community where your current job is located? | < 1.000 people | < 1.000 people | < 1.000 people |
|  |  |  | 1.000-20.000 people | 1.000-20.000 people | 1.000-20.000 people |
|  |  |  | 20.000-100.000 people | 20.000-100.000 people | 20.000-100.000 people |
|  |  |  | 100.000-1.000.000 | 100.000-1.000.000 | 100.000-1.000.000 |
|  |  |  | > 1.000.000 | > 1.000.000 | > 1.000.000 |
|  |  |  | Don't Know | Don't Know | Don't Know |
| N/A | Pick the description that best fits the autistic adult. | N/A | N/A | Has a high level of independence | N/A |
|  |  |  |  | Has some independence but needs support |  |
|  |  |  |  | Needs a high level of support in daily living |  |
|  |  |  |  | Needs high level institution-like care |  |
| N/A | N/A | The experience and knowledge about services for adults that you have from your current job is: | N/A | N/A | Most closely connected to your current job location (e.g., capital city or small town) |
|  |  |  |  |  | Most closely connected to your current job location and a wider area (e.g., the region or state where your job is located) |
|  |  |  |  |  | Most closely connected to the whole country |
| Residential services | | | | | |
| Are you in a residential service now or have been at some time in the last 2 years? | Is the adult in a residential service now or has been at some time in the last 2 years? | Do you have knowledge of and work experience in residential services that are currently available for adults, including autistic adults? | Yes | Yes | Yes |
|  |  |  | No | No | No |
|  |  |  |  | Don’t know |  |
| Have you tried to get a residential service at some time in the last 2 years? | Has the adult, or someone for the adult, tried to get a residential service at some time in the last 2 years? | N/A | Yes, I have tried to get the service, and I did NOT succeed | Yes, the adult, or someone for the adult, tried to get the service, but did NOT succeed | N/A |
|  |  |  | No, I have not tried to get the service | No, nobody has tried to get the service |  |
| If yes, what kind of residential service were you trying to get? (Check all that apply) If you have tried to get a residential service more than one time, please think of the most recent time that you tried. | If yes, what kind of residential service did the adult, or someone for the adult, try to get?  (Check all that apply)  If the adult, or someone for the adult, has tried to get a residential service more than one time, please think of the most recent time it was tried. | For each type of adult residential service available for autistic persons, select the availability that best fits what you know. | Help in own home | Help in own home | Help in own home (Currently available; Not available now, but there are plans in motion to make it available; Not available now, but it is available in other areas of the country; Not available now and, to my knowledge, there are no plans to make it available; Don’t know) |
|  |  |  | Day center (a place a person goes to during the day and returns home at night) | Day center (a place a person goes to during the day and returns home at night) | Day center (a place a person goes to during the day and returns home at night) (Currently available; Not available now, but there are plans in motion to make it available; Not available now, but it is available in other areas of the country; Not available now and, to my knowledge, there are no plans to make it available; Don’t know) |
|  |  |  | Full time residential facility | Full time residential facility | N/A |
|  |  |  | N/A | N/A | Full time residential facility with private bedroom only (Currently available; Not available now, but there are plans in motion to make it available; Not available now, but it is available in other areas of the country; Not available now and, to my knowledge, there are no plans to make it available; Don’t know) |
|  |  |  | N/A | N/A | Full time residential facility with full apartment (Currently available; Not available now, but there are plans in motion to make it available; Not available now, but it is available in other areas of the country; Not available now and, to my knowledge, there are no plans to make it available; Don’t know) |
|  |  |  | Help while living in a college or school dormitory | Help while living in a college or school dormitory | Help while living in a college or school dormitory (Currently available; Not available now, but there are plans in motion to make it available; Not available now, but it is available in other areas of the country; Not available now and, to my knowledge, there are no plans to make it available; Don’t know) |
|  |  |  | Caregiver respite care | Caregiver respite care | Caregiver respite care (Currently available; Not available now, but there are plans in motion to make it available; Not available now, but it is available in other areas of the country; Not available now and, to my knowledge, there are no plans to make it available; Don’t know) |
|  |  |  | Other, please specify | Other, please specify | N/A |
| What kind of office or organization did you go to in order to apply for your residential service? (Check all that apply)  If you have applied for a residential service more than one time, please think of the most recent time that you applied. | What kind of office or organization was used to apply for the adult’s residential service? (Check all that apply)  If the adult, or someone for the adult, has applied for a residential service more than one time, please think of the most recent time that it was applied for. | N/A | Public office or organization | Public office or organization | N/A |
|  |  |  | Private office or organization | Private office or organization | N/A |
|  |  |  | Charity-, advocacy- or volunteer-based organization | Charity-, advocacy- or volunteer-based organization | N/A |
|  |  |  | Don't know | Don't know | N/A |
| At the organization, did the employees that worked with you to apply for residential services: | At the organization, did the employees that worked with the adult to apply for residential services: | N/A | Seem knowledgeable about autism spectrum? (Yes; Some were and some were not; No; Don't know) | Seem knowledgeable about autism spectrum? (Yes; Some were and some were not; No; Don't know) | N/A |
|  |  |  | Seem knowledgeable about the kinds of services and care that were available for you? (Yes; Some were and some were not; No; Don't know) | Seem knowledgeable about the kinds of services and care that were available for adults with autism spectrum? (Yes; Some were and some were not; No; Don't know) | N/A |
|  |  |  | Provide information about care and services that was easy for you to understand? (Yes; Some were and some were not; No; Don't know) | Provide information about care and services that was easy to understand by the autistic adult? (Yes; Some were and some were not; No; Don't know) | N/A |
| What kind of residential service did you get? (Check all that apply) | What kind of residential service did the adult get? (Check all that apply) |  | Help in own home | Help in own home | N/A |
|  |  |  | Day center (a place a person goes to during the day and returns home at night) | Day center (a place a person goes to during the day and returns home at night) | N/A |
|  |  |  | Full time residential facility | Full time residential facility | N/A |
|  |  |  | Help while living in a college or school dormitory | Help while living in a college or school dormitory | N/A |
|  |  |  | Caregiver respite care | Caregiver respite care | N/A |
|  |  |  | Other, please specify | Other, please specify | N/A |
| Was the residential service set-up for: (check all that apply) | Was the residential service set-up for (Check all that apply) | N/A | All adults | All adults | N/A |
|  |  |  | Adults with handicaps or special needs, specifically | Adults with handicaps or special needs, specifically | N/A |
|  |  |  | Adults with autism spectrum, specifically | Adults with autism spectrum, specifically | N/A |
|  |  |  | Don’t know | Don’t know | N/A |
|  |  |  | Other, please specify | N/A | N/A |
| What was the waiting time for you to get into the residential service? | What was the waiting time for the adult to get into the residential service? | N/A | < 1 month | < 1 month | N/A |
|  |  |  | 1-3 months | 1-3 months | N/A |
|  |  |  | 3-6 months | 3-6 months | N/A |
|  |  |  | > 6 months | > 6 months | N/A |
|  |  |  | Don't know | Don't know | N/A |
| N/A | N/A | To the best of your knowledge, what is the waiting time before an autistic adult can get into a residential service in the "area where you work now"? | N/A | N/A | Help in own home (This service not currently available; < 1 month; 1-3 months; 3-6 months; > 6 months; Don't know) |
|  |  |  | N/A | N/A | Day center (a place a person goes to during the day and returns home at night) (This service not currently available; < 1 month; 1-3 months; 3-6 months; > 6 months; Don't know) |
|  |  |  | N/A | N/A | Full time residential facility with private bedroom only (This service not currently available; < 1 month; 1-3 months; 3-6 months; > 6 months; Don't know) |
|  |  |  | N/A | N/A | Full time residential facility with full apartment (This service not currently available; < 1 month; 1-3 months; 3-6 months; > 6 months; Don't know) |
|  |  |  | N/A | N/A | Help while living in a college or school dormitory (This service not currently available; < 1 month; 1-3 months; 3-6 months; > 6 months; Don't know) |
|  |  |  | N/A | N/A | Caregiver respite care (This service not currently available; < 1 month; 1-3 months; 3-6 months; > 6 months; Don't know) |
| Does or did the residential service provide: | To the best of your knowledge, does the adult’s residential service provide: | To the best of your knowledge, do the residential services for adults in the "area where you work now" provide: | Structured activities for you (Yes; No; Does not apply; Don’t know) | Structured activities for the residents (Yes; No; Does not apply; Don’t know) | Structured activities for the residents (Yes; No, but there are plans in motion to get it started; No, but I believe that it may be helpful; No, and I believe that it is not needed; Don’t know; Does not apply) |
|  |  |  | Activities for you to feel part of the community (Yes; No; Does not apply; Don’t know) | Activities for the residents to feel part of the community | Activities for the residents to feel part of the community (Yes; No, but there are plans in motion to get it started; No, but I believe that it may be helpful; No, and I believe that it is not needed; Don’t know; Does not apply) |
|  |  |  | Opportunities for you to go into different places in the community (Yes; No; Does not apply; Don’t know) | Opportunities for the residents to go into different places in the community | Opportunities for the residents to go into different places in the community (Yes; No, but there are plans in motion to get it started; No, but I believe that it may be helpful; No, and I believe that it is not needed; Don’t know; Does not apply) |
|  |  |  | A physical environment that was adapted to your needs (Yes; No; Does not apply; Don’t know) | A physical environment that is adapted to the needs of adults with autism spectrum (Yes; No; Does not apply; Don’t know) | A physical environment that is adapted to the needs of adults with autism spectrum (Yes; No, but there are plans in motion to get it started; No, but I believe that it may be helpful; No, and I believe that it is not needed; Don’t know; Does not apply) |
|  |  |  | Staff with specialist autism spectrum training (Yes; No; Does not apply; Don’t know) | Staff with specialist autism spectrum training (Yes; No; Does not apply; Don’t know) | Staff with specialist autism spectrum training (Yes; No, but there are plans in motion to get it started; No, but I believe that it may be helpful; No, and I believe that it is not needed; Don’t know; Does not apply) |
|  |  |  | Ways to get specialist care when it is needed (Yes; No; Does not apply; Don’t know) | Ways to get specialist care when it is needed (Yes; No; Does not apply; Don’t know) | Ways to get specialist care when it is needed (Yes; No, but there are plans in motion to get it started; No, but I believe that it may be helpful; No, and I believe that it is not needed; Don’t know; Does not apply) |
|  |  |  | Ways to coordinate other services you need with providers in the area (Yes; No; Does not apply; Don’t know) | Ways to coordinate services with other providers in the area, if needed (Yes; No; Does not apply; Don’t know) | Ways to coordinate services with other providers in the area (Yes; No, but there are plans in motion to get it started; No, but I believe that it may be helpful; No, and I believe that it is not needed; Don’t know; Does not apply) |
|  |  |  | N/A | Support for employment (Yes; No; Does not apply; Don’t know) | N/A |
|  |  |  | N/A | Support for independent living | N/A |
| If you could choose a residential service that fits your needs best now, what would you choose? (Please, tick 1 box) | If you could choose a residential service that fits the adult’s needs best now, what would you choose? (Please, tick 1 box) | Which 2 types of residential services do you think are most needed for autistic adults in the "area where you work now": (Please, tick 2 boxes). | I am satisfied with what I have now | The adult is satisfied with what he or she has | I prefer to not make a choice |
|  |  |  | Help in own home | Help in own home | Help in own home |
|  |  |  | Day center (a place a person goes to during the day and returns home at night) | Day center (a place a person goes to during the day and returns home at night) | Day center (a place a person goes to during the day and returns home at night) |
|  |  |  | Full time residential facility | Full time residential facility | N/A |
|  |  |  | N/A | N/A | Full time residential facility, private bedroom only |
|  |  |  | N/A | N/A | Full time residential facility, full apartment |
|  |  |  | Help while living in a college or school dormitory | Help while living in a college or school dormitory | Help while living in a college or school dormitory |
|  |  |  | Caregiver respite care | Caregiver respite care | Caregiver respite care |
|  |  |  | Other, please specify | Other, please specify | Other, please specify |
| Do you know of an adult residential service, in your area or elsewhere in your country, which works very well for autistic adults? | Do you know of an adult residential service, in your area or elsewhere in your country, which works very well for autistic adults? | Do you know of an adult residential service, in your area or elsewhere in your country, which works very well for autistic adults? | Yes | Yes | Yes |
|  |  |  | No | No | No |
|  |  |  | N/A | N/A | Don’t know |
| Employment services | | | | | |
| Are you in an employment service now or have been at some time in the last 2 years? | Is the adult in an employment service now or has been at some time in the last 2 years? | Do you have knowledge of and work experience in employment services that are currently available for adults, including autistic adults? | Yes | Yes | Yes |
|  |  |  | No | No | No |
|  |  |  | N/A | Don't know | N/A |
| Have you tried to get an employment service at some time in the last 2 years? | Has the adult, or someone for the adult, tried to get an employment service at some time in the last 2 years? | N/A | Yes, I have tried to get the service, and I did NOT succeed | Yes, the adult, or someone for the adult, tried to get the service, but did NOT succeed | N/A |
|  |  |  | No, I have not tried to get the service | No, nobody has tried to get the service | N/A |
| If yes, what kind of service were you trying to get? (Check all that apply)  If you have tried to get an employment service more than one time, please think of the most recent time that you tried. | If yes, what kind of employment service did the adult, or someone for the adult, try to get? (Check all that apply)  If the adult, or someone for the adult, has tried to get an employment service more than one time, please think of the most recent time it was tried. | For each type of adult employment service available for autistic persons, select the availability that best fits what you know. | Employment skills training | Employment skills training | Employment skills training (Currently available; Not available now, but there are plans in motion to make it available; Not available now, but it is available in other areas of the country; Not available now and, to my knowledge, there are no plans to make it available; Don't know) |
|  |  |  | Community job center (not specific for autism spectrum) | Community job center (not specific for autism spectrum) | Community job center (not specific for autism) (Currently available; Not available now, but there are plans in motion to make it available; Not available now, but it is available in other areas of the country; Not available now and, to my knowledge, there are no plans to make it available; Don't know) |
|  |  |  | Job placement specific for persons with autism spectrum | Job placement specific for persons with autism spectrum | Job placement specific for persons with autism (Currently available; Not available now, but there are plans in motion to make it available; Not available now, but it is available in other areas of the country; Not available now and, to my knowledge, there are no plans to make it available; Don't know) |
|  |  |  | Employment counseling specific for persons with autism spectrum | Employment counseling specific for persons with autism spectrum | Employment counseling specific for persons with autism (Currently available; Not available now, but there are plans in motion to make it available; Not available now, but it is available in other areas of the country; Not available now and, to my knowledge, there are no plans to make it available; Don't know) |
|  |  |  | N/A | N/A | Employer programs to encourage employment of persons with autism (Currently available; Not available now, but there are plans in motion to make it available; Not available now, but it is available in other areas of the country; Not available now and, to my knowledge, there are no plans to make it available; Don't know) |
|  |  |  | Internships or work placement | Internships or work placement | Internships or work placement (Currently available; Not available now, but there are plans in motion to make it available; Not available now, but it is available in other areas of the country; Not available now and, to my knowledge, there are no plans to make it available; Don't know) |
|  |  |  | Sheltered employment training (employment training in a separate setting to prepare persons for employment in a regular setting) | Sheltered employment training (employment training in a separate setting to prepare persons for employment in a regular setting) | Sheltered employment training (employment training in a separate setting to prepare persons for employment in a regular setting) (Currently available; Not available now, but there are plans in motion to make it available; Not available now, but it is available in other areas of the country; Not available now and, to my knowledge, there are no plans to make it available; Don't know) |
|  |  |  | Sheltered employment (employment in a separate setting for persons who cannot work in a regular work place) | Sheltered employment (employment in a separate setting for persons who cannot work in a regular work place) | Sheltered employment (employment in a separate setting for persons who cannot work in a regular work place) (Currently available; Not available now, but there are plans in motion to make it available; Not available now, but it is available in other areas of the country; Not available now and, to my knowledge, there are no plans to make it available; Don't know) |
|  |  |  | Job mentors (for example, a job coach or other special help at work) | Job mentors (for example, a job coach or other special help at work) | Job mentors (for example, a job coach or other special help at work) (Currently available; Not available now, but there are plans in motion to make it available; Not available now, but it is available in other areas of the country; Not available now and, to my knowledge, there are no plans to make it available; Don't know) |
|  |  |  | N/A | N/A | Employer training about autism (Currently available; Not available now, but there are plans in motion to make it available; Not available now, but it is available in other areas of the country; Not available now and, to my knowledge, there are no plans to make it available; Don't know) |
|  |  |  | N/A | N/A | Reasonable adjustments in the workplace for employees with autism (Currently available; Not available now, but there are plans in motion to make it available; Not available now, but it is available in other areas of the country; Not available now and, to my knowledge, there are no plans to make it available; Don't know) |
|  |  |  | Other, please specify | Other, please specify | N/A |
| What kind of office or organization did you go to in order to apply for your employment service? (Check all that apply)  If you have applied for an employment service more than one time, please think of the most recent time that you applied. | What kind of office or organization was used to apply for the adult’s employment service? (Check all that apply)  If the adult, or someone for the adult, has applied for an employment service more than one time, please think of the most recent time that it was applied for. | N/A | Public office or organization | Public office or organization | N/A |
|  |  |  | Private office or organization | Private office or organization | N/A |
|  |  |  | Charity-, advocacy- or volunteer-based organization | Charity-, advocacy- or volunteer-based organization | N/A |
|  |  |  | Don’t know | Don’t know | N/A |
| At the organization, did the employees that worked with you to apply for employment services: | At the organization, did the employees that worked with the adult to apply for employment services: | N/A | Seem knowledgeable about autism spectrum? (Yes; Some were and some were not; No; Don't know) | Seem knowledgeable about autism spectrum? (Yes; Some were and some were not; No; Don't know) | N/A |
|  |  |  | Seem knowledgeable about the kinds of services and care that were available for you? (Yes; Some were and some were not; No; Don't know) | Seem knowledgeable about the kinds of services and care that were available for adults with autism spectrum? (Yes; Some were and some were not; No; Don't know) | N/A |
|  |  |  | Provide information about care and services that was easy for you to understand? (Yes; Some were and some were not; No; Don't know) | Provide information about care and services that was easy to understand by the autistic adult? (Yes; Some were and some were not; No; Don't know) | N/A |
| What kind of employment service did you get? (Check all that apply) | What kind of employment service did the adult get? (Check all that apply) | N/A | Employment skills training | Employment skills training | N/A |
|  |  |  | Community job center (not specific for autism spectrum) | Community job center (not specific for autism spectrum) | N/A |
|  |  |  | Job placement specific for persons with autism spectrum | Job placement specific for persons with autism spectrum | N/A |
|  |  |  | Employment counseling specific for persons with autism spectrum | Employment counseling specific for persons with autism spectrum | N/A |
|  |  |  | Internships or work placement | Internships or work placement | N/A |
|  |  |  | Sheltered employment training (employment training in a separate setting to prepare persons for employment in a regular setting) | Sheltered employment training (employment training in a separate setting to prepare persons for employment in a regular setting) | N/A |
|  |  |  | Sheltered employment (employment in a separate setting for persons who cannot work in a regular work place) | Sheltered employment (employment in a separate setting for persons who cannot work in a regular work place) | N/A |
|  |  |  | Job mentors (for example, a job coach or other special help at work) | Job mentors (for example, a job coach or other special help at work) | N/A |
|  |  |  | Other, please specify | Other, please specify | N/A |
| Was the employment service set-up for: (Check all that apply) | Was the employment service set-up for: (Check all that apply) | N/A | All adults | All adults | N/A |
|  |  |  | Adults with handicaps or special needs, specifically | Adults with handicaps or special needs, specifically | N/A |
|  |  |  | Adults with autism spectrum, specifically | Adults with autism spectrum, specifically | N/A |
|  |  |  | Don’t know | Don’t know | N/A |
|  |  |  | Other, please specify | Other, please specify | N/A |
| What was the waiting time before you could get into the employment service? | What was the waiting time for the adult to get into the employment service? | N/A | < 1 month | < 1 month | N/A |
|  |  |  | 1-3 months | 1-3 months | N/A |
|  |  |  | 3-6 months | 3-6 months | N/A |
|  |  |  | > 6 months | > 6 months | N/A |
|  |  | N/A | Don't know | Don't know | N/A |
| N/A | N/A | To the best of your knowledge, what is the waiting time before an autistic adult can get into an employment service in the "area where you work now"? | N/A | N/A | Employment skills training (This service not currently available; < 1 month; 1-3 months; 3-6 months; > 6 months; Don't know) |
|  |  |  | N/A | N/A | Job placement specific for persons with autism (This service not currently available; < 1 month; 1-3 months; 3-6 months; > 6 months; Don't know) |
|  |  |  | N/A | N/A | Employment counseling specific for persons with autism (This service not currently available; < 1 month; 1-3 months; 3-6 months; > 6 months; Don't know) |
|  |  |  | N/A | N/A | Internships or work placement (This service not currently available; < 1 month; 1-3 months; 3-6 months; > 6 months; Don't know) |
|  |  |  | N/A | N/A | Sheltered employment training (employment training in a separate setting to prepare persons for employment in a regular setting) (This service not currently available; < 1 month; 1-3 months; 3-6 months; > 6 months; Don't know) |
|  |  |  | N/A | N/A | Sheltered employment (employment in a separate setting for persons who cannot work in a regular work place) (This service not currently available; < 1 month; 1-3 months; 3-6 months; > 6 months; Don't know) |
|  |  |  | N/A | N/A | Job mentors (for example, a job coach or other special help at work) (This service not currently available; < 1 month; 1-3 months; 3-6 months; > 6 months; Don't know) |
| If you could choose an employment service that fits your needs best now, what would you choose? (Please, tick 1 box) | If you could choose an employment service that fits the adult’s needs best now, what would you choose? (Please, tick 1 box) | Which 2 types of employment services do you think are most needed for autistic adults in the "area where you work now" (Please, tick 2 boxes). | I am satisfied with what I have now | The adult is satisfied with what he or she has | I prefer not to make a choice |
|  |  |  | Employment skills training | Employment skills training | Employment skills training |
|  |  |  | Community job center (not specific for autism spectrum) | Community job center (not specific for autism spectrum) | Community job center (not specific for autism spectrum) |
|  |  |  | Job placement specific for persons with autism spectrum | Job placement specific for persons with autism spectrum | Job placement specific for persons with autism spectrum |
|  |  |  | Employment counseling specific for persons with autism spectrum | Employment counseling specific for persons with autism spectrum | Employment counseling specific for persons with autism spectrum |
|  |  |  | N/A | N/A | Employer programs to encourage employment of persons with autism spectrum |
|  |  |  | Internships or work placement | Internships or work placement | Internships or work placement |
|  |  |  | Sheltered employment training (employment training in a separate setting to prepare persons for employment in a regular setting) | Sheltered employment training (employment training in a separate setting to prepare persons for employment in a regular setting) | Sheltered employment training (employment training in a separate setting to prepare persons for employment in a regular setting) |
|  |  |  | Sheltered employment (employment in a separate setting for persons who cannot work in a regular work place) | Sheltered employment (employment in a separate setting for persons who cannot work in a regular work place) | Sheltered employment (employment in a separate setting for persons who cannot work in a regular work place) |
|  |  |  | Job mentors (for example, a job coach or other special help at work) | Job mentors (for example, a job coach or other special help at work) | Job mentors (for example, a job coach or other special help at work) |
|  |  |  | N/A | N/A | Employer training about autism |
|  |  |  | N/A | N/A | Reasonable adjustments in the workplace for employees with autism spectrum |
|  |  |  | Other, please specify | Other, please specify | Other, please specify |
| Do you know of an employment service, in your area or elsewhere in your country, that works very well for autistic adults? | Do you know of an employment service, in your area or elsewhere in your country, which works very well for autistic adults? | Do you know of an employment service for adults, in your area or elsewhere in your country, which works very well for autistic adults? | Yes | Yes |  |
|  |  |  | No | No | No |
|  |  |  | Don't know | Don't know | Don't know |
| Adult education services | | | | | |
| Are you in an adult education service now or have been at some time in the last 2 years? | Is the adult in an adult education service now or has been at some time in the last 2 years? | Do you have knowledge of and work experience in adult education services that are currently available for adults, including autistic adults? | Yes | Yes | Yes |
|  |  |  | No | No | No |
|  |  |  | N/A | Don’t know | N/A |
| Have you tried to get an adult education service at some time in the last 2 years? | Has the adult, or someone for the adult, tried to get an adult education service at some time in the last 2 years? | N/A | Yes, I have tried to get the service, and I did NOT succeed | Yes, the adult, or someone for the adult, tried to get the service, but did NOT succeed | N/A |
|  |  |  | No, I have not tried to get the service | No, nobody has tried to get the service | N/A |
| If yes, what kind of adult education service were you trying to get? (Check all that apply)  If you have tried to get an adult education service more than one time, please think of the most recent time that you tried. | If yes, what kind of education service did the adult, or someone for the adult, try to get? (Check all that apply) If the adult, or someone for the adult, has tried to get an adult education service more than one time, please think of the most recent time it was tried. | For each type of adult education service available for autistic persons, select the availability that best fits what you know. | Day school or college for adults with autism spectrum (18 years or older) | Day school or college for adults with autism spectrum (18 years or older) | Day school or college for adults with autism spectrum (18 years or older) (Currently available; Not available now, but there are plans in motion to make it available; Not available now, but it is available in other areas of the country; Not available now and, to my knowledge, there are no plans to make it available; Don't know) |
|  |  |  | Boarding school or college for adults with autism spectrum (18 years or older) | Boarding school or college for adults with autism spectrum (18 years or older) | Boarding school or college for adults with autism spectrum (18 years or older) (Currently available; Not available now, but there are plans in motion to make it available; Not available now, but it is available in other areas of the country; Not available now and, to my knowledge, there are no plans to make it available; Don't know) |
|  |  |  | Mentorship or specialist support in regular education settings (high school, technical school, adult education, university) | Mentorship or specialist support in regular education settings (high school, technical school, adult education, university) | Mentorship or specialist support in regular education settings (high school, technical school, adult education, university) (Currently available; Not available now, but there are plans in motion to make it available; Not available now, but it is available in other areas of the country; Not available now and, to my knowledge, there are no plans to make it available; Don't know) |
|  |  |  | Other, please specify | Other, please specify | N/A |
| What kind of office or organization did you go to in order to apply for your adult education service? (Check all that apply) If you have applied for an adult education service more than one time, please think of the most recent time that you applied. | What kind of office or organization was used to apply for the adult’s education service? (Check all that apply) If the adult, or someone for the adult, has applied for an adult education service more than one time, please think of the most recent time that it was applied for. | N/A | Public office or organization | Public office or organization | N/A |
|  |  |  | Private office or organization | Private office or organization | N/A |
|  |  |  | Charity-, advocacy- or volunteer-based organization | Charity-, advocacy- or volunteer-based organization | N/A |
|  |  |  | Don't know | Don't know | N/A |
| At the organization, did the employees that worked with you to apply for adult education services: | At the organization, did the employees that worked with the adult to apply for adult education services: | N/A | Seem knowledgeable about autism spectrum? (Yes; Some were and some were not; No; Don’t know) | Seem knowledgeable about autism spectrum? (Yes; Some were and some were not; No; Don’t know) | N/A |
|  |  |  | Seem knowledgeable about the kinds of services and care that were available for you? (Yes; Some were and some were not; No; Don’t know) | Seem knowledgeable about the kinds of services and care that were available for adults with autism spectrum? (Yes; Some were and some were not; No; Don’t know) | N/A |
|  |  |  | Provide information about care and services that was easy for you to understand? (Yes; Some were and some were not; No; Don’t know) | Provide information about care and services that was easy to understand by the autistic adults? (Yes; Some were and some were not; No; Don’t know) | N/A |
| What kind of adult education service did you get? (Check all that apply) | What kind of adult education service did the adult get? (Check all that apply) | N/A | N/A | N/A | N/A |
|  |  |  | Day school or college for adults with autism spectrum (18 years or older) | Day school or college for adults with autism spectrum (18 years or older) | N/A |
|  |  |  | Boarding school or college for adults with autism spectrum (18 years or older) | Boarding school or college for adults with autism spectrum (18 years or older) | N/A |
|  |  |  | Mentorship or specialist support in regular education settings (high school, technical school, adult education, university) | Mentorship or specialist support in regular education settings (high school, technical school, adult education, university) | N/A |
|  |  |  | Other, please specify | Other, please specify | N/A |
| Was the adult education service set-up for: (Check all that apply) | Was the adult education service set-up for: (Check all that apply) | N/A | All adults | All adults | N/A |
|  |  |  | Adults with handicaps or special needs, specifically | Adults with handicaps or special needs, specifically | N/A |
|  |  |  | Adults with autism spectrum, specifically | Adults with autism spectrum, specifically | N/A |
|  |  |  | Don’t know | Don’t know | N/A |
|  |  |  | Other, please specify | Other, please specify | N/A |
| What was the waiting time before you could get into the adult education service? | What was the waiting time to get into the adult education service that the adult is in right now? | N/A | < 1 month | < 1 month | N/A |
|  |  |  | 1-3 months | 1-3 months | N/A |
|  |  |  | 3-6 months | 3-6 months | N/A |
|  |  |  | > 6 months | > 6 months | N/A |
|  |  |  | Don’t know | Don’t know | N/A |
| N/A | N/A | To the best of your knowledge, what is the waiting time before an autistic adult can get into an education service in the "area where you work now"? | N/A | N/A | Day school or college for adults with autism spectrum (18 years or older) |
|  |  |  | N/A | N/A | Boarding school or college for adults with autism spectrum (18 years or older) |
|  |  |  | N/A | N/A | Mentorship or specialist support in regular |
|  |  |  | N/A | N/A | education settings (high school, technical school, adult education, university) |
| If you could choose an adult education service that fits your needs best now, what would you choose? (Please, tick 1 box) | If you could choose an adult education service that fits the adult’s needs best now, what would you choose? (Please, tick 1 box) | Which 2 types of education services do you think are most needed for autistic adults in the "area where you work now": | I am satisfied with what I have now | The adult is satisfied with what he or she has | I prefer not to make a choice |
|  |  |  | Day school or college for adults with autism spectrum (18 years or older) | Day school or college for adults with autism spectrum (18 years or older) | Day school or college for adults with autism spectrum (18 years or older |
|  |  |  | Boarding school or college for adults with autism spectrum (18 years or older) | Boarding school or college for adults with autism spectrum (18 years or older) | Boarding school or college for adults with autism spectrum (18 years or older) |
|  |  |  | Mentorship or specialist support in regular education settings (high school, technical school, adult education, university) | Mentorship or specialist support in regular education settings (high school, technical school, adult education, university) | Mentorship or specialist support in regular education settings (high school, technical school, adult education, university) |
|  |  |  | Other, please specify | Other, please specify | Other, please specify |
| Do you know of an adult education service, in your area or elsewhere in your country, that works very well for autistic adults? | Do you know of an adult education service, in your area or elsewhere in your country, which works very well for autistic adults? | Do you know of an adult education service, in your area or elsewhere in your country, which works very well for autistic adults? | Yes | Yes | Yes |
|  |  |  | No | No | No |
|  |  |  | Don’t know | Don’t know | Don’t know |
| Financial services | | | | | |
| Are you in a financial service now or have been at some time in the last 2 years? | Is the adult in a financial service now or has been at some time in the last 2 years? | Do you have knowledge of and work experience in financial services that are currently available for adults, including adults with autism spectrum? | Yes | Yes | Yes |
|  |  |  | No | No | No |
|  |  |  | N/A | Don’t know | N/A |
| Have you tried to get a financial service at some time in the last 2 years? | Has the adult, or someone for the adult, tried to get a financial service at some time in the last 2 years? | N/A | Yes, I have tried to get the service, and I did NOT succeed | Yes, the adult, or someone for the adult, tried to get the service, but did NOT succeed | N/A |
|  |  |  | No, I have not tried to get the service | No, nobody has tried to get the service | N/A |
| If yes, what kind of financial service were you trying to get? (Check all that apply) If you have tried to get a financial service more than one time, please think of the most recent time that you tried. | If yes, what kind of financial service did the adult, or someone for the adult, try to get? (Check all that apply) If the adult, or someone for the adult, has tried to get a financial service more than one time, please think of the most recent time it was tried. | For each type of adult financial service available for autistic persons, select the availability that best fits what you know. | Stipend during school or job training | Stipend during school or job training | Stipend/support during school or job training (Currently available; Not available now, but there are plans in motion to make it available; Not available now, but it is available in other areas of the country; Not available now and, to my knowledge, there are no plans to make it available; Don't know) |
|  |  |  | Unemployment benefits (benefit during periods of unemployment) | Unemployment benefits (benefit during periods of unemployment) | Unemployment benefits (benefit during periods of unemployment) (Currently available; Not available now, but there are plans in motion to make it available; Not available now, but it is available in other areas of the country; Not available now and, to my knowledge, there are no plans to make it available; Don't know) |
|  |  |  | Supplementary income for persons unable to have full employment | Supplementary income for persons unable to have full employment | Supplementary income for persons unable to have full employment (Currently available; Not available now, but there are plans in motion to make it available; Not available now, but it is available in other areas of the country; Not available now and, to my knowledge, there are no plans to make it available; Don't know) |
|  |  |  | Full pension (unable to work) | Full pension (unable to work) | Full pension (unable to work) (Currently available; Not available now, but there are plans in motion to make it available; Not available now, but it is available in other areas of the country; Not available now and, to my knowledge, there are no plans to make it available; Don't know) |
|  |  |  | Special 'insurance' to help pay for health care | Special 'insurance' to help pay for health care | Special 'insurance' to help pay for health care (Currently available; Not available now, but there are plans in motion to make it available; Not available now, but it is available in other areas of the country; Not available now and, to my knowledge, there are no plans to make it available; Don't know) |
|  |  |  | Transportation benefits | Transportation benefits | Transportation benefits (Currently available; Not available now, but there are plans in motion to make it available; Not available now, but it is available in other areas of the country; Not available now and, to my knowledge, there are no plans to make it available; Don't know) |
|  |  |  | Caregiver supplementary income (if caregiver duties prevent full employment) | Caregiver supplementary income (if caregiver duties prevent full employment) | Caregiver supplementary income (if caregiver duties prevent full employment) (Currently available; Not available now, but there are plans in motion to make it available; Not available now, but it is available in other areas of the country; Not available now and, to my knowledge, there are no plans to make it available; Don't know) |
|  |  |  | Supported employment (employer receives benefits for providing employment for adults with autism) | Supported employment (employer receives benefits for providing employment for adults with autism) | Supported employment (employer receives benefits for providing employment for adults with autism) (Currently available; Not available now, but there are plans in motion to make it available; Not available now, but it is available in other areas of the country; Not available now and, to my knowledge, there are no plans to make it available; Don't know) |
|  |  |  | Other, please specify | Other, please specify | N/A |
| What kind of office or organization did you go to in order to apply for your financial service? (Check all that apply) If you have applied for a financial service more than one time, please think of the most recent time that you applied. | What kind of office or organization was used to apply for the adult’s financial service? (Check all that apply) If the adult, or someone for the adult, has applied for a financial service more than one time, please think of the most recent time that it was applied for. | N/A | Public office or organization | Public office or organization | N/A |
|  |  |  | Private office or organization | Private office or organization | N/A |
|  |  |  | Charity-, advocacy- or volunteer-based organization | Charity-, advocacy- or volunteer-based organization | N/A |
|  |  |  | Don't know | Don't know | N/A |
| At the organization, did the employees that worked you to apply for financial services: | At the organization, did the employees that worked with the adult to apply for financial services: | N/A | Seem knowledgeable about autism spectrum? (Yes; Some were and some were not; No; Don’t know) | Seem knowledgeable about autism spectrum? (Yes; Some were and some were not; No; Don’t know) | N/A |
|  |  |  | Seem knowledgeable about the kinds of services and care that were available to you? (Yes; Some were and some were not; No; Don’t know) | Seem knowledgeable about the kinds of services and care that were available for adults with autism? (Yes; Some were and some were not; No; Don’t know) | N/A |
|  |  |  | Provide information about care and services that was easy for you to understand? (Yes; Some were and some were not; No; Don’t know) | Provide information about care and services that was easy to understand by the autistic adult? (Yes; Some were and some were not; No; Don’t know) | N/A |
| What kind of financial service did you get? (Check all that apply) | What kind of financial service did the adult get? (Check all that apply) | N/A | Stipend during school or job training | Stipend during school or job training | N/A |
|  |  |  | Unemployment benefits (benefit during periods of unemployment) | Unemployment benefits (benefit during periods of unemployment) | N/A |
|  |  |  | Supplementary income for persons unable to have full employment | Supplementary income for persons unable to have full employment | N/A |
|  |  |  | Full pension (unable to work) | Full pension (unable to work) | N/A |
|  |  |  | Special 'insurance' to help pay for health care | Special 'insurance' to help pay for health care | N/A |
|  |  |  | Transportation benefits | Transportation benefits | N/A |
|  |  |  | Caregiver supplementary income (if caregiver duties prevent full employment) | Caregiver supplementary income (if caregiver duties prevent full employment) | N/A |
|  |  |  | Supported employment (employer receives benefits for providing employment for adults with autism) | Supported employment (employer receives benefits for providing employment for adults with autism) | N/A |
|  |  |  | Other, please specify | Other, please specify | N/A |
| Was the financial service set-up for: (Check all that apply) | Was the financial service set-up for: (Check all that apply) | N/A | All adults | All adults | N/A |
|  |  |  | Adults with handicaps or special needs, specifically | Adults with handicaps or special needs, specifically | N/A |
|  |  |  | Adults with autism spectrum, specifically | Adults with autism spectrum, specifically | N/A |
|  |  |  | Don’t know | Don’t know | N/A |
|  |  |  | Other, please specify | Other, please specify | N/A |
| What was the waiting time before you could get into the financial service? | What was the waiting time for the adult to get into the financial service? | N/A | < 1 month | < 1 month | N/A |
|  |  |  | 1-3 months | 1-3 months | N/A |
|  |  |  | 3-6 months | 3-6 months | N/A |
|  |  |  | > 6 months | > 6 months | N/A |
|  |  |  | Don’t know | Don’t know | N/A |
| N/A | N/A | To the best of your knowledge, what is the waiting time before an autistic person can receive a financial service in the "area where you work now"? | N/A | N/A | Stipend during school or job training (This service is not currently available; < 1 month; 1-3 months; 3-6 months; > 6 month; Don't know) |
|  |  |  | N/A | N/A | Unemployment benefits (benefit during periods of unemployment) (This service is not currently available; < 1 month; 1-3 months; 3-6 months; > 6 month; Don't know) |
|  |  |  | N/A | N/A | Supplementary income (This service is not currently available; < 1 month; 1-3 months; 3-6 months; > 6 month; Don't know) for persons unable to have full employment (This service is not currently available; < 1 month; 1-3 months; 3-6 months; > 6 month; Don't know) |
|  |  |  | N/A | N/A | Full pension (unable to work) (This service is not currently available; < 1 month; 1-3 months; 3-6 months; > 6 month; Don't know) |
|  |  |  | N/A | N/A | Special 'insurance' to help pay for health care (This service is not currently available; < 1 month; 1-3 months; 3-6 months; > 6 month; Don't know) |
|  |  |  | N/A | N/A | Transportation benefits (This service is not currently available; < 1 month; 1-3 months; 3-6 months; > 6 month; Don't know) |
|  |  |  | N/A | N/A | Caregiver supplementary income (if caregiver duties prevent full employment) (This service is not currently available; < 1 month; 1-3 months; 3-6 months; > 6 month; Don't know) |
|  |  |  | N/A | N/A | Supported employment (employer receives benefits for providing employment for adults with autism) (This service is not currently available; < 1 month; 1-3 months; 3-6 months; > 6 month; Don't know) |
| If you could choose a financial service that fits your needs best now, what would you choose? (Please, tick 1 box) | If you could choose a financial service that fits the adult’s needs best now, what would you choose? (Please, tick 1 box) | Which 2 types of financial services do you think are most needed for autistic adults in the "area where you work now"? (Please, tick 2 boxes) | I am satisfied with what I have now | The adult is satisfied with what they have | I prefer not to make a choice |
|  |  |  | Stipend during school or job training | Stipend during school or job training | Stipend during school or job training |
|  |  |  | Unemployment benefits (benefit during periods of unemployment) | Unemployment benefits (benefit during periods of unemployment) | Unemployment benefits (benefit during periods of unemployment) |
|  |  |  | Supplementary income for persons unable to have full employment | Supplementary income for persons unable to have full employment | Supplementary income for persons unable to have full employment |
|  |  |  | Full pension (unable to work) | Full pension (unable to work) | Full pension (unable to work) |
|  |  |  | Special 'insurance' to help pay for health care | Special 'insurance' to help pay for health care | Special 'insurance' to help pay for health care |
|  |  |  | Transportation benefits | Transportation benefits | Transportation benefits |
|  |  |  | Caregiver supplementary income (if caregiver duties prevent full employment) | Caregiver supplementary income (if caregiver duties prevent full employment) | Caregiver supplementary income (if caregiver duties prevent full employment) |
|  |  |  | Supported employment (employer receives benefits for providing employment for adults with autism) | Supported employment (employer receives benefits for providing employment for adults with autism) | Supported employment (employer receives benefits for providing employment for adults with autism) |
|  |  |  | Other, please specify | Other, please specify | Other, please specify |
| Do you know of a financial service, in your area or elsewhere in your country, that works very well for autistic adults? | Do you know of a financial service, in your area or elsewhere in your country, which works very well for autistic adults? | N/A | Yes | Yes | N/A |
|  |  |  | No | No | N/A |
|  |  |  | Don’t know | Don’t know | N/A |
| Social support services | | | | | |
| Are you in a social support service now or have been at some time in the last 2 years? | Is the adult in a social support service now or has been at some time in the last 2 years? | Do you have knowledge of and work experience in social support services that are currently available for adults, including adults on the autism spectrum? | Yes | Yes | Yes |
|  |  |  | No | No | No |
|  |  |  | N/A | Don’t know | N/A |
| Have you tried to get a social support service at some time in the last 2 years? | Has the adult, or someone for the adult, you tried to get a social support service at some time in the last 2 years? | N/A | Yes, I have tried to get the service, and I did NOT succeed | Yes, the adult, or someone for the adult, tried to get the service, but did NOT succeed | N/A |
|  |  |  | No, I have not tried to get the service | No, nobody has tried to get the | N/A |
| If yes, what kind of social support service were you trying to get? (Check all that apply) If you have tried to get a social support service more than one time, please think of the most recent time that you tried. | If yes, what kind of social support service did the adult, or someone for the adult, try to get? (Check all that apply) If the adult, or someone for the adult, has tried to get a social support service more than one time, please think of the most recent time it was tried. | For each type of adult social support services available for autistic persons, select the availability that best fits what you know. | Life skills training | Life skills training | Life skills training (Currently available; Not available now, but there are plans in motion to make it available; Not available now, but it is available in other areas of the country; Not available now and, to my knowledge, there are no plans to make it available; Don't know) |
|  |  |  | Support groups, on-line | Support groups, on-line | Support groups, on-line (Currently available; Not available now, but there are plans in motion to make it available; Not available now, but it is available in other areas of the country; Not available now and, to my knowledge, there are no plans to make it available; Don't know) |
|  |  |  | Support groups, in person | Support groups, in person | Support groups, in person (Currently available; Not available now, but there are plans in motion to make it available; Not available now, but it is available in other areas of the country; Not available now and, to my knowledge, there are no plans to make it available; Don't know) |
|  |  |  | Free time activities | Free time activities | Free time activities (Currently available; Not available now, but there are plans in motion to make it available; Not available now, but it is available in other areas of the country; Not available now and, to my knowledge, there are no plans to make it available; Don't know) |
|  |  |  | Family relations support and counseling | Family relations support and counseling | Family relations support and counseling (Currently available; Not available now, but there are plans in motion to make it available; Not available now, but it is available in other areas of the country; Not available now and, to my knowledge, there are no plans to make it available; Don't know) |
|  |  |  | Behavior training, for an individual | Behavior training, for an individual | Behavior training, for an individual (Currently available; Not available now, but there are plans in motion to make it available; Not available now, but it is available in other areas of the country; Not available now and, to my knowledge, there are no plans to make it available; Don't know) |
|  |  |  | Behavior training, in groups | Behavior training, in groups | Behavior training, in groups (Currently available; Not available now, but there are plans in motion to make it available; Not available now, but it is available in other areas of the country; Not available now and, to my knowledge, there are no plans to make it available; Don't know) |
|  |  |  | Program for matching autistic peers with each other (persons similar to each other, peer-to-peer) | Program for matching autistic peers with each other (persons similar to each other, peer-to-peer) | Program for matching autistic peers with each other (persons similar to each other, peer-to-peer) (Currently available; Not available now, but there are plans in motion to make it available; Not available now, but it is available in other areas of the country; Not available now and, to my knowledge, there are no plans to make it available; Don't know) |
|  |  |  | Program for matching an autistic person with a non-autistic peer (someone similar to the autistic person in age, gender, interests, etc.) | Program for matching and autistic person with a non-autistic peer (someone similar to the autistic person in age, gender, interests, etc.) | Program for matching and autistic person with a non-autistic peer (someone similar to the autistic person in age, gender, interests, etc.) (Currently available; Not available now, but there are plans in motion to make it available; Not available now, but it is available in other areas of the country; Not available now and, to my knowledge, there are no plans to make it available; Don't know) |
|  |  |  | Other, please specify | Other, please specify | N/A |
| What kind of office or organization did you go to in order to apply for your social support service? (Check all that apply) If you have applied for a social support service more than one time, please think of the most recent time that you applied. | What kind of office or organization was used to apply for the adult’s social support service? (Check all that apply) If the adult, or someone for the adult, has applied for a social support service more than one time, please think of the most recent time that it was applied for. | N/A | Public office or organization | Public office or organization | N/A |
|  |  |  | Private office or organization | Private office or organization | N/A |
|  |  |  | Charity-, advocacy- or volunteer-based organization | Charity-, advocacy- or volunteer-based organization | N/A |
|  |  |  | Don't know | Don't know | N/A |
| At the organization, did the employees that worked with you to apply for social support services: | At the organization, did the employees that worked with the adult to apply for social support services: | N/A | Seem knowledgeable about autism spectrum? (Yes; Some were and some were not; No; Don’t know) | Seem knowledgeable about autism spectrum? (Yes; Some were and some were not; No; Don’t know) | N/A |
|  |  |  | Seem knowledgeable about the kinds of services and care that were available to you? (Yes; Some were and some were not; No; Don’t know) | Seem knowledgeable about the kinds of services and care that were available for adults with autism? (Yes; Some were and some were not; No; Don’t know) | N/A |
|  |  |  | Provide information about care and services that was easy for you to understand? (Yes; Some were and some were not; No; Don’t know) | Provide information about care and services that was easy to understand by the autistic adult? (Yes; Some were and some were not; No; Don’t know) | N/A |
| What kind of social support service did you get? (Check all that apply) | What kind of social support service did the adult get? (Check all that apply) | N/A | Life skills training | Life skills training | N/A |
|  |  |  | Support groups, on-line | Support groups, on-line | N/A |
|  |  |  | Support groups, in person | Support groups, in person | N/A |
|  |  |  | Free time activities | Free time activities | N/A |
|  |  |  | Family relations support and counseling | Family relations support and counseling | N/A |
|  |  |  | Behavior training, for an individual | Behavior training, for an individual | N/A |
|  |  |  | Behavior training, in groups | Behavior training, in groups | N/A |
|  |  |  | Program for matching autistic peers with each other (persons similar to each other, peer-to-peer) | Program for matching autistic peers with each other (persons similar to each other, peer-to-peer) | N/A |
|  |  |  | Program for matching an autistic person with a non-autistic peer (someone similar to the autistic person in age, gender, interests, etc.) | Program for matching an autistic person with a non-autistic peer (someone similar to the autistic person in age, gender, interests, etc.) | N/A |
|  |  |  | Other, please specify | Other, please specify | N/A |
| Was the social support service set-up for: (Check all that apply) | Was the social support service set-up for: (Check all that apply) | N/A | All adults | All adults | N/A |
|  |  |  | Adults with handicaps or special needs, specifically | Adults with handicaps or special needs, specifically | N/A |
|  |  |  | Adults with autism spectrum, specifically | Adults with autism spectrum, specifically | N/A |
|  |  |  | Don’t know | Don’t know | N/A |
|  |  |  | Other, please specify | Other, please specify | N/A |
| What was the waiting time before you could get into the social support service? | What was the waiting time for the adult to get into the social service? | N/A | < 1 month | < 1 month | N/A |
|  |  |  | 1-3 months | 1-3 months | N/A |
|  |  |  | 3-6 months | 3-6 months | N/A |
|  |  |  | > 6 months | > 6 months | N/A |
|  |  |  | Don't know | Don't know | N/A |
| N/A | N/A | To the best of your knowledge, what is the waiting time before an autistic person can get into a social support service in the "area where you work now"? | N/A | N/A | Free time activities (This service not currently available; < 1 months; 1-3 months; 3-6 months; > 6 months; Don’t know) |
|  |  |  | N/A | N/A | Family relations support and counseling (This service not currently available; < 1 months; 1-3 months; 3-6 months; > 6 months; Don’t know) |
|  |  |  | N/A | N/A | Behavior training, for an individual (This service not currently available; < 1 months; 1-3 months; 3-6 months; > 6 months; Don’t know) |
|  |  |  | N/A | N/A | Behavior training, in groups (This service not currently available; < 1 months; 1-3 months; 3-6 months; > 6 months; Don’t know) |
|  |  |  | N/A | N/A | Program for matching autistic peers with each other (persons similar to each other, peer-to-peer) (This service not currently available; < 1 months; 1-3 months; 3-6 months; > 6 months; Don’t know) |
|  |  |  | N/A | N/A | Program for matching an autistic person with a non-autistic peer (someone similar to the autistic person in age, gender, interests, etc.) (This service not currently available; < 1 months; 1-3 months; 3-6 months; > 6 months; Don’t know) |
| If you could choose a social support service that fits your needs best now, what would you choose? (Please, tick 1 box) | If you could choose a social support service that fits the adult’s needs best now what would you choose? (Please, tick 1 box) | Which 2 types of social support services for autistic adults do you think are most needed in "the area where you work now": (Please, tick 2 boxes). | I am satisfied with what I have now | The adult is satisfied with what they have | I prefer not to make a choice |
|  |  |  | Life skills training | Life skills training | Life skills training |
|  |  |  | Support groups, on-line | Support groups, on-line | Support groups, on-line |
|  |  |  | Support groups, in person | Support groups, in person | Support groups, in person |
|  |  |  | Free time activities | Free time activities | Free time activities |
|  |  |  | Family relations support and counseling | Family relations support and counseling | Family relations support and counseling |
|  |  |  | Behavior training, for an individual | Behavior training, for an individual | Behavior training, for an individual |
|  |  |  | Behavior training, in groups | Behavior training, in groups | Behavior training, in groups |
|  |  |  | Program for matching autistic peers with each other (persons similar to each other, peer-to-peer) | Program for matching autistic peers with each other (persons similar to each other, peer-to-peer) | Program for matching autistic peers with each other (persons similar to each other, peer-to-peer) |
|  |  |  | Program for matching an autistic person with a non-autistic peer (someone similar to the autistic person in age, gender, interests, etc.) | Program for matching an autistic person with a non-autistic peer (someone similar to the autistic person in age, gender, interests, etc.) | Program for matching an autistic person with a non-autistic peer (someone similar to the autistic person in age, gender, interests, etc.) |
|  |  |  | Other, please specify | Other, please specify | Other, please specify |
| Do you know of a social support service, in your area or elsewhere in your country, that works very well for autistic adults? | Do you know of a social support service, in your area or elsewhere in your country, which works very well for autistic adults? | Do you know of a social support service for adults, in your area or elsewhere in your country, which works very well for autistic adults? | Yes | Yes | Yes |
|  |  |  | No | No | No |
|  |  |  | Don’t know | Don’t know | Don’t know |

*Note*. N/A = Question not available for the correspondent group.

**Supplementary Data 2**

Demographic background information of the respondents: adults, carers, and professionals

| Characteristic | Answer | Autistic adult  (N=469) |  | Carer / Cared-for adult  (N=441) |  | Professional  (N=175) |
| --- | --- | --- | --- | --- | --- | --- |
| Gender | Female | 308 (65.7) |  | 361 (81.9) / 121(27.4) |  | 133 (76.0) |
|  | Male | 144 (30.7) |  | 79 (17.9) / 319 (72.3) |  | 42 (24.0) |
|  | Other or no answer | 17 (3.6) |  | 1 (0.2) / 1 (0.2) |  | 0 (0.0) |
| Age (years) | 18-25 | 99 (21.1) |  | 7 (1.6) / 239 (54.2) |  | N/A |
|  | 26-35 | 162 (34.5) |  | 22 (5.0) / 122 (27.7) |  |  |
|  | 36-45 | 108 (23.0) |  | 54 (12.2) / 53 (12.0) |  |  |
|  | 46-55 | 82 (17.5) |  | 177 (40.1) / 23 (5.2) |  |  |
|  | 56-64 | 15 (3.2) |  | 117 (26.5) / 3 (0.7) |  |  |
|  | > 65 | 3 (0.6) |  | 64 (14.5) / 1 (0.2) |  |  |
| Living area | | | | | | |
| Country | Denmark | 193 (41.2) |  | 122 (27.7) |  | 95 (54.3) |
|  | France | 61 (13.0) |  | 67 (15.2) |  | 19 (10.9) |
|  | Spain | 22 (4.7) |  | 86 (19.5) |  | 17 (9.7) |
|  | Finland | 63 (13.4) |  | 36 (8.2) |  | 9 (5.1) |
|  | Poland | 38 (8.1) |  | 48 (10.9) |  | 2 (1.1) |
|  | Italy | 36 (7.7) |  | 32 (7.3) |  | 11 (6.3) |
|  | Iceland | 16 (3.4) |  | 40 (9.7) |  | 15 (8.6) |
|  | Republic of Ireland | 14 (3.0) |  | 10 (2.3) |  | 0 |
|  | United Kingdom | 20 (4.3) |  | 0 |  | 2 (1.1) |
|  | Germanyy | 6 (1.3) |  | 0 |  | 0 |
|  | Portugal | 0 |  | 0 |  | 2 (1.1) |
| Living area | Capital city | 115 (24.5) |  | 160 (36.3) |  | 52 (29.7) |
|  | Other than a capital city | 354 (75.5) |  | 281 (63.7) |  | 123 (70.3) |
| Community size | < 1.000 people | 28 (6.0) |  | 31 (7.0) |  | 6 (3.4) |
|  | 1.000-20.000 people | 93 (19.8) |  | 96 (21.8) |  | 30 (17.1) |
|  | 20.000-100.000 people | 134 (28.6) |  | 116 (26.3) |  | 82 (46.9) |
|  | 100.000-1.000.000 | 125 (26.7) |  | 111 (25.2) |  | 45 (25.7) |
|  | > 1.000.000 | 46 (9.8) |  | 60 (13.6) |  | 12 (6.9) |
|  | Don't Know | 43 (9.2) |  | 27 (6.1) |  | 0 |
| Education | | | | | | |
| Adult is attending school, home school or education program now | Yes, full time | 56 (11.9) |  | N/A |  | N/A |
|  | Yes, part time | 49 (10.5) |  |  |  |  |
|  | No | 364 (77.6) |  |  |  |  |
| If yes, actual education level | Primary level school | 2 (1.9) |  | N/A |  | N/A |
|  | Secondary level school | 22 (21.0) |  |  |  |  |
|  | Technical, vocational or job training school | 16 (15.2) |  |  |  |  |
|  | College or University | 65 (61.9) |  |  |  |  |
| If no, completed education level | Primary level school | 39 (10.7) |  | N/A |  | N/A |
|  | Secondary level school | 67 (18.4) |  |  |  |  |
|  | Technical, vocational or job training school | 91 (25.0) |  |  |  |  |
|  | College or University | 149 (40.9) |  |  |  |  |
|  | Don't Know | 18 (5.0) |  |  |  |  |
| Adult remember the age when completing education | Yes | 332 (91.2) |  | N/A |  | N/A |
|  | No | 32 (8.8) |  |  |  |  |
| Adult age at completed education | Mean + Standard deviation [range] | 24.4 ± 7.9 [3-52] |  | N/A |  | N/A |
| Carer years of education | < 10 years | N/A |  | 43 (9.8) |  | N/A |
|  | 10-12 years |  |  | 33 (7.5) |  |  |
|  | 13-16 years |  |  | 109 (24.7) |  |  |
|  | >16 years |  |  | 220 (49.9) |  |  |
|  | Other |  |  | 36 (8.2) |  |  |
| Employment | | | | | | |
| Current employment status | Unemployed | 247 (52.7) |  | 46 (10.4) |  | N/A |
|  | Employed (part time o full time) | 156 (33.3) |  | 232 (52.6) |  |  |
|  | Self-employed | 25 (5.3) |  | 44 (10.0) |  |  |
|  | Student | N/A |  | 9 (2.0) |  |  |
|  | Volunteer | 41 (8.7) |  | 17 (3.9) |  |  |
|  | Retired | N/A |  | 93 (21.1) |  |  |
| If unemployed, reasons for unemployment | A disability that prevents from having a job | 80 (32.4) |  | N/A |  | N/A |
|  | Student | 35 (14.2) |  |  |  |  |
|  | Looking for a job | 35 (14.2) |  |  |  |  |
|  | Believing that she/he cannot find a job | 20 (8.1) |  |  |  |  |
|  | Retired | 19 (7.7) |  |  |  |  |
|  | Other | 58 (23.5) |  |  |  |  |
| Age at diagnosis | | | | | | |
| Age at autism diagnosis | 16-25 | 63 (20.3) |  | 42 (58.3) |  | N/A |
|  | 26-35 | 114 (36.7) |  | 14 (19.4) |  |  |
|  | 36-45 | 78 (25.1) |  | 10 (13.9) |  |  |
|  | 46-55 | 48 (15.4) |  | 5 (6.9) |  |  |
|  | >=56 | 8 (2.5) |  | 1 (1.4) |  |  |
| Carers’ relationship with the adult | | | | | | |
| Years of knowledge of the adult | Less than 1 year | N/A |  | 5 (1.1) |  | N/A |
|  | 1-5 years |  |  | 11 (2.5) |  |  |
|  | 5-10 years |  |  | 8 (1.8) |  |  |
|  | More than 10 years but not the adult's whole life |  |  | 29 (6.6) |  |  |
|  | The adult's whole life |  |  | 388 (88.0) |  |  |
| Relationship with the adult | Parent | N/A |  | 370 (83.9) |  | N/A |
|  | Other family member related by blood |  |  | 33 (7.5) |  |  |
|  | Spouse or partner |  |  | 15 (3.4) |  |  |
|  | A carer, but not a family member, spouse, or partner |  |  | 23 (5.2) |  |  |
| Level of independence of the autistic adult | High level of independence | N/A |  | 42 (9.5) |  | N/A |
|  | Some independence but needs support |  |  | 180 (40.8) |  |  |
|  | Needs a high level of support in daily living |  |  | 149 (33.8) |  |  |
|  | Needs high level institution-like care |  |  | 70 (15.9) |  |  |
| Professionals’ backgrounds and characteristics of their workplace | | | | | | |
| Professional type | Other | N/A |  | N/A |  | 55 (31.4) |
|  | Teacher/pedagogue |  |  |  |  | 45 (25.7) |
|  | Social worker |  |  |  |  | 44 (25.1) |
|  | Psychologist |  |  |  |  | 20 (11.4) |
|  | Physical or occupational therapist |  |  |  |  | 7 (4.0) |
|  | Psychiatrist |  |  |  |  | 1 (0.6) |
|  | Teaching assistant/nursery assistant |  |  |  |  | 2 (1.1) |
|  | Nurse |  |  |  |  | 1 (0.6) |
|  | General practitioner |  |  |  |  | 0 (0.0) |
|  | Medical specialist, other than psychiatrist |  |  |  |  | 0 (0.0) |
|  | Other medical professional (open answer) |  |  |  |  | 0 (0.0) |
|  | Mental health therapist |  |  |  |  | 0 (0.0) |
|  | Criminal justice (e.g., police, courts, legal advocate) |  |  |  |  | 0 (0.0) |
| Years in jobs in adult services and care | <1 year | N/A |  | N/A |  | 4 (2.3) |
|  | 1-2 years |  |  |  |  | 9 (5.1) |
|  | 3-5 years |  |  |  |  | 24 (13.7) |
|  | 6-10 years |  |  |  |  | 36 (20.6) |
|  | >10 years |  |  |  |  | 102 (58.3) |
| Source of experience and knowledge about services for adults | Current job location (e.g., capital city or small town) | N/A |  | N/A |  | 74 (42.3) |
|  | Current job location and a wider area (e.g., the region or state where your job is located) |  |  |  |  | 84 (48.0) |
|  | Most closely connected to the whole country |  |  |  |  | 17 (9.7) |

*Note*. N/A = Question not available for the correspondent group. Values expressed as number of responders and frequencies (in parenthesis).

**SupplementaryData3a**

*Type of organization where applied for a given service*

| Autistic adult (N=646) | | | | | |
| --- | --- | --- | --- | --- | --- |
|  | Residential service  (n=213) | Employment service  (n=197) | Adult education service  (n=105) | Financial service  (n=259) | Social support service  (n=192) |
| Public office or organization | 145 (68.10) | 132 (67.00) | 63 (60.00) | 234 (90.35) | 118 (61.46) |
| Private office or organization | 31 (14.55) | 30 (15.23) | 20 (19.05) | 7 (2.70) | 25 (13.02) |
| Charity-, advocacy- or volunteer-based organization | 13 (6.10) | 19 (9.64) | 8 (7.62) | 13 (5.02) | 38 (19.79) |
| Don't know | 24 (11.27) | 16 (8.12) | 14 (13.33) | 5 (1.93) | 11 (5.73) |
| Carer (N=575) | | | | | |
|  | Residential service  (n=299) | Employment service  (n=181) | Adult education service  (n=181) | Financial service  (n=309) | Social support service  (n=248) |
| Public office or organization | 199 (66.56) | 118 (65.19) | 108 (59.69) | 290 (93.85) | 153 (61.69) |
| Private office or organization | 66 (22.07) | 35 (19.34) | 46 (25.41) | 6 (1.94) | 54 (21.77) |
| Charity-, advocacy- or volunteer-based organization | 20 (6.69) | 15 (8.29) | 13 (7.18) | 6 (1.94) | 36 (14.52) |
| Don't know | 14 (4.68) | 13 (7.18) | 14 (7.73) | 7 (2.27) | 5 (2.02) |

*Note*. Values expressed as number of responders and frequencies (in parenthesis). Kind of service the adult got: data are for adults and carers of adults who were in a residential/employment/adult education/financial/social support service now or in the last 2 years. The questions for the autistic adults (or carers) were: “*What kind of office or organization did you go to in order to apply foryour* (carers: *was used to apply for the adult’s) <residential/employment/adult education/financial/social support> service? (Check all that apply). If you have* (carers: *If the adult, or someone for the adult, has) applied for a <residential/employment/adult education/financial/social support> service more than one time, please think of the most recent time that you applied* (carer: *that it was applied for).”*

**Supplementary Data 3b**

Client group that a given service was set up for

| Autistic adult (N=646) | | | | | |
| --- | --- | --- | --- | --- | --- |
| Client group | Residential service  (n=162) | Employment service  (n=152) | Adult education service  (n=74) | Financial service  (n=221) | Social support service  (n=161) |
| All adults | 15 (9.26) | 59 (38.82) | 25 (33.78) | 100 (45.24) | 22 (13.66) |
| Adults with handicaps or special needs, specifically | 57 (35.19) | 44 (28.95) | 29 (39.19) | 80 (36.20) | 45 (27.95) |
| Adults with autism spectrum, specifically | 65 (40.12) | 25 (16.45) | 10 (13.51) | 9 (4.07) | 66 (40.99) |
| Don’t know | 11 (6.79) | 12 (7.89) | 5 (6.76) | 15 (6.79) | 12 (7.45) |
| Other, please specify | 14 (8.64) | 12 (7.89) | 5 (6.76) | 17 (7.69) | 16(9.94) |
| Carer (N=575) | | | | | |
| Client group | Residential service  (n=232) | Employment service  (n=135) | Adult education service  (n=138) | Financial service  (n=257) | Social support service  (n=164) |
| All adults | 10 (4.31) | 25 (18.52) | 27 (19.57) | 50 (19.46) | 8 (4.88) |
| Adults with handicaps or special needs, specifically | 71 (30.60) | 51 (37.78) | 54 (39.13) | 185 (71.98) | 60 (36.59) |
| Adults with autism spectrum, specifically | 133 (57.33) | 41 (30.37) | 48 (34.78) | 22 (8.56) | 80 (48.78) |
| Don’t know | 7 (3.02) | 10 (7.41) | 7 (5.07) | 23 (8.95) | 7 (4.27) |
| Other, please specify | 14 (4.74) | 8 (5.93) | 2 (1.45) | 7 (2.72) | 9 (5.49) |

*Note*. Values expressed as number of responders and frequencies (in parenthesis). Kind of service the adult got: data are for adults and carers of adults who were in a residential/employment/adult education/financial/social support service now or in the last 2 years. The questions for the autistic adults and carers were: “*Was the residential/employment/adult education/financial/social service set up for: (Check all that apply).”*

**Supplementary Data 4a**

*Preferred residential services*

|  | Autistic adult (n=469) | Carer (n=441) | | Professional  (n=301) |
| --- | --- | --- | --- | --- |
|  |  | High independence (n=222) | Low independence (n=219) |  |
|  | N (%) | N (%) | N (%) | N (%) |
| Adult satisfied | 190 (40.5) | 70 (31.5) | 51 (23.9) | NA |
| I prefer not to make a choice | NA | NA | NA | 30 (9.9)***** |
| Help in own home | 125 (26.6) | 73 (32.8) | 43 (19.6) | 74 (27.3) |
| Day center | 22 (4.6) | 11 (4.9) | 27 (12.3) | 40 (14.7) |
| Full time residential facility | 18 (3.8) | 12 (5.4) | 71 (32.4) | NA |
| Full time residential facility with private bedroom only | NA | NA | NA | 32 (11.8) |
| Full time residential facility with full apartment | NA | NA | NA | 69 (25.4) |
| Help at college or school dormitory | 10 (2.1) | 22 (9.9) | 4 (1.8) | 14 (5.2) |
| Caregiver respite care | 21 (4.4) | NA | NA | 20 (7.3) |
| Other | 83 (17.7) | 34 (15.3) | 23 (10.5) | 22 (8.1) |

*Note*. NA=Question not available for the correspondent group. Data are for all adults and carers of adults. The question for the autistic adults was: “*If you could choose a residential service that fits your needs best now, what would you choose? (Please, tick 1 box)”.* The question for the carers of autistic adults was: *“If you could choose a residential service that fits the adult’s needs best now, what would you choose? (Please, tick 1 box)”.* The carers’ data are stratified by high and low level of independence of the autistic adult. The question for the professional was: *“Which 2 types of residential services do you think are most needed for autistic adults in the (geographical) area where you work now? (Please, tick 2 boxes).”* Professionals who selected “*I prefer not to make a choice*” are not included in the calculation of % for each of the other answer choices. The %s for the other answer choices are based on the professionals who made a choice, n=271 (301-30=271). *This % is calculated on the total sample of the professionals’ responders (n=301).

**Supplementary Data 4b**

***Residential services availability***

|  | Kind of residential service that was applied for, but failed | | | Kind of residential service the adult got | | | Residential service availability | | | | |
| --- | --- | --- | --- | --- | --- | --- | --- | --- | --- | --- | --- |
|  | Autistic adult (n=48) | Carer High indipendence (n=25) | Carer Low independence (n=30) | Autistic adult (n=138) | Carer  High indipendence (n=71) | Carer  Low independence (n=146) | Professional (n=176) | | | | |
|  |  |  |  |  |  |  | Currently available | Not available now, but there are plans in motion to make it available | Not available now, but it is available in other areas of the country | Not available now and, to my knowledge, there are no plans to make it available | Don't know |
| Help in own home | 28 (41.18) | 12  (34.29) | 11 (23.91) | 91 (53.21) | 35 (38.89) | 23 (11.98) | 139 (78.98) | 7 (3.98) | 4 (2.27) | 9 (5.11) | 17 (9.66) |
| Day  Center | 7 (10.29) | 3 (8.57) | 9 (19.57) | 16 (9.36) | 14 (15.56) | 42 (21.88) | 130 (73.86) | 7 (3.98) | 12 (6.82) | 9 (5.11) | 18 (10.23) |
| Full time residential facility | 6 (8.82) | 5 (14.29) | 12 (26.09) | 14 (8.20) | 12 (13.13) | 84 (43.75) | NA | NA | NA | NA | NA |
| Full time residential facility with private bedroom only | NA | NA | NA | NA | NA | NA | 104 (59.09) | 8 (4.55) | 9 (5.11) | 23 (13.07) | 32 (18.18) |
| Full time residential facility with full apartment | NA | NA | NA | NA | NA | NA | 111 (63.07) | 8 (4.55) | 15 (8.52) | 19 (10.80) | 23 (13.07) |
| Help at college or school dormitory | 4 (5.88) | 6 (17.14) | 1 (2.17) | 2 (1.17) | 2 (2.22) | 3 (1.56) | 74 (42.05) | 6 (3.41) | 9 (5.11) | 35 (19.89) | 52 (29.55) |
| Caregiver respite care | 8 (11.76) | 2 (5.71) | 8 (17.39) | 10 (5.85) | 4 (4.44) | 22 (11.46) | 99 (56.25) | 13 (7.39) | 7 (3.98) | 22 (12.50) | 35 (19.89) |
| Other | 15 (22.06) | 7 (20.00) | 5 (10.87) | 38 (22.22) | 23 (25.56) | 18 (9.38) | NA | NA | NA | NA | NA |

*Note*. Values expressed as number of responders and frequencies (in parenthesis). NA=Question not available for the correspondent group. Kind of residential service that was applied for but failed: data are for adults and carers of adults who were not in a residential service now or in the last 2 years but had tried to get a residential service in the last 2 years and failed. Kind of residential service the adult got: data are for adults and carers of adults who were in a residential service now or in the last 2 years. The question for the autistic adults was: “*What kind of residential service were you trying to get? (Check all that apply).”* The question for the carers of autistic adults was: *“What kind of residential service did the adult, or someone for the adult, try to get? (Check all that apply)”.* The carers’ data are stratified by high and low level of independence of the autistic adult. The question for the professionals was: *“For each type of adult residential service available for autistic persons, select the availability that best fits what you know.”*

**Supplementary Data 4c**

***Residential services availability by gender***

|  | Kind of residential service that was applied for, but failed  (Autistic Adult=48)  (male 18, female 28, other/no answer 2) | | | Kind of residential service the adult got  (Autistic Adult=138)  (male 44, female 92, other/no answer 2) | | |
| --- | --- | --- | --- | --- | --- | --- |
|  | Male | Female | Other/No answer | Male | Female | Other/No answer |
|
| Help in own home | 10 (6.9) | 18  (5.8) | 0 (0.0) | 22 (15.3) | 67 (21.8) | 2 (11.8) |
| Day  Center | 4 (2.8) | 2 (0.7) | 1 (5.9) | 6 (4.2) | 10 (3.3) | 0 (0.0) |
| Full time residential facility | 4 (2.8) | 2 (0.7) | 0 (0.0) | 7 (4.9) | 7 (2.3) | 0 (0.0) |
| Help at college or school dormitory | 2 (1.4) | 1 (0.3) | 1 (5.9) | 2 (1.4) | 0 (0.0) | 0 (0.0) |
| Caregiver respite care | 3 (2.1) | 5 (1.6) | 0 (0.0) | 5 (3.5) | 5 (1.6) | 0 (0.0) |
| Other | 3 (2.1) | 12 (3.9) | 0 (0.0) | 12 (8.3) | 26 (8.4) | 0 (0.0) |

*Note*. Values expressed as number of responders and frequencies (in parenthesis). NA=Question not available for the correspondent group. Kind of residential service that was applied for but failed: data are for adults and carers of adults who were not in a residential service now or in the last 2 years but had tried to get a residential service in the last 2 years and failed. Kind of residential service the adult got: data are for adults and carers of adults who were in a residential service now or in the last 2 years. The question for the autistic adults was: “*What kind of residential service were you trying to get? (Check all that apply).”*

**Supplementary Data 4d**

*Preference of residential services by gender*

|  | Autistic Adult (n=469) | | Other/No answer (n=17) |
| --- | --- | --- | --- |
|  | Male (n=144) | Female (n=308) |
| Adult satisfied | 64 (44.4) | 119 (38.6) | 7 (41.2) |
| Help in own home | 28 (19.4) | 92 (29.9) | 5 (29.4) |
| Day center | 9 (6.3) | 12 (3.9) | 1 (5.9) |
| Full time residential facility | 5 (3.5) | 12 (3.9) | 1 (5.9) |
| Help at college or school dormitory | 4 (2.8) | 5 (1.6) | 1 (5.9) |
| Caregiver respite care | 8 (5.6) | 12 (3.9) | 1 (5.9) |
| Other | 26 (18.1) | 56 (18.2) | 1 (5.9) |

*Note*. Values expressed as number of responders and frequencies (in parenthesis). NA=Question not available for the correspondent group. Data are for all adults and carers of adults. The question for the autistic adults was: “*If you could choose a residential service that fits your needs best now, what would you choose?(Please, tick 1 box)”.*

**Supplementary Data 5a**

*Preferred employment services*

|  | Autistic adult (n=463) | Carer (n=432) | | Professional (n=198) |
| --- | --- | --- | --- | --- |
|  |  | High independence (n=217) | Low independence (n=215) |  |
|  | N (%) | N (%) | N (%) | N (%) |
| Adult satisfied | 112 (24.1) | 45 (20.7) | 44 (20.4) | NA |
| I prefer not to make a choice | NA | NA | NA | 22 (11.1)* |
| Employment skills training | 31 (6.7) | 14 (6.4) | 8 (3.7) | 16 (9.0) |
| Community job center | 7 (1.5) | 3 (1.3) | 3 (1.4) | 1 (0.5) |
| Job placement specific for persons with autism spectrum | 63 (13.5) | 28 (12.9) | 22 (10.2) | 26 (14.7) |
| Employment counseling specific for persons with autism spectrum | 56 (12.1) | 19 (8.7) | 10 (4.6) | 9 (5.1) |
| Employer programs to encourage employment of persons with autism | NA | NA | NA | 27 (15.3) |
| Internships or work placement | 17 (3.6) | 10 (4.6) | 3 (1.4) | 11 (6.2) |
| Sheltered employment training | 7 (1.5) | 7 (3.2) | 9 (4.1) | 13 (7.3) |
| Sheltered employment | 24 (5.1) | 19 (8.7) | 42 (19.5) | 13 (7.3) |
| Employer training about autism | NA | NA | NA | 19 (10.8) |
| Reasonable adjustments in the workplace for employees with autism | NA | NA | NA | 9 (5.1) |
| Job mentors | 84 (18.1) | 54 (24.8) | 40 (18.6) | 32 (18.1) |
| Other | 62 (13.3) | 18 (8.2) | 34 (15.8) | NA |

*Note*. NA=Question not available for the correspondent group. Data are for all adults and carers of adults. The question for the autistic adults was: “*If you could choose an employment service that fits your needs best now, what would you choose? (Please, tick 1 box)”.* The question for the carers of autistic adults was: *“If you could choose an employment service that fits the adult’s needs best now, what would you choose? (Please, tick 1 box)”.* The carers’ data are stratified by high and low level of independence of the autistic adult. The question for the professional was: *“Which 2 types of employment services do you think are most needed for autistic adults in the (geographical) area where you work now? (Please, tick 2 boxes).”* Professionals who selected “*I prefer not to make a choice*” are not included in the calculation of % for each of the other answer choices. The %s for the other answer choices are based on the professionals who made a choice, n=176 (198-22=176). *This % is calculated on the total sample of the professionals’ responders (n=198).

**Supplementary Data 5b**

Employment services availability

|  | Kind of employment service that was applied for, but failed | | | Kind of employment service the adult got | | | Employment service availability | | | | |
| --- | --- | --- | --- | --- | --- | --- | --- | --- | --- | --- | --- |
|  | Autistic adult (n=43) | Carer  High independence (n=21) | Carer  Low independence (n=20) | Autistic adult (n=131) | Carer  High indipendece  (n=80) | Carer  Low indipendence  (n=48) | Professional (n=115) | | | | |
|  |  |  |  |  |  |  | Currently available | Not available now, but there are plans in motion to make it available | Not available now, but it is available in other areas of the country | Not available now and, to my knowledge, there are no plans to make it available | Don't know |
| Employment skills training | 13 (13.13) | 5 (11.90) | 3 (7.32) | 24 (10.00) | 21 (13.29) | 9 (11.69) | 85 (73.91) | 4 (3.48) | 6 (5.22) | 2 (1.74) | 18 (15.65) |
| Community job center | 8 (8.08) | 6 (14.29) | 2 (4.88) | 37 (15.42) | 20 (12.66) | 4 (5.19) | 82 (71.30) | 1 (0.87) | 6 (5.22) | 7 (6.09) | 19 (16.52) |
| Job placement specific for persons with autism spectrum | 15 (15.15) | 4 (9.52) | 6 (14.63) | 9 (3.75) | 5 (3.16) | 5 (6.49) | 57 (49.57) | 9 (7.83) | 11 (9.57) | 12 (10.43) | 26 (22.61) |
| Employment counseling specific for persons with autism spectrum | 17 (17.17) | 4 (9.52) | 7 (17.07) | 10 (4.17) | 6 (3.80) | 5 (6.49) | 57 (49.57) | 8 (6.96) | 13 (11.30) | 9 (7.83) | 28 (24.35) |
| Internships or work placement | 10 (10.10) | 7 (16.67) | 3 (7.32) | 52 (21.67) | 34 (21.52) | 10 (12.99) | NA | NA | NA | NA | NA |
| Employer programs to encourage employment of persons with autism | NA | NA | NA | NA | NA | NA | 22 (19.13) | 16 (13.91) | 10 (8.70) | 13 (11.30) | 54 (46.96) |
| Sheltered employment training | 8 (8.08) | 4 (9.52) | 2 (4.88) | 15 (6.25) | 10 (6.33) | 6 (7.79) | 80 (69.57) | 5 (4.35) | 4 (3.48) | 7 (6.09) | 19 (16.52) |
| Sheltered employment | 5 (5.05) | 5 (11.90) | 4 (9.76) | 14 (5.83) | 7 (4.43) | 14 (18.18) | 89 (77.39) | 6 (5.22) | 2 (1.74) | 6 (5.22) | 12 (10.43) |
| Job mentors | 16 (16.16) | 5 (11.90) | 11 (26.83) | 45 (18.75) | 31 (19.62) | 14 (18.18) | 88 (76.52) | 6 (5.22) | 2 (1.74) | 5 (4.35) | 14 (12.17) |
| Reasonable adjustments in the workplace for employees with autism | NA | NA | NA | NA | NA | NA | 51 (44.35) | 9 (7.83) | 7 (6.09) | 11 (9.57) | 37 (32.17) |
| Other | 7 (7.07) | 2 (4.76) | 3 (7.32) | 34 (14.17) | 24 (15.19) | 10 (12.99) | NA | NA | NA | NA | NA |

*Note*. Values expressed as number of responders and frequencies (in parenthesis). NA=Question not available for the correspondent group. Kind of employment service that was applied for but failed: data are for adults and carers of adults who were not in an employmentservice now or in the last 2 years but had tried to get an employment service in the last 2 years and failed. Kind of employment service the adult got: data are for adults and carers of adults who were in an employment service now or in the last 2 years. The question for the autistic adults was: “*What kind of employment service were you trying to get? (Check all that apply).”* The question for the carers of autistic adults was: *“what kind of employment service did the adult, or someone for the adult, try to get? (Check all that apply)”.* The carers’ data are stratified by high and low level of independence of the autistic adult. The question for the professionals was: *“For each type of adult employment service available for autistic persons, select the availability that best fits what you know.”*

**Supplementary Data 5c**

*Employment services ava*ilability by gender

|  | Kind of employment service that was applied for, but failed  (43 Autistic Adult;14 male, 28 female, 1 other/no answer) | | | Kind of employment service the adult got  (131 Autistic Adult;46 male, 82 female, 3 other/no answer) | | |
| --- | --- | --- | --- | --- | --- | --- |
|  | Male | Female | Other/No answer | Male | Female | Other/No answer |
|
| Employment skills training | 7 (4.9) | 6(2.0) | 0 (0.0) | 11 (7.6) | 13 (4.2) | 0 (0.0) |
| Community job center | 3 (2.1) | 5 (1.6) | 0 (0.0) | 9 (6.3) | 27 (8.8) | 1 (4.2) |
| Job placement specific for persons with autism spectrum | 5 (3.5) | 10 (3.3) | 0 (0.0) | 6 (4.2) | 3 (1.0) | 0 (0.0) |
| Employment counseling specific for persons with autism spectrum | 4 (2.8) | 13 (4.2) | 0 (0.0) | 4 (2.8) | 6 (2.0) | 0 (0.0) |
| Internships or work placement | 2 (1.4) | 8 (2.6) | 0 (0.0) | 21 (14.6) | 29 (9.4) | 2 (8.3) |
| Sheltered employment training | 3 (2.1) | 5 (1.6) | 0 (0.0) | 5 (3.5) | 10 (3.3) | 0 (0.0) |
| Sheltered employment | 1 (0.7) | 4 (1.3) | 0 (0.0) | 10 (6.9) | 4 (1.3) | 0 (0.0) |
| Job mentors | 5 (3.6) | 11 (3.6) | 0 (0.0) | 14 (9.7) | 31 (10.1) | 0 (0.0) |
| Other | 1 (0.7) | 5 (1.6) | 1 (4.2) | 9 (6.3) | 24 (7.8) | 1 (5.9) |

*Note*. Values expressed as number of responders and frequencies (in parenthesis). NA=Question not available for the correspondent group. Kind of employment service that was applied for but failed: data are for adults and carers of adults who were not in an employmentservice now or in the last 2 years but had tried to get an employment service in the last 2 years and failed. Kind of employment service the adult got: data are for adults and carers of adults who were in an employment service now or in the last 2 years. The question for the autistic adults was: “*What kind of employment service were you trying to get? (Check all that apply).”*

**Supplementary Data 5d**

***Preference of employment services by gender***

|  | Autistic Adult (n=463) | | Other/No answer (n=17) |
| --- | --- | --- | --- |
|  | Male (n=143) | Female (n=303) |
| Adult satisfied | 39 (27.3) | 70 (23.1) | 3 (17.7) |
| Employment skills training | 13 (9.1) | 17 (5.6) | 1 (5.9) |
| Community job center | 1 (0.7) | 6 (2.0) | 0 (0.0) |
| Job placement specific for persons with autism spectrum | 23 (16.1) | 40 (13.2) | 0 (0.0) |
| Employment counseling specific for persons with autism spectrum | 16 (11.2) | 37 (12.2) | 3 (17.7) |
| Internships or work placement | 4 (2.8) | 12 (4.0) | 1 (5.9) |
| Sheltered employment training | 2 (1.40) | 5 (1.7) | 0 (0.0) |
| Sheltered employment | 8 (5.6) | 15 (4.9) | 1 (5.9) |
| Job mentors | 26 (18.2) | 54 (17.8) | 4 (23.5) |
| Other | 11 (7.7) | 47 (15.5) | 4 (23.5) |

*Note*. Values expressed as number of responders and frequencies (in parenthesis). NA=Question not available for the correspondent group. Data are for all adults and carers of adults. The question for the autistic adults was: “*If you could choose an employment service that fits your needs best now, what would you choose?(Please, tick 1 box)”.*

**Supplementary Data 6a**

***Preference adult education services***

|  | Autistic adult (n=458) | Carer (n=424) | | Professional (n=124) |
| --- | --- | --- | --- | --- |
|  |  | High independence (n=213) | Low independence (n=211) |  |
|  | N (%) | N (%) | N (%) | N (%) |
| Adult satisfied | 164 (35.8) | 62 (29.1) | 72 (34.1) | NA |
| I prefer not to make a choice | NA | NA | NA | 13 (10.4)***** |
| Day school or college for adults with autism spectrum | 68 (14.8) | 35 (16.4) | 31 (14.6) | 41 (36.9) |
| Boarding school or college for adults with autism spectrum | 16 (3.4) | 9 (4.2) | 16 (7.5) | 15 (13.5) |
| Mentorship or specialist support in regular education settings | 138 (30.1) | 92 (43.1) | 53 (25.1) | 47 (42.3) |
| Other | 72 (15.7) | 15 (7.0) | 39 (18.4) | 8 (7.2) |

*Note*. NA=Question not available for the correspondent group. Data are for all adults and carers of adults. The question for the autistic adults was: “*If you could choose an education service that fits your needs best now, what would you choose? (Please, tick 1 box)”.* The question for the carers of autistic adults was: *“If you could choose an education service that fits the adult’s needs best now, what would you choose? (Please, tick 1 box)”.* The carers’ data are stratified by high and low level of independence of the autistic adult. The question for the professional was: *“Which 2 types of education services do you think are most needed for autistic adults in the (geographical) area where you work now? (Please, tick 2 boxes).”* Professionals who selected “*I prefer not to make a choice*” are not included in the calculation of % for each of the other answer choices. The %s for the other answer choices are based on the professionals who made a choice, n=111 (124-13=111). *This % is calculated on the total sample of the professionals’ responders (n=124).

**Supplementary Data6b**

*Adult educational services availability*

|  | Kind of adult educational service that was applied for, but failed | | | Kind of adult educational service the adult got | | | Adult educational availability | | | | |
| --- | --- | --- | --- | --- | --- | --- | --- | --- | --- | --- | --- |
|  | Autistic adult (n=27) | Carer  High independence  (n=13) | Carer  High independence  (n=26) | Autistic adult (n=67) | Carer  High independence (n=73) | Carer  Low independence (n=56) | Professional (n=76) | | | | |
|  |  |  |  |  |  |  | Currently available | Not available now, but there are plans in motion to make it available | Not available now, but it is available in other areas of the country | Not available now and, to my knowledge, there are no plans to make it available | Don't know |
| Day school or college for adults with autism spectrum | 7 (24.14) | 4 (26.67) | 6 (17.65) | 11 (15.28) | 18 (23.38) | 14 (23.33) | 52 (68.42) | 3 (3.95) | 1 (1.32) | 5 (6.58) | 15 (19.74) |
| Boarding school or college for adults with autism spectrum | 0 | 1 (6.67) | 5 (14.71) | 3 (4.17) | 3 (3.90) | 5 (8.33) | 27 (35.53) | 4 (5.26) | 7 (9.21) | 9 (11.84) | 29 (38.16) |
| Mentorship or specialist support in regular education settings | 13 (44.83) | 5 (33.33) | 14 (41.18) | 39 (54.17) | 29 (37.66) | 12 (20.00) | 50 (65.79) | 3 (3.95) | 4 (5.26) | 3 (3.95) | 16 (21.05) |
| Other | 9 (31.03) | 5 (33.33) | 9 (26.47) | 19 (26.39) | 27 (35.06) | 29 (48.33) | NA | NA | NA | NA | NA |

*Note*. Values expressed as number of responders and frequencies (in parenthesis). NA=Question not available for the correspondent group. Kind of adult education service that was applied for but failed: data are for adults and carers of adults who were not in an adult education service now or in the last 2 years but had tried to get an adult education service in the last 2 years and failed. Kind of adult education service the adult got: data are for adults and carers of adults who were in an adult education service now or in the last 2 years. The question for the autistic adults was: “*What kind of adult education service were you trying to get? (Check all that apply).”* The question for the carers of autistic adults was: *“what kind of education service did the adult, or someone for the adult, try to get? (Check all that apply)”.* The carers’ data are stratified by high and low level of independence of the autistic adult. The question for the professionals was: *“For each type of adult education service available for autistic persons, select the availability that best fits what you know.”*

**Supplementary Data 6c**

*Adult educational serv*ices availability by gender

|  | Kind of adult educational service that was applied for, but failed  (27 Autistic Adult, 13 male,13 female, 1 Other/No answer) | | | Kind of adult educational service the adult got  (67 Autistic Adult, 19 male, 45female, 3 Other/No answer) | | |
| --- | --- | --- | --- | --- | --- | --- |
|  | Male | Female | Other/No answer | Male | Female | Other/No answer |
|
| Day school or college for adults with autism spectrum | 4 (2.8) | 2 (0.7) | 1 (5.9) | 5 (3.5) | 5 (1.6) | 1 (5.9) |
| Boarding school or college for adults with autism spectrum | 0 (0.0) | 0 (0.0) | 0 (0.0) | 2 (1.4) | 1 (0.3) | 0 (0.0) |
| Mentorship or specialist support in regular education settings | 6 (4.2) | 6 (2.0) | 1 (4.2) | 9 (6.3) | 28 (9.1) | 2 (8.3) |
| Other | 4 (2.8) | 5 (1.6) | 0 (0.0) | 6 (4.2) | 13 (4.2) | 0 (0.0) |

*Note*. Values expressed as number of responders and frequencies (in parenthesis). NA=Question not available for the correspondent group. Kind of adult education service that was applied for but failed: data are for adults and carers of adults who were not in anadult education service now or in the last 2 years but had tried to get an adult education service in the last 2 years and failed. Kind of adult education service the adult got: data are for adults and carers of adults who were in an adult education service now or in the last 2 years. The question for the autistic adults was: “*What kind of adult education service were you trying to get? (Check all that apply).”*

**Supplementary Data 6d**

*Preference of adult educational services by gender*

|  | Autistic Adult (n=458) | | Other/No answer (n=17) |
| --- | --- | --- | --- |
|  | Male (n=142) | Female (n=299) |
| Adult satisfied | 53 (37.3) | 106 (35.5) | 5 (29.4) |
| Day school or college for adults with autism spectrum | 26 (18.3) | 39 (13.0) | 3 (17.7) |
| Boarding school or college for adults with autism spectrum | 6 (4.2) | 8 (2.7) | 2 (11.8) |
| Mentorship or specialist support in regular education settings | 38 (26.8) | 94 (31.4) | 6 (35.3) |
| Other | 19 (13.4) | 52 (17.4) | 1 (5.9) |

*Note*. Values expressed as number of responders and frequencies (in parenthesis). NA=Question not available for the correspondent group. Data are for all adults and carers of adults. The question for the autistic adults was: “*If you could choose an education service that fits your needs best now, what would you choose?(Please, tick 1 box)”.*

**Supplementary Data 7a**

*Preferred financial services*

|  | Autistic adult (n=453) | Carer (n=418) | | Professional (n=88) |
| --- | --- | --- | --- | --- |
|  |  | High independence  (n=211) | Low independence (n=207) |  |
|  | N (%) | N (%) | N (%) | N (%) |
| Adult satisfied | 130 (28.7) | 46 (21.8) | 48 (23.1) | NA |
| I prefer not to make a choice | NA | NA | NA | 10 (11.3)* |
| Stipend during school or job training | 37 (8.1) | 29 (13.7) | 5 (2.4) | 13 (16.7) |
| Unemployment benefits | 16 (3.5) | 6 (2.8) | 0 | 6 (7.6) |
| Supplementary income for persons unable to have full employment | 79 (17.4) | 26 (12.3) | 22 (10.6) | 15 (19.2) |
| Full pension | 56 (12.3) | 26 (12.3) | 53 (25.6) | 11 (14.1) |
| Special 'insurance' to help pay for health care | 21 (4.6) | 2 (0.9) | 6 (2.9) | 1 (1.2) |
| Transportation benefits | 11 (2.4) | 3 (1.4) | 6 (2.9) | 3 (3.8) |
| Caregiver supplementary income | 18 (3.9) | 19 (9.0) | 32 (15.4) | 4 (5.1) |
| Supported employment | 43 (9.4) | 45 (21.3) | 18 (8.7) | 21 (26.9) |
| Other | 42 (9.2) | 9 (4.2) | 17 (8.2) | 4 (5.1) |

*Note*. NA=Question not available for the correspondent group. Data are for all adults and carers of adults. The question for the autistic adults was: “*If you could choose a financial service that fits your needs best now, what would you choose? (Please, tick 1 box)”.* The question for the carers of autistic adults was: *“If you could choose a financial service that fits the adult’s needs best now, what would you choose? (Please, tick 1 box)”.* The carers’ data are stratified by high and low level of independence of the autistic adult. The question for the professional was: *“Which 2 types of financial services do you think are most needed for autistic adults in the (geographical) area where you work now? (Please, tick 2 boxes).”* Professionals who selected “*I prefer not to make a choice*” are not included in the calculation of % for each of the other answer choices. The %s for the other answer choices are based on the professionals who made a choice, n=78 (88-10=78). *This % is calculated on the total sample of the professionals’ responders (n=88).

**Supplementary Data 7b**

*Financial services* availability

|  | Kind of financial service that was applied for, but failed | | | Kind of financial service the adult got | | | Financial service availability | | | | |
| --- | --- | --- | --- | --- | --- | --- | --- | --- | --- | --- | --- |
|  | Autistic adult (n=49) | Carer  High indipendence (n=19) | Carer  Low indipendence (n=15) | Autistic adult (n=197) | Carer  High indipendence (n=123) | Carer  Low indipendence (n=147) | Professional (n=52) | | | | |
|  |  |  |  |  |  |  | Currently available | Not available now, but there are plans in motion to make it available | Not available now, but it is available in other areas of the country | Not available now and, to my knowledge, there are no plans to make it available | Don't know |
| Stipend/support during school or job training | 4 (5.26) | 6 (26.09) | 1 (4.76) | 39 (14.08) | 27 (14.29) | 18 (8.82) | 37 (71.15) | 3 (5.77) | 2 (3.85) | 1 (1.92) | 9 (17.31) |
| Unemployment benefits | 8 (10.53) | 3 (13.04) | 1 (4.76) | 62 (22.38) | 21 (11.11) | 6 (2.94) | 38 (73.08) | 2 (3.85) | 0 | 3 (5.77) | 9 (17.31) |
| Supplementary income for persons unable to have full employment | 14 (18.42) | 1 (4.35) | 1 (4.76) | 27 (9.75) | 20 (10.58) | 5 (2.45) | 38 (73.08) | 1 (1.92) | 1 (1.92) | 6 (11.54) | 6 (11.54) |
| Full pension | 10 (13.16) | 1 (4.35) | 2 (9.52) | 45 (16.25) | 37 (19.58) | 80 (39.22) | 39 (75.00) | 2 (3.85) | 0 | 1 (1.92) | 10 (19.23) |
| Special 'insurance' to help pay for health care | 6 (7.89) | 2 (8.70) | 2 (9.52) | 7 (2.53) | 6 (3.17) | 6 (2.94) | 15 (28.85) | 2 (3.85) | 1 (1.92) | 5 (9.62) | 29 (55.77) |
| Transportation benefits Transportation benefits | 7 (9.21) | 2 (8.70) | 1 (4.76) | 22 (7.94) | 22 (11.64) | 24 (11.76) | 41 (78.85) | 1 (1.92) | 2 (3.85) | 5 (9.62) | 3 (5.77) |
| Caregiver supplementary income | 5 (6.58) | 4 (17.39) | 9 (42.86) | 8 (2.89) | 17 (8.99) | 33 (16.18) | 27 (51.92) | 1 (1.92) | 1 (1.92) | 6 (11.54) | 17 (32.69) |
| Supported employment | 3 (3.95) | 1 (4.35) | 0 | 13 (4.69) | 8 (4.23) | 1 (0.49) | 37 (71.15) | 2 (3.85) | 1 (1.92) | 0 | 12 (23.08) |
| Other | 19 (25.00) | 3 (13.04) | 4 (19.05) | 54 (19.49) | 31 (16.40) | 31 (15.19) | NA | NA | NA | NA | NA |

*Note*. Values expressed as number of responders and frequencies (in parenthesis). NA=Question not available for the correspondent group. Kind of financial service that was applied for but failed: data are for adults and carers of adults who were not in a financial service now or in the last 2 years but had tried to get a financial service in the last 2 years and failed. Kind of financial service the adult got: data are for adults and carers of adults who were in a financial service now or in the last 2 years. The question for the autistic adults was: “*what kind of financial service were you trying to get? (Check all that apply).”* The question for the carers of autistic adults was: *“what kind of financial service did the adult, or someone for the adult, try to get? (Check all that apply)”.* The carers’ data are stratified by high and low level of independence of the autistic adult. The question for the professionals was: *“For each type of adult financial service available for autistic persons, select the availability that best fits what you know.”*

**Supplementary Data 7c**

Financial services availability by gender

|  | Kind of financial service that was applied for, but failed  (49 Autistic Adult, 19 male, 30 female, 0 Other/No answer) | | | Kind of financial service the adult got  (197 Autistic, 55 male, 135 female, 7 Other/No Answer) | | |
| --- | --- | --- | --- | --- | --- | --- |
|  | Male | Female | Other/No answer | Male | Female | Other/No answer |
|
| Stipend/support during school or job training | 3 (2.1) | 1 (0.3) | 0 (0.0) | 11 (7.6) | 25 (8.1) | 3 (17.7) |
| Unemployment benefits | 4 (2.8) | 4 (1.3) | 0 (0.0) | 12 (8.3) | 48 (15.6) | 2 (11.8) |
| Supplementary income for persons unable to have full employment | 4 (2.8) | 10 (3.3) | 0 (0.0) | 7 (4.9) | 20 (6.5) | 0 (0.0) |
| Full pension | 2 (1.4) | 8 (2.6) | 0 (0.0) | 11 (7.6) | 31 (10.1) | 3 (17.7) |
| Special 'insurance' to help pay for health care | 4 (2.8) | 2 (0.7) | 0 (0.0) | 3 (2.1) | 4 (1.3) | 0 (0.0) |
| Transportation benefits Transportation benefits | 5 (3.5) | 2 (0.7) | 0 (0.0) | 8 (5.6) | 14 (4.6) | 0 (0.0) |
| Caregiver supplementary income | 5 (3.5) | 0 (0.0) | 0 (0.0) | 2 (1.4) | 6 (2.0) | 0 (0.0) |
| Supported employment | 3 (2.1) | 0 (0.0) | 0 (0.0) | 4 (2.8) | 9 (2.9) | 0 (0.0) |
| Other | 5 (3.5) | 14 (4.6) | 0 (0.0) | 18 (12.5) | 36 (11.7) | 0 (0.0) |

*Note*. Values expressed as number of responders and frequencies (in parenthesis). NA=Question not available for the correspondent group. Kind of financial service that was applied for but failed: data are for adults and carers of adults who were not in a financial service now or in the last 2 years but had tried to get a financial service in the last 2 years and failed. Kind of financial service the adult got: data are for adults and carers of adults who were in a financial service now or in the last 2 years. The question for the autistic adults was: “*what kind of financial service were you trying to get? (Check all that apply).”*

**Supplementary Data 7d**

*Preference of financial servicesby gender*

|  | Autistic Adult (n=453) | | Other/No answer (n=17) |
| --- | --- | --- | --- |
|  | Male(n=141) | Female (n=295) |
| Adult satisfied | 44 (31.2) | 79 (26.8) | 7 (41.2) |
| Stipend during school or job training | 14 (9.9) | 21 (7.1) | 2 (11.8) |
| Unemployment benefits | 7 (5.0) | 8 (2.7) | 1 (5.9) |
| Supplementary income for persons unable to have full employment | 17 (12.1) | 61 (20.7) | 1 (5.9) |
| Full pension | 11 (7.8) | 42 (14.2) | 3 (17.7) |
| Special 'insurance' to help pay for health care | 4 (2.8) | 17 (5.8) | 0 (0) |
| Transportation benefits | 1 (0.7) | 10 (3.4) | 0 (0.0) |
| Caregiver supplementary income | 12 (8.5) | 6 (2.0) | 0 (0.0) |
| Supported employment | 21 (14.9) | 21 (7.1) | 1 (5.9) |
| Other | 10 (7.1) | 30 (10.2) | 2 (11.8) |

*Note*. Values expressed as number of responders and frequencies (in parenthesis). NA=Question not available for the correspondent group. Data are for all adults and carers of adults. The question for the autistic adults was: “*If you could choose a financial service that fits your needs best now, what would you choose? (Please, tick 1 box)”.*

**Supplementary Data 8a**

***Preference social support services***

|  | Autistic adult (n=444) | Carer (n=413) | | Professional  (n=207) |
| --- | --- | --- | --- | --- |
|  |  | High independence (n=207) | Low independence (n=206) |  |
|  | N (%) | N (%) | N (%) | N (%) |
| Adult satisfied | 115 (25.9) | 25 (12.0) | 38 (18.4) | NA |
| I prefer not to make a choice | NA | NA | NA | 21 (10.1)* |
| Life skills training | 45 (10.1) | 37 (17.8) | 47 (22.8) | 60 (32.2) |
| Support groups, on-line | 9 (2.0) | 3 (1.4) | 0 | 5 (2.6) |
| Support groups, in person | 31 (6.9) | 8 (3.8) | 1 (0.4) | 14 (7.5) |
| Free time activities | 34 (7.6) | 20 (9.6) | 30 (14.5) | 23 (12.3) |
| Family relations support and counseling | 31 (6.9) | 12 (5.8) | 9 (4.3) | 14 (7.5) |
| Behavior training, for an individual | 51 (11.4) | 19 (9.1) | 29 (14.0) | 23 (12.3) |
| Behavior training, in groups | 9 (2.0) | 16 (7.7) | 5 (2.4) | 12 (6.4) |
| Program for matching autistic peers with each other | 49 (11.0) | 19 (9.1) | 12 (5.8) | 10 (5.3) |
| Program for matching an autistic person with a non-autistic peer | 36 (8.1) | 37 (17.8) | 26 (12.6) | 13 (6.9) |
| Other | 34 (7.6) | 11 (5.3) | 9 (4.3) | 12 (6.4) |

*Note*. NA=Question not available for the correspondent group. Data are for all adults and carers of adults. The question for the autistic adults was: “*If you could choose a social support* *service that fits your needs best now, what would you choose? (Please, tick 1 box)”.* The question for the carers of autistic adults was: *“If you could choose a social support service that fits the adult’s needs best now, what would you choose? (Please, tick 1 box)”.* The carers’ data are stratified by high and low level of independence of the autistic adult. The question for the professional was: *“Which 2 types of social support services do you think are most needed for autistic adults in the (geographical) area where you work now? (Please, tick 2 boxes).”* Professionals who selected “*I prefer not to make a choice*” are not included in the calculation of % for each of the other answer choices. The %s for the other answer choices are based on the professionals who made a choice, n=186 (207-21=186). *This % is calculated on the total sample of the professionals’ responders (n=207).

**Supplementary Data 8b**

Social support services availability

|  | Kind of social support service that was applied for, but failed | | | Kind of social support service the adult got | | | Social support service availability | | | | |
| --- | --- | --- | --- | --- | --- | --- | --- | --- | --- | --- | --- |
|  | Autistic adult (n=37) | High independence (n=35) | Low independence (n=31) | Autistic adult (n=142) | High independence (n=73) | Low independence (n=80) | Professional (n=118) | | | | |
|  |  |  |  |  |  |  | Currently available | Not available now, but there are plans in motion to make it available | Not available now, but it is available in other areas of the country | Not available now and, to my knowledge, there are no plans to make it available | Don't know |
| Life skills training | 15 (14.29) | 9 (12.86) | 16 (19.28) | 57 (21.92) | 40 (24.84) | 38 (21.35) | 104 (88.14) | 0 | 2 (1.69) | 4 (3.39) | 8 (6.78) |
| Support groups, on-line | 3 (2.86) | 0 | 1 (1.20) | 11 (4.23) | 1 (0.62) | 1 (0.56) | 25 (21.19) | 7 (5.93) | 9 (7.63) | 17 (14.41) | 60 (50.85) |
| Support groups, in person | 8 (7.62) | 8 (4.71) | 6 (7.23) | 33 (12.69) | 18 (11.18) | 16 (8.99) | 72 (61.02) | 5 (4.249 | 6 (5.08) | 6 (5.08) | 29 (24.58) |
| Free time activities | 16 (15.24) | 11 (15.71) | 14 (16.87) | 37 (14.23) | 21 (13.04) | 38 (21.35) | 94 (79.66) | 2 (1.69) | 1 (0.85) | 8 (6.78) | 13 (11.02) |
| Family relations support and counseling | 14 (13.33) | 7 (10.00) | 6 (7.23) | 22 (8.46) | 9 (5.59) | 11 (6.18) | 68 (57.63) | 4 (3.39) | 2 (1.69) | 9 (7.63) | 35 (29.66) |
| Behavior training, for an individual | 14 (13.33) | 7 (10.00) | 11 (13.25) | 27 (10.38) | 20 (12.42) | 22 (12.36) | 62 (52.54) | 0 | 7 (5.93) | 13 (11.02) | 36 (30.51) |
| Behavior training, in groups | 8 (7.62) | 3 (4.29) | 7 (8.43) | 13 (5.00) | 12 (7.45) | 18 (10.11) | 51 (43.22) | 1 (0.85) | 7 (5.93) | 14 (11.86) | 45 (38.14) |
| Program for matching autistic peers with each other | 12 (11.43) | 6 (8.57) | 7 (8.43) | 21 (8.08) | 10 (6.21) | 10 (5.62) | 34 (28.81) | 12 (10.07) | 3 (2.54) | 16 (13.56) | 53 (44.92) |
| Program for matching an autistic person with a non-autistic peer | 10 (9.52) | 8 (11.43) | 9 (10.84) | 2 (0.77) | 11 (6.83) | 8 (4.49) | 32 (27.12) | 6 (5.08) | 4 (3.39( | 19 (16.10) | 57 (48.31) |
| Other | 5 (4.76) | 11 (15.71) | 6 (7.23) | 37 (14.23) | 19 (11.80) | 16 (8.99) | NA | NA | NA | NA | NA |

*Note*. Values expressed as number of responders and frequencies (in parenthesis). NA=Question not available for the correspondent group. Kind of social support service that was applied for, but failed: data are for adults and carers of adults who were not in a social support service now or in the last 2 years, but had tried to get a social support service in the last 2 years and failed. Kind of social support service the adult got: data are for adults and carers of adults who were in a social support service now or in the last 2 years. The question for the autistic adults was: “*what kind of social supportservice were you trying to get? (Check all that apply).”* The question for the carers of autistic adults was: *“what kind of social supportservice did the adult, or someone for the adult, try to get? (Check all that apply)”.* The carers’ data are stratified by high and low level of independence of the autistic adult. The question for the professionals was: *“For each type of adult social support service available for autistic persons, select the availability that best fits what you know.”*

**Supplementary Data 8c**

Social support services availability by gender

|  | Kind of social support service that was applied for, but failed  (37 Autistic Adult, 11 male, 24 female, 2 Other/No Answer) | | | Kind of social support service the adult got  (142 Autistic Adult, 39 male, 98 female, 5 Other/No Answer) | | |
| --- | --- | --- | --- | --- | --- | --- |
|  | Male | Female | Other/No Answer | Male | Female | Other/No Answer |
|
| Life skills training | 3 (2.1) | 11 (3.6) | 1 (5.9) | 21 (14.6) | 34 (11.0) | 2 (11.8) |
| Support groups, on-line | 0 (0.0) | 2 (0.7) | 1 (5.9) | 4 (2.8) | 6 (2.0) | 1 (5.9) |
| Support groups, in person | 2 (1.4) | 5 (1.6) | 1 (5.9) | 5 (3.5) | 25 (8.1) | 3 (17.7) |
| Free time activities | 6 (4.2) | 10 (3.3) | 0 (0.0) | 11 (7.6) | 24 (7.8) | 2 (11.8) |
| Family relations support and counseling | 6 (4.2) | 8 (2.6) | 0 (0.0) | 8 (5.6) | 13 (4.2) | 1 (5.9) |
| Behavior training, for an individual | 5 (3.5) | 8 (2.6) | 1 (5.9) | 5 (3.5) | 21 (6.8) | 1 (5.9) |
| Behavior training, in groups | 2 (1.4) | 5 (1.6) | 1 (5.9) | 4 (2.8) | 8 (2.6) | 1 (5.9) |
| Program for matching autistic peers with each other | 2 (1.4) | 8 (2.6) | 2 (11.8) | 8 (5.6) | 10 (3.3) | 3 (17.7) |
| Program for matching an autistic person with a non-autistic peer | 3 (2.1) | 6 (2.0) | 1 (5.9) | 1 (0.7) | 1 (0.3) | 0 (0.0) |
| Other | 0 (0.0) | 5 (1.6) | 0 (0.0) | 8 (5.6) | 29 (9.4) | 0 (0.0) |

*Note*. Values expressed as number of responders and frequencies (in parenthesis). NA=Question not available for the correspondent group. Kind of social support service that was applied for, but failed: data are for adults and carers of adults who were not in a social support service now or in the last 2 years, but had tried to get a social support service in the last 2 years and failed. Kind of social support service the adult got: data are for adults and carers of adults who were in a social support service now or in the last 2 years. The question for the autistic adults was: “*what kind of social supportservice were you trying to get? (Check all that apply).”*

**Supplementary Data 8d**

*Preference of social support services by gender*

|  | Autistic Adult (n=444) | | Other/No Answer  (n=17) |
| --- | --- | --- | --- |
|  | Male (n=138) | Female (n=289) |
| Adult satisfied | 41 (29.7) | 71 (24.6) | 3 (17.8) |
| Life skills training | 16 (11.6) | 28 (9.7) | 1 (5.9) |
| Support groups, on-line | 1 (0.7) | 7 (2.4) | 1 (5.9) |
| Support groups, in person | 6 (4.4) | 21 (7.3) | 4 (23.5) |
| Free time activities | 14 (10.1) | 20 (6.9) | 0 (0.0) |
| Family relations support and counseling | 11 (8.0) | 20 (6.9) | 0 (0.0) |
| Behavior training, for an individual | 14 (10.1) | 36 (12.5) | 1 (5.9) |
| Behavior training, in groups | 3 (2.2) | 6 (2.1) | 0 (0.0) |
| Program for matching autistic peers with each other | 13 (9.4) | 33 (11.4) | 3 (17.7) |
| Program for matching an autistic person with a non-autistic peer | 12 (8.7) | 20 (6.9) | 4 (23.5) |
| Other | 7 (5.1) | 27 (9.3) | 0 (0.0) |

*Note*. Values expressed as number of responders and frequencies (in parenthesis). NA=Question not available for the correspondent group. Data are for all adults and carers of adults. The question for the autistic adults was: “*If you could choose a social supportservice that fits your needs best now, what would you choose? (Please, tick 1 box)”.*

**Supplementary Data 9**

*Service availability by countries*

|  | Autistic adult |  | Carer |  |
| --- | --- | --- | --- | --- |
| Answer | Yes, I have tried to get the service, and I did NOT succeed | N | Yes, the adult, or someone for the adult, tried to get the service, but did NOT succeed | N |
| **Residential** |  |  |  |  |
| Denmark | 23 (11.92) | 193 | 11 (9.02) | 122 |
| Finland | 6 (9.52) | 63 | 8 (22.22) | 36 |
| France | 7 (11.48) | 61 | 11 (16.42) | 67 |
| Spain | 2 (9.02) | 22 | 5 (5.81) | 86 |
| Italy | 6 (16.67) | 36 | 3 (9.38) | 32 |
| Poland | 1 (2.63) | 38 | 5 (10.42) | 48 |
| Iceland | 0 (0.00) | 16 | 9 (22.50) | 40 |
| United Kingdom | 1 (4.76) | 21 | 0 (0.00) | 6 |
| Republic of Ireland | 1 (7.69) | 13 | 0 (0.00) | 10 |
| Germany | 1 (16.67) | 6 | NA | NA |
| **Employment** |  |  |  |  |
| Denmark | 13 (6.74) | 193 | 11 (9.02) | 122 |
| Finland | 6 (9.52) | 63 | 5 (13.89) | 36 |
| France | 8 (13.11) | 61 | 7 (10.45) | 67 |
| Spain | 1 (4.54) | 22 | 6 (6.98) | 86 |
| Italy | 8 (22.22) | 36 | 5 (12.63) | 32 |
| Poland | 2 (5.26) | 38 | 1 (2.08) | 48 |
| Iceland | 0 (0.00) | 16 | 5 (12.50) | 40 |
| United Kingdom | 3 (14.29) | 21 | 0 (0.00) | 6 |
| Republic of Ireland | 1 (7.69) | 13 | 1 (10.00) | 10 |
| Germany | 1 (16.67) | 6 | NA | NA |
| **Education** |  |  |  |  |
| Denmark | 6 (3.11) | 193 | 9 (7.38) | 122 |
| Finland | 6 (9.52) | 63 | 5 (13.89) | 36 |
| France | 4 (6.58) | 61 | 9 (13.43) | 67 |
| Spain | 1 (4.55) | 22 | 5 (5.81) | 86 |
| Italy | 7 (19.44) | 36 | 3 (9.38) | 32 |
| Poland | 0 (0.00) | 38 | 4 (8.33) | 48 |
| Iceland | 0 (0.00) | 16 | 2 (5.00) | 40 |
| United Kingdom | 1 (4.76) | 21 | 0 (0.00) | 6 |
| Republic of Ireland | 2 (15.38) | 13 | 2 (20.00) | 10 |
| Germany | 0 (0.00) | 6 | NA | NA |
| **Financial** |  |  |  |  |
| Denmark | 11 (5.70) | 193 | 12 (9.84) | 122 |
| Finland | 9 (14.29) | 63 | 3 (8.33) | 36 |
| France | 13 (21.31) | 61 | 5 (7.46) | 67 |
| Spain | 5 (22.73) | 22 | 6 (6.98) | 86 |
| Italy | 5 (13.89) | 36 | 3 (9.38) | 32 |
| Poland | 2 (5.26) | 38 | 2 (4.17) | 48 |
| Iceland | 0 (0.00) | 16 | 2 (5.00) | 40 |
| United Kingdom | 2 (9.52) | 21 | 0 (0.00) | 6 |
| Republic of Ireland | 1 (7.69) | 13 | 1 (10.00) | 10 |
| Germany | 1 (16.67) | 6 | NA | NA |
| **Social support** |  |  |  |  |
| Denmark | 12 (6.22) | 193 | 15 (12.30) | 122 |
| Finland | 5 (7.93) | 63 | 6 (16.67) | 36 |
| France | 10 (16.39) | 61 | 13 (19.40) | 67 |
| Spain | 3 (13.64) | 22 | 8 (9.30) | 86 |
| Italy | 3 (8.33) | 36 | 7 (21.88) | 32 |
| Poland | 0 (0.00) | 38 | 5 (10.42) | 48 |
| Iceland | 1 (6.25) | 16 | 10 (25.00) | 40 |
| United Kingdom | 2 (9.52) | 21 | 0 (0.00) | 6 |
| Republic of Ireland | 1 (7.69) | 13 | 2 (20.00) | 10 |
| Germany | 0 (0.00) | 6 | NA | NA |

*Note*. Values expressed as number of responders and frequencies (in parenthesis). The question for the autistic adults was: “*What kind of residential service were you trying to get? (Check all that apply).”* The question for the autistic adults was: *“Have you tried to get a service at some time in the last 2 years?”.* The question for the carers was: *“Has the adult, or someone for the adult, tried to get the service at some time in the last 2 years?”*

**Supplementary Data 10**

*Recommended r*esidential services provision

|  | Autistic adult (n=137) | | | | Carer (n=216) | | | | Professional (n=173) | | | | | |
| --- | --- | --- | --- | --- | --- | --- | --- | --- | --- | --- | --- | --- | --- | --- |
|  | Yes | No | Does not apply | Don’t know | Yes | No | Does not apply | Don’t know | Yes | No, but there are plans in motion to get it started | No, but I believe that it may be helpful | No, and I believe that it is not needed | Does not apply | Don't know |
| Structured activities for the residents | 71 (51.82) | 35 (25.55) | 22 (16.06) | 9 (6.57) | 137 (63.43) | 48 (22.22) | 17 (7.87) | 14 (6.48) | 145 (83.82) | 6 (3.47) | 9 (5.20) | 1 (0.58) | 2 (1.16) | 10 (5.78) |
| Activities for the residents to feel part of the community | 54 (39.42) | 48 (35.04) | 27 (19.71) | 8 (5.84) | 119 (55.09) | 59 (27.31) | 17 (7.87) | 21 (9.72) | 134 (77.46) | 8 (4.62) | 14 (8.09) | 2 (1.16) | 1 (0.58) | 14 (8.09) |
| Opportunities for the residents to go into different places in the community | 69 (50.36) | 43 (31.39) | 17 (12.41) | 8 (5.84) | 127 (58.80) | 58 (26.85) | 19 (8.80) | 12 (5.56) | 136 (78.61) | 5 (2.89) | 11 (6.36) | 5 (2.89) | 3 (1.73) | 13 (7.51) |
| A physical environment that is adapted to the needs of adults with autism spectrum | 53 (38.69) | 43 (31.39) | 27 (19.71) | 14 (10.22) | 127 (58.80) | 64 (29.63) | 13 (6.02) | 12 (5.56) | 121 (69.94) | 10 (5.78) | 25 (14.45) | 3 (1.73) | 2 (1.16) | 12 (6.94) |
| Staff with specialist autism spectrum training | 65 (47.45) | 49 (35.77) | 8 (5.84) | 15 (10.95) | 128 (59.26) | 63 (29.17) | 7 (3.24) | 18 (8.33) | 136 (78.61) | 8 (4.62) | 15 (8.67) | 1 (0.58) | 1 (0.58) | 12 (6.94) |
| Ways to get specialist care when it is needed | 25 (18.25) | 68 (49.64) | 17 (12.41) | 27 (19.71) | 97 (44.91) | 81 (37.50) | 13 (6.02) | 25 (11.57) | 116 (67.05) | 11 (6.36) | 25 (14.45) | 1 (0.58) | 4 (2.31) | 16 (9.25) |
| Ways to coordinate services with other providers in the area, if needed | 44 (32.12) | 52 (37.96) | 10 (7.30) | 31 (22.63) | 88 (40.74) | 74 (64.26) | 13 (6.02) | 41 (18.98) | 109 (63.01) | 10 (5.78) | 23 (13.29) | 0 | 9 (5.20) | 22 (12.72) |
| Support for employment | NA | NA | NA | NA | 81 (37.50) | 84 (38.89) | 33 (15.28) | 18 (8.33) | NA | NA | NA | NA | NA | NA |
| Support for independent living | NA | NA | NA | NA | 96 (44.44) | 62 (28.70) | 36 (16.67) | 22 (10.19) | NA | NA | NA | NA | NA | NA |

*Note*. Values expressed as number of responders and frequencies (in parenthesis). NA=Question not available for the correspondent group. Data are for adults and carers of adults in a residential service now or in the last 2 years, and for professionals with current work experience with residential services. The question for the autistic adults and carers was: *“Does or did the residential service provide:”.* The question for the professional was: *“To the best of your knowledge, do the residential services for adults in the (geographical) area where you work now provide:”*

**Supplementary Data 11**

Waiting time for services

|  | Autistic adult | | | | | Carer | | | | | Professional | | | | | |
| --- | --- | --- | --- | --- | --- | --- | --- | --- | --- | --- | --- | --- | --- | --- | --- | --- |
|  | < 1 month | 1-3 months | 3-6 months | > 6 months | N | < 1 month | 1-3 months | 3-6 months | > 6 months | N | < 1 month | 1-3 months | 3-6 months | > 6 months | Don't know | N |
| Waiting time for a residential service | | | | | | | | | | | | | | | | |
| Residential service | 30 (25.42) | 37 (31.36) | 24 (20.34) | 27 (22.88) | 128 | 39 (22.67) | 40 (23.26) | 26 (15.12) | 67 (38.95) | 172 | NA | NA | NA | NA | NA | NA |
| Help in own home | 20 (24.69) | 29 (35.80) | 16 (19.75) | 16 (19.75) | 81 | 14 (31.11) | 15 (33.33) | 5 (11.11) | 11 (24.44) | 45 | 43 (26.06) | 37 (22.42) | 10 (6.06) | 14 (8.48) | 61 (36.97) | 165 |
| Day center | 3 (21.43) | 5 (35.70) | 3 (21.43) | 3 (21.43) | 14 | 11 (26.19) | 9 (21.43) | 9 (21.43) | 13 (30.95) | 42 | 32 (19.28) | 32 (19.28) | 6 (3.61) | 31 (18.67) | 65 (39.16) | 166 |
| Full time residential facility | 2 (18.18) | 0 | 3 (27.27) | 6 (54.55) | 11 | 17 (20.99) | 11 (13.58) | 15 (18.52) | 38 (46.91) | 81 | NA | NA | NA | NA | NA | NA |
| Full time residential facility with private bedroom only | NA | NA | NA | NA | NA | NA | NA | NA | NA | NA | 2 (1.27) | 19 (12.10) | 16 (10.19) | 51 (32.48) | 69 (43.95) | 157 |
| Full time residential facility with full apartment | NA | NA | NA | NA | NA | NA | NA | NA | NA | NA | 3 (1.95) | 26 (16.88) | 16 (10.39) | 41 (26.62) | 68 (44.16) | 154 |
| Help at college or school dormitory | 0 | 2 (100.00) | 0 | 0 | 2 | 0 | 1 (25.00) | 1 (25.00) | 2 (50.00) | 4 | 11 (7.10) | 17 (10.97) | 13 (8.39) | 21 (13.55) | 93 (60.00) | 155 |
| Caregiver respite care | 1 (11.11) | 4 (44.44) | 2 (22.22) | 2 (22.22) | 9 | 10 (50.00) | 6 (30.00) | 2 (10.00) | 2 (10.00) | 20 | 18 (11.04) | 30 (18.40) | 14 (8.59) | 10 (6.13) | 91 (55.83) | 163 |
| Other | 10 (30.26) | 9 (29.03) | 6 (19.35) | 6 (19.35) | 31 | 5 (17.24) | 7 (24.14) | 3 (10.34) | 14 (48.28) | 29 | NA | NA | NA | NA | NA | NA |
| Waiting time for an employment service | | | | | | | | | | | | | | | | |
| Employment service | 47 (40.17) | 34 (29.06) | 23 (19.66) | 13 (11.11) | 117 | 35 (35.35) | 22 (22.22) | 19 (19.19) | 23 (23.23) | 99 | NA | NA | NA | NA | NA | NA |
| Employment skills training | 9 (40.91) | 6 (27.27) | 4 (18.18) | 3 (13.64) | 22 | 6 (26.09) | 7 (30.43) | 8 (34.78) | 2 (8.70) | 23 | 18 (18.18) | 23 (23.23) | 6 (6.06) | 7 (7.07) | 45 (45.45) | 99 |
| Community job center | 15 (45.45) | 12 (36.36) | 2 (6.06) | 4 (12.12) | 33 | 9 (47.37) | 7 (36.84) | 2 (10.53) | 1 (5.26) | 19 | NA | NA | NA | NA | NA | NA |
| Job placement specific for persons with autism spectrum | 4 (50.00) | 1 (12.50) | 1 (12.50) | 2 (25.00) | 8 | 3 (42.86) | 2 (28.57) | 1 (14.29) | 1 (14.29) | 7 | 9 (9.68) | 18 (19.35) | 8 (8.60) | 6 (6.45) | 52 (55.91) | 93 |
| Employment counseling specific for persons with autism spectrum | 1 (10.00) | 6 (60.00) | 1 (10.00) | 2 (20.00) | 10 | 3 (30.00) | 2 (20.00) | 3 (30.00) | 2 (20.00) | 10 | 10 (10.42) | 20 (20.83) | 2 (2.08) | 7 (7.29) | 57 (59.38) | 96 |
| Internships or work placement | 12 (26.09) | 19 (41.30) | 9 (19.57) | 6 (13.04) | 46 | 12 (32.43) | 6 (16.22) | 9 (24.32) | 10 (27.03) | 37 | 11 (10.48) | 21 (20.00) | 10 (9.52) | 7 (6.67) | 56 (53.33) | 105 |
| Sheltered employment training | 6 (40.00) | 5 (33.33) | 2 (13.33) | 2 (13.33) | 15 | 4 (26.67) | 4 (26.67) | 3 (20.00) | 4 (26.67) | 15 | 12 (11.88) | 20 (19.80) | 5 (4.95) | 7 (6.93) | 57 (56.44) | 101 |
| Sheltered employment | 5 (38.46) | 4 (30.77) | 2 (15.38) | 2 (15.38) | 13 | 6 (42.86) | 3 (21.43) | 2 (14.29) | 3 (21.43) | 14 | 15 (14.29) | 21 (20.00) | 5 (4.76) | 11 (10.48) | 53 (50.48) | 105 |
| Job mentors | 18 (41.86) | 12 (27.91) | 10 (23.26) | 3 (6.98) | 43 | 11 (29.73) | 9 (24.32) | 8 (21.62) | 9 (24.32) | 37 | 18 (17.65) | 24 (23.53) | 2 (1.96) | 7 (6.86) | 51 (50.00) | 102 |
| Other | 10 (34.48) | 9 (31.03) | 6 (20.69) | 4 (13.79) | 29 | 9 (32.14) | 8 (28.57) | 3 (10.71) | 8 (28.57) | 29 | NA | NA | NA | NA | NA | NA |
| Waiting time for an adult education service | | | | | | | | | | | | | | | | |
| Adult education service | 29 (52.72) | 16 (29.09) | 6 (10.90) | 4 (7.27) | 55 | 40 (40.00) | 25 (25.00) | 11 (11.00) | 24 (24.00) | 100 | NA | NA | NA | NA | NA | NA |
| Day school or college for adults with autism spectrum | 5 (45.45) | 2 (18.18) | 1 (9.09) | 3 (27.27) | 11 | 6 (24.00) | 11 (44.00) | 3 (12.00) | 5 (20.00) | 25 | 8 (11.43) | 7 (10.00) | 5 (7.14) | 8 (11.43) | 42 (60.00) | 70 |
| Boarding school or college for adults with autism spectrum | 1 (33.33) | 2 (66.67) | 0 | 0 | 3 | 3 (42.86) | 3 (42.86) | 0 | 1 (14.29) | 7 | 5 (7.46) | 4 (5.97) | 4 (5.97) | 6 (8.96) | 48 (71.64) | 67 |
| Mentorship or specialist support in regular education settings | 15 (46.88) | 12 (37.50) | 4 (12.50) | 1 (3.13) | 32 | 15 (44.15) | 10 (29.41) | 3 (8.82) | 6 (17.65) | 34 | 11 (15.28) | 10 (13.89) | 1 (1.39) | 7 (9.72) | 43 (59.72) | 72 |
| Other | 9 (64.29) | 2 (14.29) | 2 (14.29) | 1 (7.14) | 14 | 18 (41.86) | 7 (16.28) | 6 (13.95) | 12 (27.91) | 43 | NA | NA | NA | NA | NA | NA |
| Waiting time for a financial service | | | | | | | | | | | | | | | | |
| Financial service | 57 (34.34) | 51 (30.72) | 32 (19.28) | 26 (15.66) | 166 | 56 (24.67) | 74 (32.60) | 37 (16.30) | 60 (26.43) | 227 | NA | NA | NA | NA | NA | NA |
| Stipend/support during school or job training | 9 (27.27) | 14 (42.42) | 8 (24.24) | 2 (6.06) | 33 | 13 (32.50) | 19 (47.50) | 4 (10.00) | 4 (10.00) | 40 | 10 (20.00) | 5 (10.00) | 2 (4.00) | 7 (14.00) | 26 (52.00) | 50 |
| Unemployment benefits | 32 (56.14) | 17 (29.82) | 4 (7.02) | 4 (7.02) | 57 | 9 (39.13) | 9 (39.13) | 3 (13.04) | 2 (8.70) | 23 | 15 (31.25) | 5 (10.42) | 0 | 4 (8.33) | 24 (50.00) | 48 |
| Supplementary income for persons unable to have full employment | 6 (25.00) | 10 (41.67) | 2 (8.33) | 6 (25.00) | 24 | 7 (30.43) | 7 (30.43) | 1 (4.35) | 8 (34.78) | 23 | 8 (17.39) | 6 (13.04) | 1 (2.17) | 5 (10.87) | 26 (56.52) | 46 |
| Full pension | 2 (5.56) | 12 (33.33) | 12 (33.33) | 10 (27.78) | 36 | 22 (23.66) | 31 (33.33) | 14 (15.05) | 26 (27.96) | 93 | 2 (4.00) | 3 (6.00) | 6 (12.00) | 10 (20.00) | 29 (58.00) | 50 |
| Special 'insurance' to help pay for health care | 0 | 3 (60.00) | 0 | 2 (40.00) | 5 | 3 (37.50) | 3 (37.50) | 0 | 2 (25.00) | 8 | 1 (2.13) | 1 (2.13) | 0 | 4 (8.51) | 41 (87.23) | 47 |
| Transportation benefits Transportation benefits | 9 (42.86) | 7 (33.33) | 2 (9.52) | 3 (14.29) | 21 | 4 (11.11) | 20 (55.56) | 4 (11.11) | 8 (22.22) | 36 | 12 (26.09) | 5 (10.87) | 1 (2.17) | 6 (13.04) | 22 (47.83) | 46 |
| Caregiver supplementary income | 1 (14.29) | 1 (14.29) | 3 (42.86) | 2 (28.57) | 7 | 7 (15.91) | 16 (36.36) | 5 (11.36) | 16 (36.36) | 44 | 2 (4.08) | 7 (14.29) | 1 (2.04) | 5 (10.20) | 34 (69.39) | 49 |
| Supported employment | 4 (33.33) | 2 (16.67) | 4 (33.33) | 2 (16.17) | 12 | 1 (12.50) | 4 (50.00) | 0 | 3 (37.50) | 8 | 6 (12.00) | 5 (10.00) | 0 | 5 (10.00) | 34 (68.00) | 50 |
| Other | 17 (37.78) | 14 (31.11) | 8 (17.78) | 6 (13.33) | 45 | 9 (16.97) | 16 (29.63) | 15 (27.78) | 14 (25.93) | 54 | NA | NA | NA | NA | NA | NA |
| Waiting time for a social support service | | | | | | | | | | | | | | | | |
| Social support service | 47 (40.17) | 43 (36.75) | 19 (16.24) | 8 (6.84) | 117 | 53 (44.17) | 33 (27.50) | 14 (11.67) | 20 (16.67) | 120 | NA | NA | NA | NA | NA | NA |
| Life skills training | 15 (30.61) | 23 (46.94) | 7 (14.29) | 4 (8.16) | 49 | 36 (56.25) | 13 (20.31) | 6 (9.38) | 9 (14.06) | 64 | 35 (30.70) | 31 (27.19) | 3 (2.63) | 10 (8.77) | 35 (30.70) | 114 |
| Support groups on-line | 7 (87.50) | 1 (12.50) | 0 | 0 | 8 | 2 (100.00) | 0 | 0 | 0 | 2 | 17 (17.53) | 5 (5.15) | 1 (1.03) | 2 (2.06) | 72 (74.23) | 97 |
| Support groups, in person | 16 (53.33) | 11 (36.67) | 3 (10.00) | 0 | 30 | 13 (44.83) | 5 (17.24) | 6 (20.69) | 5 (17.24) | 29 | 21 (19.44) | 24 (22.22) | 4 (3.70) | 3 (2.78) | 56 (51.85) | 108 |
| Free time activities | 14 (46.67) | 13 (43.33) | 1 (3.33) | 2 (6.67) | 30 | 24 (55.81) | 11 (25.58) | 3 (6.98) | 5 (11.63) | 43 | 32 (29.63) | 20 (18.52) | 3 (2.78) | 4 (3.70) | 49 (45.37) | 108 |
| Family relations support and counseling | 5 (26.35) | 11 (57.89) | 3 (15.79) | 0 | 19 | 11 (64.71) | 2 (11.76) | 0 | 4 (23.53) | 17 | 15 (13.51) | 21 (18.92) | 1 (0.90) | 5 (4.50) | 69 (62.16) | 111 |
| Behavior training, for an individual | 8 (34.78) | 11 (47.83) | 3 (13.04) | 1 (4.35) | 23 | 20 (58.82) | 3 (8.82) | 3 (8.82) | 8 (23.53) | 34 | 13 (12.15) | 18 (16.82) | 1 (0.93) | 6 (5.61) | 69 (64.49) | 107 |
| Behavior training, in groups | 3 (30.00) | 4 (40.00) | 2 (20.00) | 1 (10.00) | 10 | 10 (41.67) | 5 (20.83) | 2 (8.33) | 7 (29.17) | 24 | 7 (6.60) | 20 (18.87) | 2 (1.89) | 4 (3.77) | 73 (68.87) | 106 |
| Program for matching autistic peers with other | 9 (52.94) | 7 (41.18) | 1 (5.88) | 0 | 17 | 5 (35.71) | 3 (21.43) | 2 (14.29) | 4 (28.57) | 14 | 7 (7.29) | 9 (9.38) | 0 | 2 (2.08) | 78 (81.25) | 96 |
| Program for matching an autistic person with non-autistic person | 1 (50.00) | 0 | 1 (50.00) | 0 | 2 | 8 (47.06) | 3 (17.65) | 1 (5.88) | 5 (29.41) | 17 | 4 (4.26) | 4 (4.26) | 0 | 2 (2.13) | 84 (89.36) | 94 |
| Other | 14 (43.75) | 10 (31.25) | 7 (21.88) | 1 (3.13) | 32 | 8 (30.77) | 11 (42.31) | 4 (15.38) | 3 (11.54) | 26 | NA | NA | NA | NA | NA | NA |

*Note*. Values expressed as number of responders and frequencies (in parenthesis). NA=Question not available for the correspondent group. Frequencies are based on respondents who selected a waiting time (excluding respondents who selected the answer choice “Don’t know” or “The service is not currently available” (professionals)). The question (sections in grey) for the autistic adults was: “*What was the waiting time before you could get into the <residential, employment, education, financial, social support> service;* for carers was*:* “*What was the waiting time for the adult to get into the <residential, employment, education, financial, social support>service?*”. The question (other sections) for the autistic adults was: “*What was the waiting time for you (*carers*: the adult) to get into the <residential, employment, education, financial, social support> service?”*. The question for the professionals was: *“To the best of your knowledge, what is the waiting time before an autistic adult can get into the following <residential, employment, education, financial, social support> services in the (geographical) area where you work now?”.*

**Supplementary Data 12**

***Service staff training***

| Autistic adult | | | | | | | | | | | | | | | | | | | | |
| --- | --- | --- | --- | --- | --- | --- | --- | --- | --- | --- | --- | --- | --- | --- | --- | --- | --- | --- | --- | --- |
|  | Residential service | | | | Employment service | | | | Adult educated service | | | | Financial service | | | | Social support service | | | |
|  | Yes | Some were and some were not | No | Don’t know | Yes | Some were and some were not | No | Don’t know | Yes | Some were and some were not | No | Don’t know | Yes | Some were and some were not | No | Don’t know | Yes | Some were and some were not | No | Don’t know |
|  | n=166 | | | | n=157 | | | | n=80 | | | | n=242 | | | | n=165 | | | |
| Seem knowledgeable about autism spectrum | 47 (28.31) | 57 (34.34) | 48 (28.92) | 14 (8.43) | 20 (12.74) | 48 (30.57) | 71 (45.22) | 18 (11.46) | 17 (21.25) | 20 (25.00) | 30 (37.50) | 13 (16.25) | 20 (8.26) | 59 (24.38) | 119 (49.17) | 44 (18.18) | 63 (38.18) | 52 (31.52) | 39 (23.64) | 11 (6.67) |
| Seem knowledgeable about the kinds of services and care that were available for adults with autism spectrum | 46 (27.71) | 50 (30.12) | 43 (25.90) | 27 (16.27) | 31 (19.75) | 44 (28.03) | 63 (40.13) | 19 (12.10) | 14 (17.50) | 21 (26.25) | 31 (38.75) | 14 (17.50) | 37 (15.29) | 59 (24.38) | 102 (42.15) | 44 (18.18) | 56 (33.94) | 48 (29.09) | 46 (27.88) | 15 (9.09) |
| Provide information about care and services that was easy to understand by the autistic adult | 36 (21.69) | 43 (25.90) | 69 (41.57) | 18 (10.84) | 29 (18.47) | 42 (26.75) | 76 (48.41) | 10 (6.37) | 17 (21.25) | 24 (30.00) | 30 (37.50) | 9 (11.25) | 38 (15.70) | 43 (17.77) | 126 (52.07) | 35 (14.46) | 58 (35.15) | 40 (24.24) | 53 (32.12) | 14 (8.48) |
| Carer | | | | | | | | | | | | | | | | | | | | |
|  | n=257 | | | | n=153 | | | | n=150 | | | | n=297 | | | | n=212 | | | |
| Seem knowledgeable about autism spectrum | 111 (43.19) | 82 (31.91) | 51 (19.84) | 13 (5.06) | 47 (30.72) | 48 (31.37) | 47 (30.72) | 11 (7.19) | 60 (40.00) | 56 (37.33) | 28 (18.67) | 6 (4.00) | 59 (19.87) | 106 (35.69) | 98 (33.00) | 34 (11.45) | 104 (49.06) | 64 (30.19) | 32 (15.09) | 12 (5.66) |
| Seem knowledgeable about the kinds of services and care that were available for adults with autism spectrum | 106 (41.25) | 67 (26.07) | 57 (22.18) | 27 (10.51) | 40 (26.14) | 41 (26.80) | 55 (35.95) | 17 (11.11) | 54 (36.00) | 46 (30.67) | 39 (26.00) | 11 (7.33) | 54 (18.18) | 92 (30.98) | 112 (37.71) | 39 (13.13) | 96 (45.28) | 56 (26.42) | 49 (23.11) | 11 (5.19) |
| Provide information about care and services that was easy to understand by the autistic adult | 78 (30.35) | 54 (21.01) | 98 (38.13) | 57 (10.51) | 31 (20.26) | 41 (26.80) | 61 (39.87) | 20 (13.07) | 49 (32.67) | 38 (25.33) | 49 (32.67) | 14 (9.33) | 35 (11.78) | 66 (22.22) | 158 (53.20) | 38 (12.79) | 78 (36.79) | 58 (27.36) | 64 (30.19) | 12 (5.66) |

*Note*. Values expressed as number of responders and frequencies (in parenthesis). The question was: *“At the organization, did the employees that worked with you* (carer: *the adult*) *to apply for <residential, employment, education, financial, social support> services:”*

**Supplementary Data 13**

***Good local models***

|  | Autistic adult | | | | Carer | | | | Professional | | | |
| --- | --- | --- | --- | --- | --- | --- | --- | --- | --- | --- | --- | --- |
|  | Yes | No | Don’t know | N | Yes | No | Don’t know | N | Yes | No | Don’t know | N |
| Residential service | 82 (17.48) | 278 (59.28) | 109 (23.24) | 469 | 153 (34.77) | 210 (47.73) | 77 (17.50) | 440 | 125 (72.67) | 22 (12.79) | 25 (14.53) | 172 |
| Employment service | 44 (9.52) | 302 (65.37) | 116 (25.11) | 462 | 83 (19.26) | 261 (60.56) | 87 (20.19) | 431 | 63 (55.26) | 30 (26.32) | 21 (18.42) | 114 |
| Adult education service | 46 (10.07) | 315 (68.93) | 96 (21.01) | 457 | 77 (18.33) | 255 (60.71) | 88 (20.95) | 420 | 40 (52.63) | 14 (18.42) | 22 (28.95) | 76 |
| Financial service | 45 (9.93) | 303 (66.89) | 105 (23.18) | 453 | 61 (14.63) | 253 (60.67) | 103 (24.70) | 417 | 19 (36.54) | 14 (26.92) | 19 (36.54) | 52 |
| Social support service | 64 (14.41) | 291 (65.54) | 89 (20.05) | 444 | 72 (17.43) | 238 (57.63) | 103 (24.94) | 413 | 56 (47.46) | 22 (18.64) | 40 (33.90) | 118 |

*Note*. Values expressed as number of responders and frequencies (in parenthesis). The question was: *“Do you know of a <residential, employment, education, financial, social> service for adults, in your area or elsewhere in your country, which works very well for adults on the autism spectrum?”.*
